# Ginger *(Zingiber officinale)* extract mediated green synthesis of silver nanoparticles and evaluation of their antioxidant activity and potential catalytic reduction activities with Direct Blue 15 or Direct Orange 26

**DOI:** 10.1371/journal.pone.0271408

**Published:** 2022-08-25

**Authors:** Daihua Hu, Tingting Gao, Xingang Kong, Na Ma, Jinhong Fu, Lina Meng, Xiaolong Duan, Ching Yuan Hu, Wang Chen, Zili Feng, Salman Latif

**Affiliations:** 1 School of Biological Sciences and Engineering, Shaanxi University of Technology, Hanzhong, Shaanxi, China; 2 Qinba State Key Laboratory of Biological Resources and Ecological Environment, Hanzhong, Shaanxi, China; 3 School of Materials Science and Engineering, Shaanxi University of Science and Technology, Xian, Shaanxi, China; 4 School of Physics and Telecommunication Engineering, Shaanxi University of Technology, Hanzhong, Shaanxi, China; 5 Department of Chemistry, College of Science, University of Ha’il, Ha’il, Saudi Arabia; Universiti Brunei Darussalam, BRUNEI DARUSSALAM

## Abstract

The green synthesis of silver nanoparticles (AgNPs) using a water extract of Ginger *(Zingiber officinale)* root by microwave irradiation and its antibacterial activities have been reported. However, AgNPs prepared from different parts of ginger root water or ethanol extract by ultrasound synthesis and their antioxidant activity and whether the biogenic could be used to catalyze the reduction of hazardous dye are unknown. This study concentrated on the facile green synthesis of AgNPs prepared from different parts (unpeeled ginger, peeled ginger, and ginger peel) of ginger root water or ethanol extract by the ultrasound-assisted method. We studied their antioxidant activity and catalytic degradation of hazardous dye Direct Orange 26 (DO26) and Direct Blue 15 (DB15). The surface plasmon resonance (SPR) peak of AgNPs was at 428–443 nm. The biogenic AgNPs were approximately 2 nm in size with a regular spherical shape identified from TEM analysis. The ethanol extracts of dried unpeeled ginger and peeled ginger, fresh peeled ginger and ginger peel. The *Z*. *officinale* AgNPs synthesized by dried unpeeled ginger ethanol extract showed the best antioxidant activity. Their scavenging activities were significantly better than BHT (*p* <0.05). The different parts of ginger extracts showed no catalytic degradation activities of DB15 and DO26. Still, the synthesized *Z*. *officinale* AgNPs exhibited good catalytic degradation activities, while their ability to catalytic degradation to DB15 was better than DO26. In the additive ratio of 3 mL DB15, 0.1 mL NaBH_4_ and 0.1 mL AgNPs, the degradation rates of DB15 (or DO26) at 15 min, 30 min and 60 min were only 1.8% (0.9%), 2.8% (1.4%) and 3.5% (1.6%) in the absence of AgNPs. When adding *Z*. *officinale* AgNPs prepared from dried ginger peel ethanol extract or fresh ginger peel water extract, the degradation rates of DB15 sharply increased to 97% and 93% after 30 min, respectively. In conclusion, ginger extract has good antioxidant properties. *Z*. *officinale* AgNPs biosynthesis from ginger extract exhibit excellent catalytic degradation activities, especially for the ginger peel extract. They have application value in the treatment of textile effluents and provide a new idea and method for the comprehensive development and utilization of ginger resources.

## Introduction

Silver nanoparticles (AgNPs) have received widespread attention due to their excellent properties like good photoelectrochemical activity, antimicrobial activity, anti-biofilms, electrical conductivity, and enzymatic activity [1–5]. The traditional preparation methods of AgNPs mainly include the physical and chemical methods [6–9]. As the potent reducing and capping agents frequently used in conventional methods are toxic to humans and the environment, green synthesis of AgNPs by a simple and eco-friendly method has gained more attention among researchers.

Biosynthesis has been preferred for synthesizing AgNPs to conventional methods using harmful reducing agents such as sodium borohydride, trisodium citrate, free aldehyde, ketone groups, and organic substances [10]. The biosynthesis of AgNPs using plant extracts instead of synthetic chemical reagents as the reducing, capping and stabilizing agents is one of the most commonly used green methods. Diverse plants such as *Givotia moluccana* [11], *Rhodiola rosea* [10, 12], *Corylus avellana* [2], Oyster mushroom [13], *Guettarda speciosa* [14], *Hagenia abyssinica* [15], *Syzygium cumini* [16], *Cassytha filiformis* [17], *Bryophyllum pinnatum* [18], and *Acacia cyanophylla* [19] have been successfully used to synthesize AgNPs. This approach has the advantages of being cost-effective, environmentally friendly, non-toxic and large-scale production of AgNPs. In addition, the AgNPs produced by plant extract could remain stable even after prolonged storage since it prevents the molecules from accumulating.

Azo dyes are synthetic dyes that generally contain one or more azo bonds (-N = N-) in their chemical structures [20]. Azo dyes are frequently used in printing and dyeing, textile, paper making, pharmaceutical and other industries. Due to the structural stability and high chromaticity, azo dyes are resistant to degradation. The discharge of textile effluent could cause serious environmental problems. Less than 50% of dyes used are not being fixed in the products and tend to be discharged into the environment with the effluent, especially azo dyes. They threaten the health of humans and eco-systems [21, 22]. Generally, the traditional physicochemical treatments of textile effluents are high-cost and may cause secondary pollution [23]. Therefore, there is an urgent need to develop more cost-effective, high-efficiency, and eco-friendly bioremediation technologies for textile effluents.

China is a major producer of ginger in the world. In 2020, the annual output of ginger in China reached 9.19 million tons, accounting for about 45% of the world’s total output. Gingerol is a general name for spicy ingredients in ginger, composed of gingerol, shogaol, zingerone and other substances. It is also the main functional factor of ginger with various pharmacological effects. Gingerol has a variety of pharmacological functions, including scavenging free radicals, anti-oxidation, anti-mutation, anti-tumor, antibacterial, anti-viral and immune regulation, and is widely used in seasoning food, medicine, cosmetics and other industries [24, 25].

Ginger peels are typically discarded during processing, resulting in a waste of resources. Thus, further exploring ginger peel usage, broadening the application value and scope, and promoting ginger’s comprehensive utilization deserve investigation. The green synthesis of AgNPs using a water extract of whole ginger *(Zingiber officinale)* root by microwave irradiation and its antibacterial activities have been reported [26–28]. Ultrasound-assisted extraction uses the cavity effect and mechanical effect generated by ultrasonic waves to promote the movement of medium molecules, improve their movement speed and increase the penetration of the medium to promote the extraction of active components in the organism. In addition, it has the advantages of short time, high yield and low yield of by-products [29, 30]. However, AgNPs prepared from different parts of ginger root water or ethanol extract by ultrasound synthesis and their antioxidant activity and whether the biogenic could be used to catalyze the reduction of hazardous dye are unknown. Therefore, this study concentrated on the facile green synthesis of AgNPs using ethanol or water extracts prepared from different parts (unpeeled ginger, peeled ginger, and ginger peel) of ginger roots by an ultrasound-assisted method. In addition, we studied their antioxidant activity and catalytic degradation of hazardous dye Direct Orange 26 (DO26) and Direct Blue 15 (DB15).

## Materials and methods

### Chemicals

All chemicals used were of analytical grade. Silver nitrate (AgNO_3_), sodium borohydride (NaBH_4_), butylated hydroxytoluene (BHT), and 2,2-diphenyl-1-picrylhydrazyl (DPPH) were purchased from Aladdin Industrial Corporation. Direct Orange 26 and Direct Blue 15 (98%) were purchased from Hubei Qifei Co., Ltd. Deionized water was prepared by a molecular ultrapure water system with an electrical resistivity of 18.25 MΩ·cm.

#### Preparation of fresh and dry root samples of *Z*. *officinale*

The fresh roots of *Z*. *officinale* were purchased from a local supermarket supplied by Shengjie Co. (Weifang, Shandong, China, 35.41°N and 108.10°E). Unpeeled ginger, peeled ginger, and ginger peel were taken separately and were cut off approximately 0.008 cm^3^. The various gingers were named fresh unpeeled ginger, fresh peeled ginger, and fresh ginger peel. They were sealed into bags separately and were stored at -4°C.

The obtained fresh unpeeled ginger, fresh peeled ginger, and fresh ginger peel were dried by hot air at 80°C for about 6–8 h, ground into powder (FW177 mill, Taisite Co., Tianjin, China) and screened by a 40-mesh sieve [10]. The obtained powders were named dry unpeeled ginger, dry peeled ginger, and dry ginger peel, respectively. They were sealed into bags separately and were stored at -20°C.

#### Preparation of extract from fresh or dry root of *Z*. *officinale*

Samples of fresh unpeeled ginger, peeled fresh ginger, and fresh ginger peel (5.0 g) were separately ultrasonically extracted with 50 mL of deionized water for 30 min at 60°C with a constant power of 200 W and a fixed frequency of 40 kHz (KQ500E Ultrasonic Cleaner, Kunshan, China). They were centrifuged at 3500 rpm for 10 min (5810R Centrifuge, Eppendorf Co., Hamburger, Germany), and the supernatant (water phase) was transferred into a vial [31]. The water phase was then collected and diluted with deionized water to 50 mL. The obtained solution was named fresh unpeeled ginger water extract, fresh peeled ginger water extract, and fresh ginger peel water extract were stored at -4°C, respectively. The ethanol extract of unpeeled fresh ginger, ethanol extracts of peeled fresh ginger and fresh ginger peel were prepared by a similar method, except the extracting solvent was replaced with 70% ethanol. All experiments were repeated three times.

The water content of unpeeled ginger, peeled ginger, and ginger peel root *Z*. *officinale* were (87.73 ± 2.41)%, (90.45 ± 3.26)%, and (87.36 ± 2.57)%, respectively. Samples of unpeeled dry ginger (0.61 g), peeled dry ginger (0.48 g), and dry ginger peel (0.63 g) were separately ultrasonically extracted with 50 mL of deionized water for 120 min at 60°C with a constant power of 200 W and a fixed frequency of 40 kHz (KQ500E Ultrasonic Cleaner, Kunshan, China). They were centrifuged at 3500 rpm for 10 min (5810R Centrifuge, Eppendorf Co., Hamburger, Germany), and the supernatant (water phase) was transferred into a vial [31]. The water phase was then collected and diluted with deionized water to 50 mL. The obtained solutions were named dry unpeeled ginger water extract, dry peeled ginger water extract, and dry ginger peel water extract. The ethanol extracts of unpeeled dry ginger, peeled dry ginger, and dry ginger peel were prepared using a similar method, except the extracting solvent was replaced with 70% ethanol. All experiments were repeated three times.

### Green synthesis of colloidal AgNPs using *Z*. *officinale* root extract

The green synthesis of colloidal AgNPs using *Z*. *officinale* was conducted using a previously reported method with minor modifications [10, 26]. First, one mL of the extracts of *Z*. *officinale* roots was added to 20 mL of silver nitrate solution (1.0 mmol/L). Then, the mixtures were separately ultrasound for 30 min at 60°C with a constant power of 200 W and a fixed frequency of 40 kHz (KQ500E Ultrasonic Cleaner, Kunshan, China).

### Characterization of AgNPs

#### Ultraviolet-visible spectrophotometry

An ultraviolet-visible spectrophotometer (UV-vis) (Mapada UV-3200, Shanghai) was used to confirm metal ions’ reduction into metal nanoparticles. For UV-visible spectroscopy studies, the synthesized colloidal AgNPs were scanned directly. The surface plasmon resonance peak was observed between 400 and 500 nm in the visible region. In addition, the absorbance was measured in the wavelength range between 200 and 800 nm [10, 32].

#### HR-TEM

Transmission electron microscopy (TEM) was performed on a Tecnai G2F20STWIN system (Thermo Fisher, Amsterdam, Netherlands) to evaluate the size and morphology at an accelerating voltage of 200 kV [33].

#### XRD

The prepared colloidal AgNPs were centrifuged at 10000 rpm /min for 10 min and then precipitated and dried at 60°C to obtain black solid particles. The solid particles were characterized by a D/max-2200 X-ray diffractometer (Rigku, Tokyo, Japan) using Cu Kα radiation (*λ* = 0.154 nm) [32, 33].

### Evaluation of the antioxidant activity of AgNPs

The antioxidant activity of AgNPs was determined by the DPPH radical scavenging method with minor modifications [10, 34]. Three mL of DPPH-ethanol solution (40 mg/L) and 200 μL of *Z*. *officinale* root extract or *Z*. *officinale* AgNPs, and BHT at the concentration of 1×10^−3^ mol/L were added into the centrifuge tube and then incubated for 1 h with oscillation at room temperature in the dark. The absorbance of the supernatant at 517 nm was determined. Ethanol solution was used as blank control, and BHT was used as a standard antioxidant. Antioxidant activity was measured by inhibition rate I % = [1-(A_0_-A_1_)/A_2_] × 100. In the formula, I is the inhibition ratio, A_0_ is the absorbance of the sample solution, A_1_ is the absorbance of the ethanol-sample solution, and A_2_ is the absorbance of the DPPH-ethanol solution. All experiments were repeated three times.

### Evaluation of the catalytic activity of *Z*. *officinale* root extract or *Z*. *officinale* AgNPs

The catalytic activity of AgNPs was determined by a published method with minor modifications [10, 32, 35]. The catalytic activity of *Z*. *officinale* root extract or synthesized *Z*. *officinale* AgNPs was determined based on the reductive degradation of DO26 and DB15 in the presence of NaBH_4_. Three milliliters of DO26 or DB15 (50 mg/L) were mixed with 0.1 mL NaBH_4_ (0.1 mol/L) and 0.1 mL *Z*. *officinale* root extract or synthesized *Z*. *officinale* AgNPs. The performance was monitored by recording UV-vis spectra variation over time (0, 3, 6, 9, 12, 15, 18, 21, 24, 27, 30, 40, 50, and 60 min). The control was also conducted without extract or AgNPs. The degradation rates for 15, 30, and 60 min were calculated. The degradation rate calculation formula was (1-A_1_/A_0_) *100, where A_0_ and A_1_ are the absorbance values at a wavelength of 603 nm (for DB15)/495 nm (for DO26) at 0 min, 15 min (or 30 min and 60 min), respectively. A pseudo-first-order reaction was assumed for the concentration of DO26 or DB15 to evaluate the reaction kinetics. The integrated form of first-order reaction is expressed as follows: ln A_*t*_/ A_*0*_ = -*k*t. A_*t*_ is the absorbance at time t, A_*0*_ is the absorbance at time 0, and *k* is the rate constant. All experiments were repeated three times.

### Statistical analysis

Data are expressed as the mean values ± standard deviation (SD) and were analyzed using one-way ANOVA. Duncan’s was used to determine significant differences between treatments. All statistical procedures were conducted using SPSS 22.0 for Windows. A significance level of *p<*0.05 was used.

## Results and discussion

### Spectroscopic and structural characterization of extract and synthesized AgNPs

**Fig 1:**
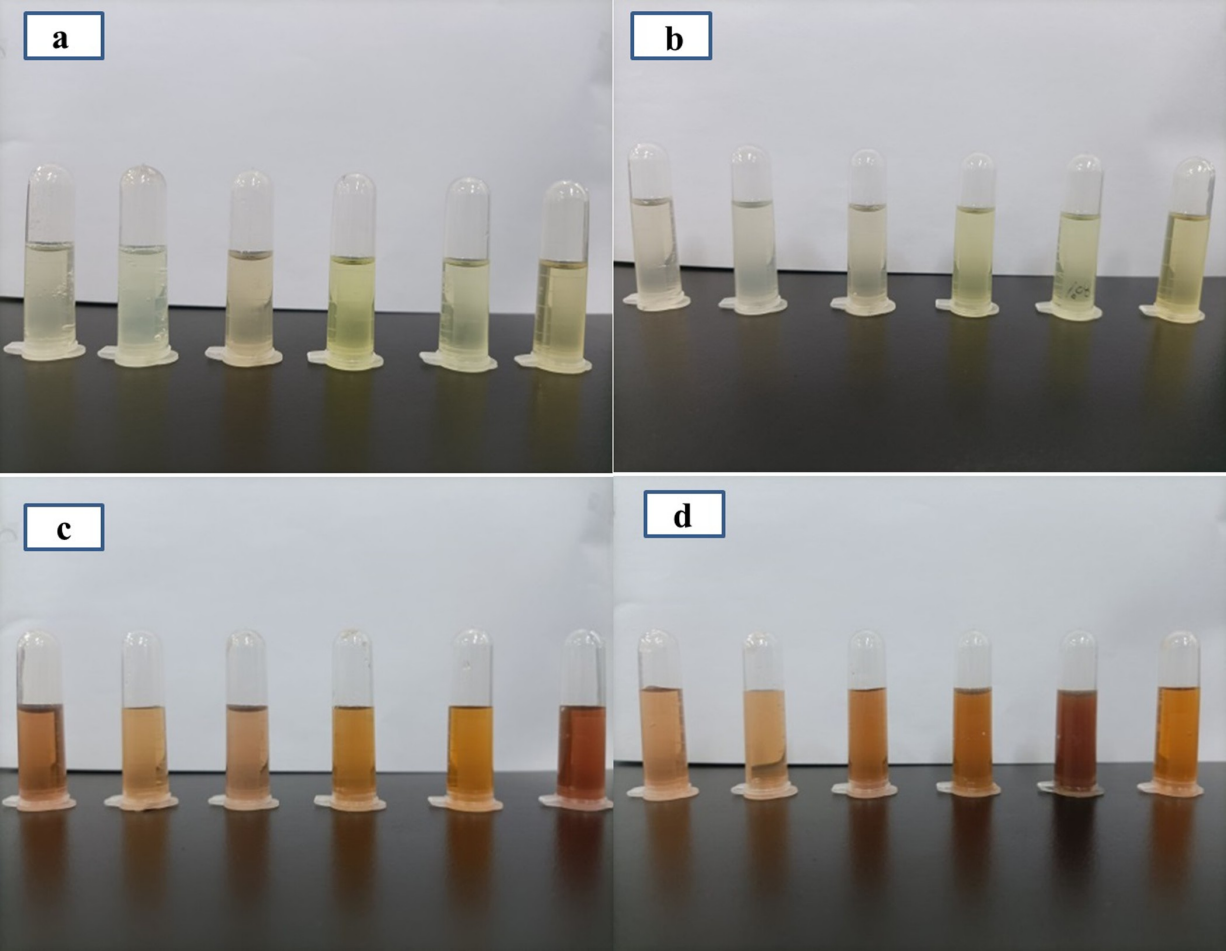
The solution appearance of *Z*. *officinale* root extract and synthesized *Z*. *officinale* AgNPs: (a) fresh ginger extract; (b) dry ginger extract; (c) fresh ginger extract AgNPs; (d) dry ginger extract AgNPs. Fig 1a and 1b from left to right was followed as the unpeeled ginger water extract, peeled ginger water extract, ginger peel water extract, unpeeled ginger ethanol extract, peeled ginger ethanol extract and ginger peel ethanol extract; Fig 1c and 1d from left to right are the unpeeled ginger water extract AgNPs, peeled ginger water extract AgNPs, ginger peel water extract AgNPs, unpeeled ginger ethanol extract AgNPs, peeled ginger ethanol extract AgNPs and ginger peel ethanol extract AgNPs.

**Fig 2:**
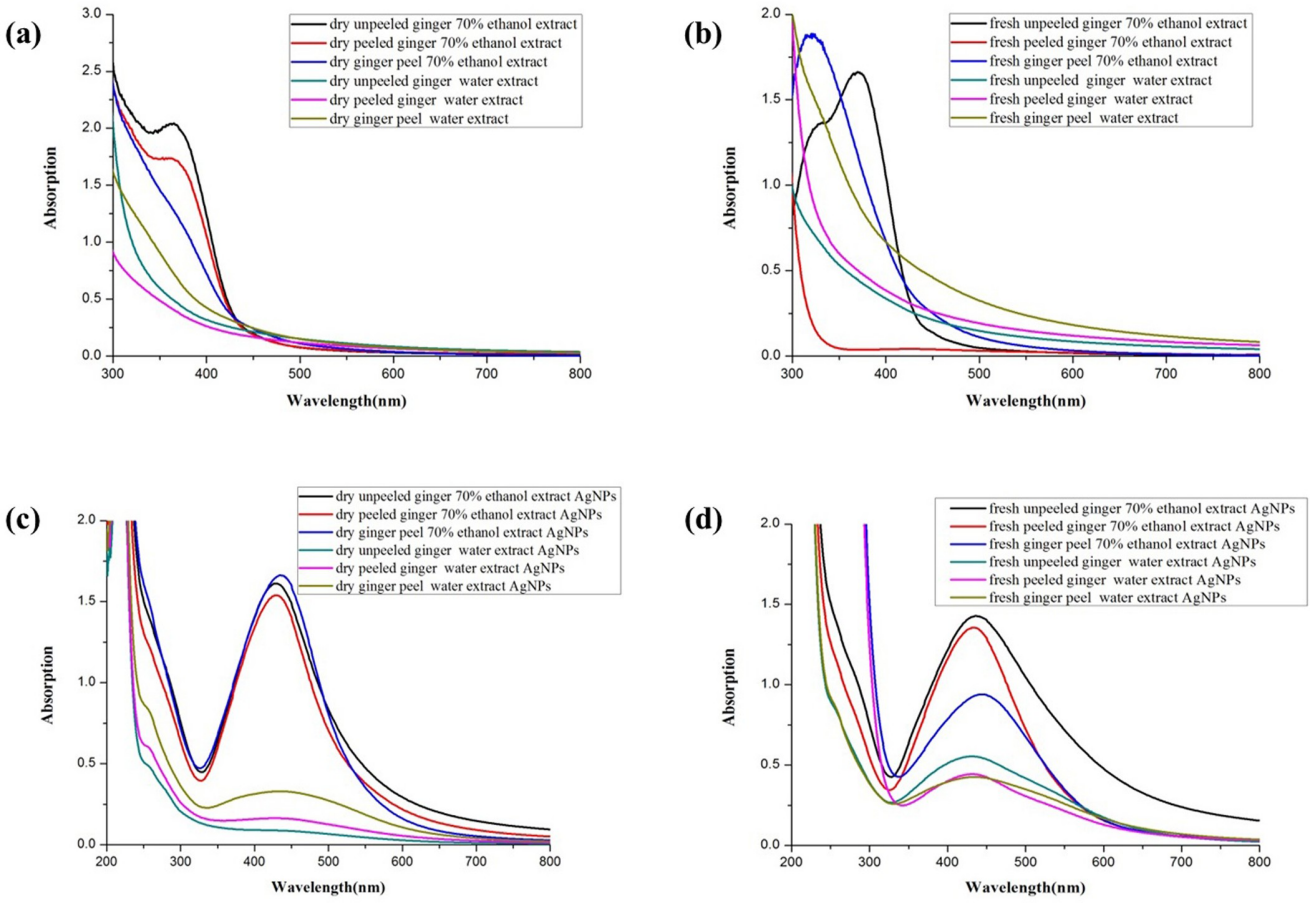
UV-vis spectra of the *Z*. *officinale* root extract and synthesized *Z*. *officinale* AgNPs: (a) dry ginger extract; (b) fresh ginger extract; (c) dry ginger extract AgNPs; (d) fresh ginger extract AgNPs.

**Fig 3:**
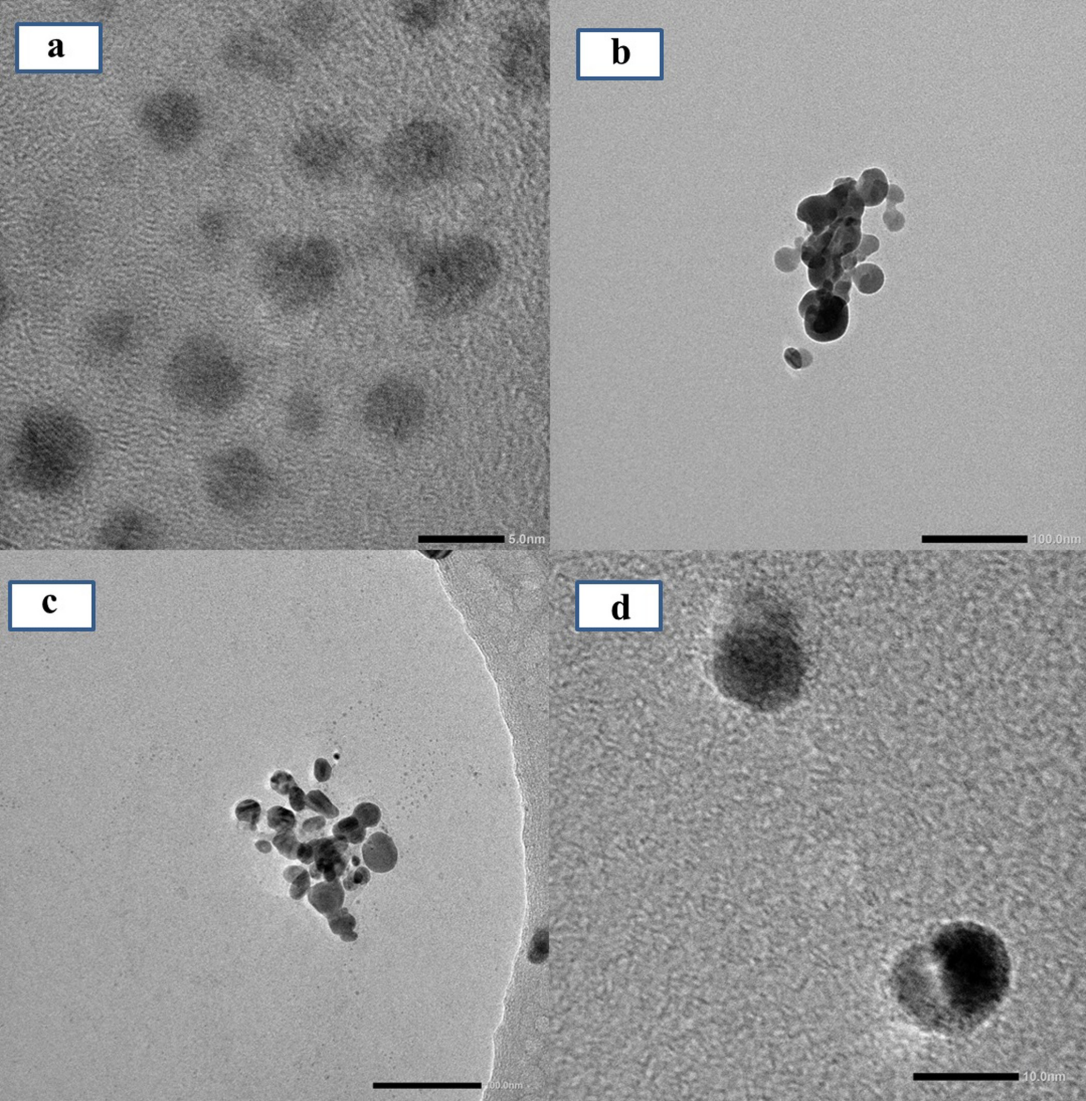
TEM images of the synthesized *Z*. *officinale* AgNPs under different magnifications: (a) AgNPs (synthesized using water extract of fresh ginger peel) at 5 nm; (b) AgNPs (synthesized using ethanol extract of fresh unpeeled ginger) at 100 nm; (c) AgNPs (synthesized using water extract of dry unpeeled ginger) at 100 nm; (d) AgNPs (synthesized using ethanol extract of dry ginger peel) at 10 nm.

**Fig 4:**
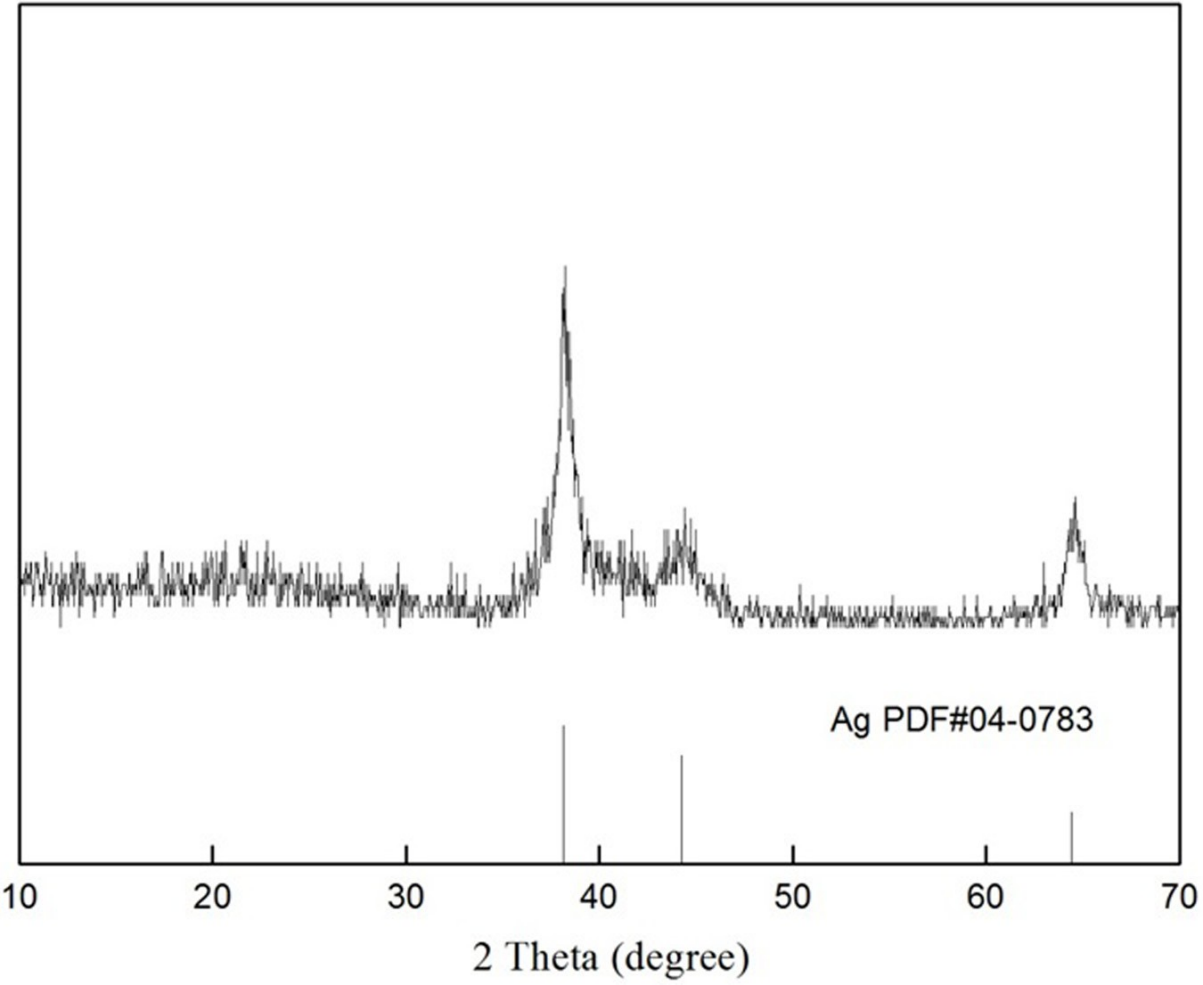
XRD patterns of synthesized *Z*. *officinale* AgNPs.

The extracts from different parts of fresh and dried ginger were nearly colorless to varying degrees of yellow transparent solutions. However, the color of ethanol extract was darker than that of water extract. After adding the extract of *Z*. *officinale* root to the colorless solution of silver nitrate, a visible color change was noticed from transparent to brownish red colloidal solution. The color of *Z*. *officinale* AgNPs prepared using ethanol extract was darker than water extract (Fig 1). The color change indicates the formation of colloidal silver particles, confirmed by ultraviolet absorption spectrum, TEM and XRD (Figs 2–4).

Fig 2 shows the absence of an SPR peak for the different ginger root extracts, but an SPR peak appeared around 428–443 nm. The SPR peak absorbance and peak width of *Z*. *officinale* AgNPs synthesized using various methods differed greatly. For example, the SPR peak absorbance of *Z*. *officinale* AgNPs synthesized using ethanol extract was higher (*p* <0.05) than water extract with smaller peak width. The SPR peak absorbance of *Z*. *officinale* AgNPs synthesized using ethanol extract of dry ginger was higher (*p* <0.05) than ethanol extract of fresh ginger with smaller peak width. The SPR peak absorbance of *Z*. *officinale* AgNPs synthesized using water extract of fresh ginger was higher (*p* <0.05) than water extract of fresh ginger with a smaller peak width. TEM results show that the AgNPs synthesized using water extract of fresh ginger peel had uniform particle size distribution, which was regular and spherical, and the particle size was about 2 nm. But the AgNPs synthesized using ethanol extract of fresh unpeeled ginger, and the AgNPs synthesized using water extract of the dry unpeeled ginger show a diameter of about 20 nm with a cluster distribution (Fig 3). XRD results show that there were three significant diffraction peaks at 2θ angles 38.116°, 44.277° and 64.426°, indicating that silver nanoparticles were successfully synthesized (Fig 4). The colloidal *Z*. *officinale* AgNPs synthesized from different methods show good stability, they could be maintained for several months.

### Evaluation of the antioxidant activity of *Z*. *officinale* root extract

Ginger extracts prepared by different methods significantly affected DPPH scavenging activity (*p* <0.05). For both fresh ginger and dry ginger, the antioxidant activity of ethanol extract from different parts of ginger was better than that of water extract (*p* <0.05). The ethanol extracts of unpeeled dry ginger, peeled dry ginger, peeled fresh ginger, and fresh ginger peel showed the best antioxidant activity. Their scavenging activities against DPPH were 65.6%, 63.0%, 57.6% and 54.3%, respectively, which were better (*p* <0.05) than that of BHT (42.3%). Nevertheless, the DPPH scavenging activity of water extract of unpeeled fresh ginger and ginger peel was lower (*p* <0.05) than that of BHT (Table 1). Ginger extract and its constituent components (such as 6-gingerol, 8-gingerol, 10-gingerol, and 6-shogaol) have been shown to have good antioxidant properties. This observation may be related to their hydroxyl groups and appropriate solubilizing side chains [36, 37]. In this study, ethanol extract of dry ginger exhibited better antioxidant properties than the BHT, and it can be used as a natural antioxidant.

**Table 1:** Antioxidant activity of *Z*. *officinale* root extract by DPPH radical scavenging (Mean ± SD, n = 3).

Type of ginger	Extraction solvent	^a^ DPPH inhibition rate (%)
unpeeled fresh ginger	water	24.83±1.34f
peeled fresh ginger		49.88±4.50bcd
fresh ginger peel		24.10±5.65f
unpeeled fresh ginger	70% ethanol	44.84±15.65cde
peeled fresh ginger		57.61±9.99ab
fresh ginger peel		54.30±0.70abc
unpeeled dry ginger	water	46.05±3.36bcde
peeled dry ginger		40.80±4.73de
dry ginger peel		36.96±1.03e
unpeeled dry ginger	70% ethanol	65.60±1.17a
peeled dry ginger		63.00±1.47a
dry ginger peel		43.85±1.68cde
BHT		42.30±9.32de

a: Different lowercase letters in the column indicate a significant difference (*p*<0.05).

### Evaluation of the antioxidant activity of synthesized *Z*. *officinale* AgNPs

The DPPH scavenging activity of *Z*. *officinale* AgNPs was lower than that of ginger root extract. *Z*. *officinale* AgNPs synthesized by different methods affected DPPH scavenging activity (*p* <0.05). For both fresh ginger and dry ginger, the antioxidant activity of *Z*. *officinale* AgNPs prepared using ethanol extract was better than that prepared using water extract (*p* <0.05). The *Z*. *officinale* AgNPs synthesized using ethanol extract of dry peeled ginger showed the best antioxidant activity, and its scavenging activities against DPPH was 64.9%, which was better (*p* <0.05) than that of BHT (42.3%). *Z*. *officinale* AgNPs synthesized using ethanol extract of fresh ginger peel also showed good antioxidant activity. Its scavenging activity against DPPH was 36.2%, and there was no difference (*p* >0.05) compared to BHT (Table 2). AgNPs colloids synthesized using *R*. *rosea* rhizome extract exhibited good antioxidant activity [10]. Its excellent antioxidant activity was attributed to antioxidant polyphenolic compounds such as phenylpropanoids, flavonoids and cinnamyl alcohol. In this study, *Z*. *officinale* AgNPs synthesized using ethanol extract of peeled dry ginger exhibited excellent antioxidant properties. Its excellent antioxidant activity most likely is attributed to the compounds such as gingerol, shogaol, and zingerone as reducing agents and stabilizers in the colloids.

**Table 2:** Antioxidant activity of *Z*. *officinale* AgNPs by DPPH radical scavenging (Mean ± SD, n = 3).

Type of ginger	Extraction solvent	^a^ DPPH inhibition rate (%)
unpeeled fresh ginger	water	6.95±0.97f
peeled fresh ginger		6.46±0.57f
fresh ginger peel		8.08±0.74f
unpeeled fresh ginger	70% ethanol	11.38±2.03ef
peeled fresh ginger		16.50±3.21e
fresh ginger peel		36.18±6.76bc
unpeeled dry ginger	water	17.70±1.35e
peeled dry ginger		17.76±1.33e
dry ginger peel		17.62±1.69e
unpeeled dry ginger	70% ethanol	28.57±0.31d
peeled dry ginger		64.87±5.33a
dry ginger peel		30.58±1.18cd
BHT	-	42.30±9.32b

a: Different lowercase letters in the column indicate a significant difference (*p*<0.05).

### Catalytic activity of colloidal AgNPs and ginger extract on the reduction of Direct Blue 15 by NaBH_4_

#### Catalytic activity of colloidal *Z*. *officinale* AgNPs synthesized from ginger extract on the reduction of Direct Blue 15 by NaBH_4_

**Fig 5:**
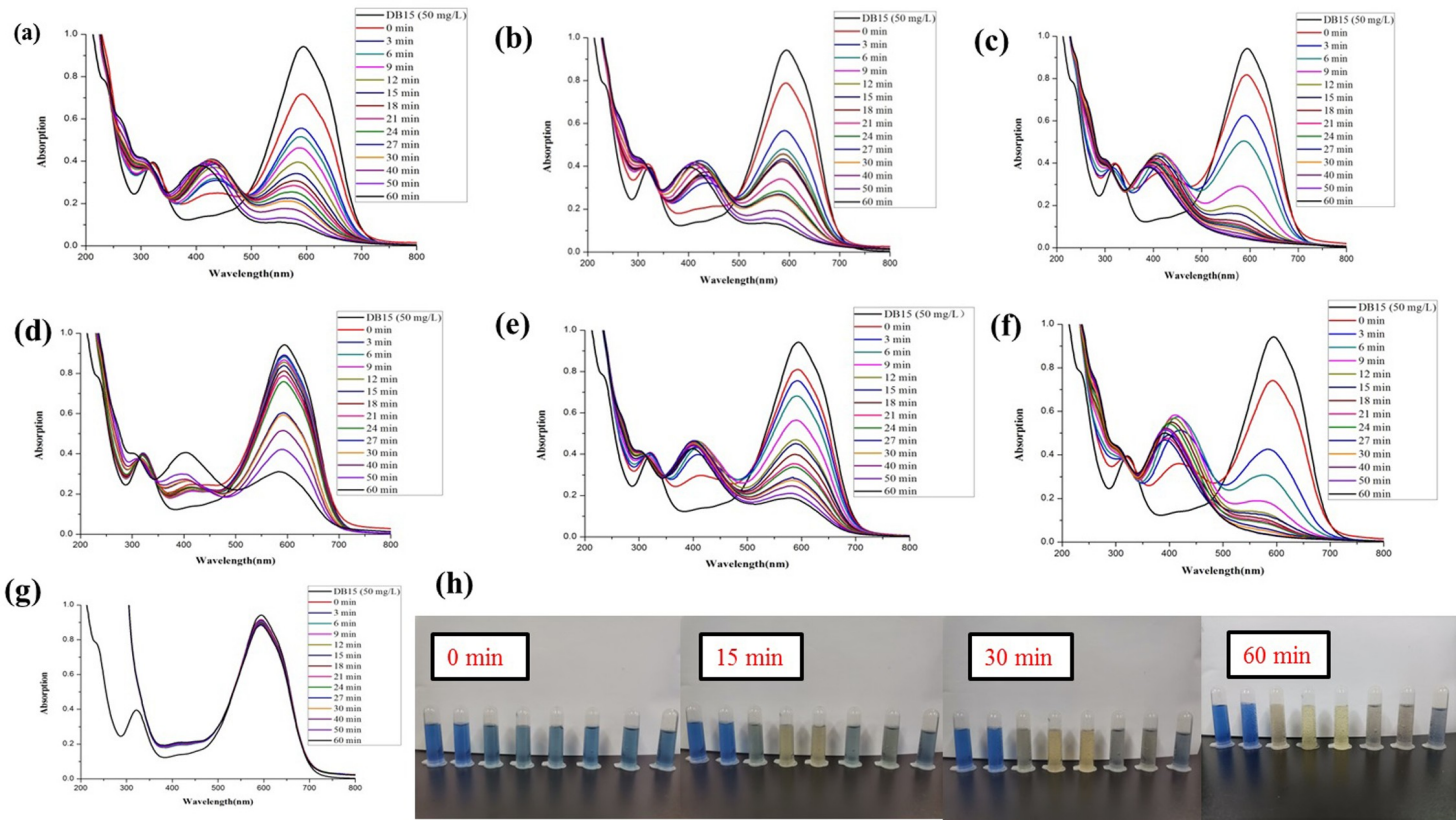
Comparison of the reductive degradation of Direct Blue 15 using different *Z*. *officinale* AgNPs synthesized from fresh ginger extracts: (a) unpeeled fresh ginger water extract AgNPs; (b) peeled fresh ginger water extract AgNPs; (c) fresh ginger peel water extract AgNPs; (d) unpeeled fresh ginger ethanol extract AgNPs; (e) peeled fresh ginger ethanol extract AgNPs; (f) fresh ginger peel ethanol extract AgNPs; (g) CK; (h) The solution appearance comparison of mixtures *Z*. *officinale* AgNPs and DB15 after reaction for 0, 15, 30 and 60 min. The solutions from left to right were followed as DB15 (50 mg/L), CK, unpeeled fresh ginger water extract AgNPs, peeled fresh ginger water extract AgNPs, fresh ginger peel water extract AgNPs, unpeeled fresh ginger ethanol extract AgNPs, peeled fresh ginger ethanol extract AgNPs, and fresh ginger peel ethanol extract AgNPs.

**Fig 6:**
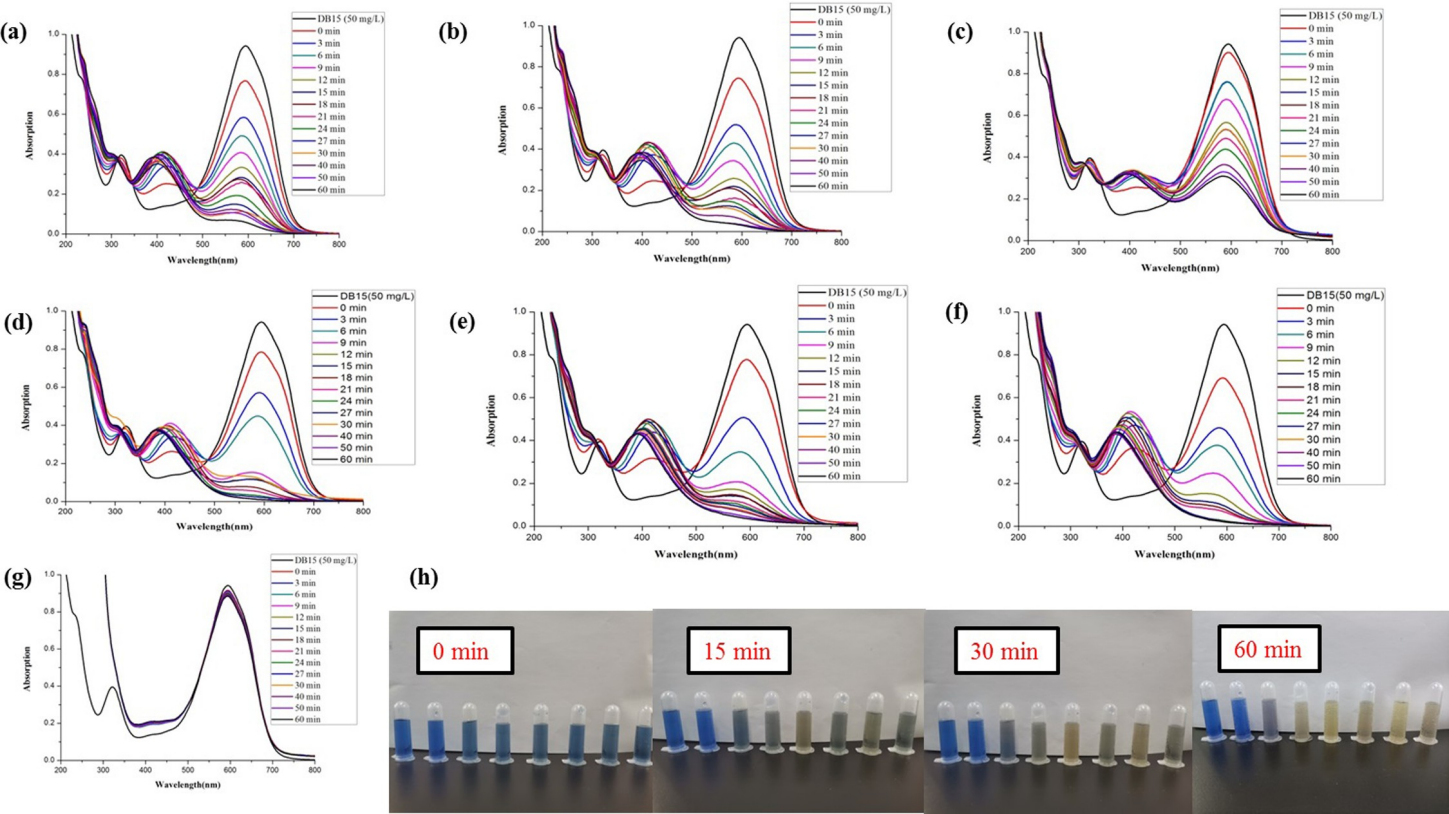
Comparison of the reductive degradation of Direct Blue 15 using different *Z*. *officinale* AgNPs synthesized from dry ginger extracts: (a) unpeeled dry ginger water extract AgNPs; (b) peeled dry ginger water extract AgNPs; (c) dry ginger peel water extract AgNPs; (d) unpeeled dry ginger ethanol extract AgNPs; (e) peeled dry ginger ethanol extract AgNPs; (f) dry ginger peel ethanol extract AgNPs; (g) CK; (h) The solution appearance comparison of mixtures *Z*. *officinale* AgNPs and DB15 after reaction for 0, 15, 30 and 60 min. The solutions from left to right were followed as DB15 (50 mg/L), CK, unpeeled dry ginger water extract AgNPs, peeled dry ginger water extract AgNPs, dry ginger peel water extract AgNPs, unpeeled dry ginger ethanol extract AgNPs, peeled dry ginger ethanol extract AgNPs, and dry ginger peel ethanol extract AgNPs.

The synthesized *Z*. *officinale* AgNPs colloids were directly used to compare the catalytic activities for the degradation of DB15 without further centrifugation. The maximum absorption of DB15 was at 603 nm, so the absorbance value at 603 nm was chosen to monitor the degradation of the azo dye. Without the addition of colloidal *Z*. *officinale* AgNPs, the characteristic peak of DB15 did not change (*p*>0.05) with the extension of time. However, the characteristic peak of DB15 decreased (*p*<0.05) continuously with the addition of *Z*. *officinale* AgNPs, which indicates that *Z*. *officinale* AgNPs had good catalytic abilities in the reduction of DB15 degradation. With the addition of *Z*. *officinale* AgNPs, a new peak near 430 nm appeared (Figs 5 and 6). The appearance of a new absorption peak was due to the addition of *Z*. *officinale* AgNPs, which indicates the presence of AgNPs.

In this research, the additive ratio was 3 mL DB15, 0.1 mL NaBH_4_ and 0.1 mL AgNPs. Generally, the degradation rate increased, and the degradation rate constant decreased with the increasing reaction time. In addition, the catalytic degradation effect of AgNPs prepared using the dry ginger extract was higher (*p*<0.05) than AgNPs prepared using fresh ginger extract. And AgNPs prepared using ethanol extract of ginger were better (*p*<0.05) than water extract (Figs 5 and 6).

The catalytic degradation of AgNPs prepared from ethanol extract of unpeeled dry ginger showed the best. The degradation rate of DB15 at 3, 6, 9, 12, 15, 18, 21, 24, and 27 min gradually increased from 28%, to 45%, 83%, 86%, 87%, 92%, 94%, 96%, and 97%, respectively. The degradation rate constants at 15 min and 30 min were higher than others *Z*. *officinale* AgNPs (Fig 6 and Table 3).

**Table 3:** Summary of degradation rate percentage and rate constant for degradation of DB15 catalyzed by *Z*. *officinale* AgNPs.

Catalyst	^a^ Degradation rate/100%			First-order rate constant/min^-1^		
	15 min	30 min	60 min	15 min	30 min	60 min
unpeeled fresh ginger water extract AgNPs	52.13±3.82d	73.03±2.54e	86.83±1.32e	0.0538	0.0489	0.0408
peeled fresh ginger water extract AgNPs	45.23±1.70e	67.95±1.32f	85.10±0.90f	0.0515	0.0428	0.0374
fresh ginger peel water extract AgNPs	81.69±1.48a	89.93±1.63c	94.50±1.07c	0.1191	0.0971	0.0691
unpeeled fresh ginger ethanol extract AgNPs	5.29±0.47g	31.82±1.47h	65.92±0.43h	0.0031	0.0093	0.0141
peeled fresh ginger ethanol extract AgNPs	43.70±1.88ef	66.28±1.86f	77.78±1.29g	0.0413	0.0395	0.0315
fresh ginger peel ethanol extract AgNPs	83.51±2.60a	93.33±1.48b	96.03±0.31bc	0.1484	0.109	0.0777
unpeeled dry ginger water extract AgNPs	61.09±3.24c	84.49±2.17d	92.52±0.76d	0.0726	0.0646	0.0515
peeled dry ginger water extract AgNPs	70.80±1.35b	86.14±2.30d	95.35±0.94c	0.0903	0.0754	0.064
dry ginger peel water extract AgNPs	40.40±1.58f	53.96±1.28g	66.21±1.44h	0.0369	0.0303	0.0235
unpeeled dry ginger ethanol extract AgNPs	84.52±2.89a	96.83±1.05a	98.27±0.59a	0.1501	0.1365	0.1016
peeled dry ginger ethanol extract AgNPs	82.94±1.23a	91.27±0.54bc	95.34±0.46c	0.1349	0.097	0.0695
dry ginger peel ethanol extract AgNPs	85.58±2.15a	96.82±0.39a	97.07±0.27ab	0.1361	0.1271	0.088
CK	1.75±0.07g	2.75±0.08i	3.54±0.14i	0.0013	0.0011	0.0008

a: Different lowercase letters in the same column indicate a significant difference (*p*<0.05).

The AgNPs prepared from water or ethanol extract of fresh ginger peel, and ethanol extract of the dry ginger (unpeeled ginger, peeled ginger and ginger peel) showed excellent catalytic activity of DB15 in a short time. For example, the degradation rates of DB15 could reach 86% after a reaction of 15 min (Figs 5 and 6 and Table 3). Furthermore, the AgNPs prepared from water extract of fresh ginger peel had higher (*p*<0.05) catalytic degradation of DB15 than dried ginger peel water extract (Figs 5 and 6 and Table 3).

For AgNPs prepared from fresh ginger extract, the catalytic degradation activity of ginger peel was better (*p*<0.05) than unpeeled and peeled ginger extract. Among them, AgNPs prepared from water and ethanol extract of ginger peel showed the best catalytic degradation activity for DB15; the degradation rate reached 83% after 15 min, and their catalytic degradation activity did not differ (*p*>0.05) (Table 3). While the AgNPs prepared using ethanol extract of unpeeled ginger exhibited a minimum catalytic activity (Fig 5 and Table 3).

For AgNPs prepared using dry ginger, the catalytic degradation activity from ethanol extract was better (*p*<0.05) than the water extract. While the catalytic degradation activity of AgNPs prepared from three ethanol extracts of ginger all showed excellent catalytic activity in DB15 in a short time, such as 15 min, the degradation rate reached the maximum of 85%. However, no difference (*p*>0.05) was observed among them (Table 3). In contrast, the catalytic degradation activity of the AgNPs prepared from different water extracts showed a significant difference (*p*<0.05), and the catalytic activities were in order of peeled ginger, unpeeled ginger and ginger peel. The catalytic activity of AgNPs synthesized using water extract of dry ginger was lower (*p*<0.05) in 15 min, but the degradation rate increased with the extension of time. For example, after a reaction of 60 min, the degradation rate of DB15 for AgNPs prepared from ethanol extract of unpeeled dry ginger reached a maximum of 98% (Fig 6 and Table 3).

The comparisons of degradation parameters for DB15 by AgNPs synthesized from plant extracts reported in the references are listed in Table 5. Compared to *R*. *rosea* AgNPs and C-AgNPs (chemically synthesized by reducing NaBH_4_), the amount of DB15 was lower, and the amount of NaBH_4_ and AgNPs was higher. Thus, the catalytic degradation ability of *R*. *rosea* AgNPs and C-AgNPs was much lower than synthesized *Z*. *officinale* AgNPs in this study. Compared to the tea polyphenol AgNPs [38], *Z*. *officinale* AgNPs exhibited excellent catalytic activity in a much shorter time (15min VS 80min) under the same ratio. Compared to the previous report, the AgNPs synthesized from *Z*. *officinale* showed higher catalytic degradation activity of DB15.

The catalytic reduction of DB15 and DO26 was usually explained in terms of the Langmuir- Hinshelwood model [32, 39]. Electron transfer plays a crucial role in the catalytic effect of AgNPs; that is, the AgNPs play a role in electron transfer between NaBH_4_ and dye, realizing the transfer of electrons between the provider (NaBH_4_) and the recipient (dye). When there is no catalyst, there is an electron transfer barrier between the reducing agent (NaBH_4_) and the dye, so the reduction could not be effective. Based on this, in catalytic reduction, NaBH_4_ and dye should be adsorbed on the surface of the AgNPs catalyst as much as possible to achieve more efficient electron transfer and reduce the degradation of dye. At the same time, the surface adsorption capacity of AgNPs is closely related to their particle size distribution, and particles with small particle size distribution tend to have stronger adsorption capacity. Therefore, the excellent catalytic activity of *Z*. *officinale* AgNPs in this study may be attributed to its smaller particle size.

#### Catalytic activity of ginger extract on the reduction of Direct Blue 15 by NaBH_4_

**Fig 7:**
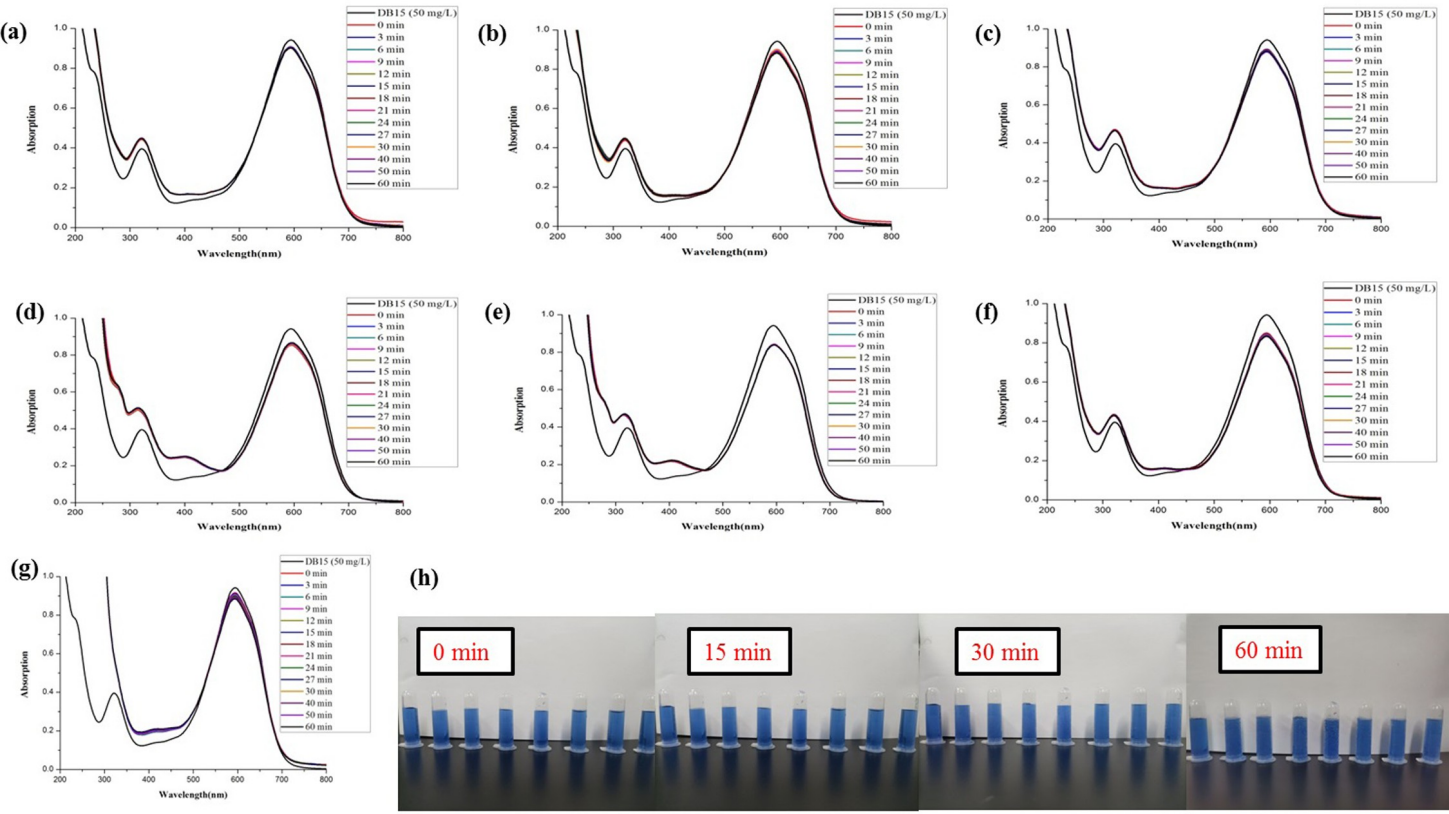
Comparison of the reductive degradation of Direct Blue 15 using different fresh ginger extracts: (a) unpeeled fresh ginger water extract; (b) peeled fresh ginger water extract; (c) fresh ginger peel water extract; (d) unpeeled fresh ginger ethanol extract; (e) peeled fresh ginger ethanol extract; (f) fresh ginger peel ethanol extract; (g) CK; (h) The solution appearance comparison of mixtures different fresh ginger extract and DB15 after reaction for 0, 15, 30 and 60 min. The solutions from left to right are DB15 (50 mg/L), CK, unpeeled fresh ginger water extract, peeled fresh ginger water extract, fresh ginger peel water extract, unpeeled fresh ginger ethanol extract, peeled fresh ginger ethanol extract, and fresh ginger peel ethanol extract.

**Fig 8:**
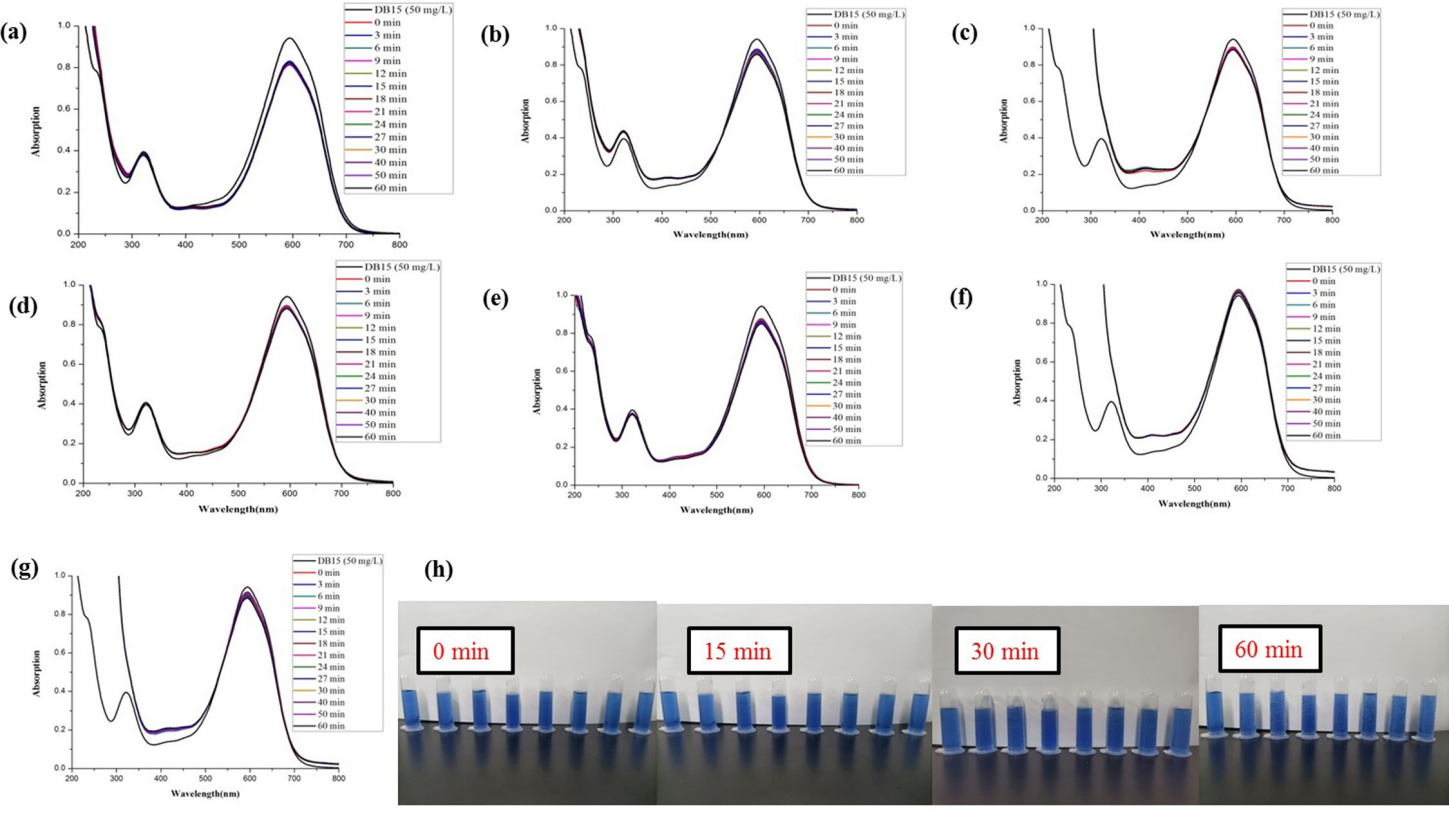
Comparison of the reductive degradation of Direct Blue 15 using different dry ginger extracts: (a) unpeeled dry ginger water extract; (b) peeled dry ginger water extract; (c) dry ginger peel water extract; (d) unpeeled dry ginger ethanol extract; (e) peeled dry ginger ethanol extract; (f) dry ginger peel ethanol extract; (g) CK; (h) The solution appearance comparison of mixtures different dry ginger extract and DB15 after reaction for 0, 15, 30 and 60 min. The solutions from left to right are DB15 (50 mg/L), CK, unpeeled dry ginger water extract, peeled dry ginger water extract, dry ginger peel water extract, unpeeled dry ginger ethanol extract, peeled dry ginger ethanol extract, and dry ginger peel ethanol extract.

As shown in Figs 7 and 8, the water and ethanol extract from different parts of fresh and dry ginger showed no catalytic degradation activities of DB15.

### Catalytic activity of colloidal AgNPs and ginger extract on the reduction of Direct Orange 26 by NaBH_4_

#### Catalytic activity of colloidal *Z*. *officinale* AgNPs synthesized from ginger extract on the reduction of Direct Orange 26 by NaBH_4_

**Fig 9:**
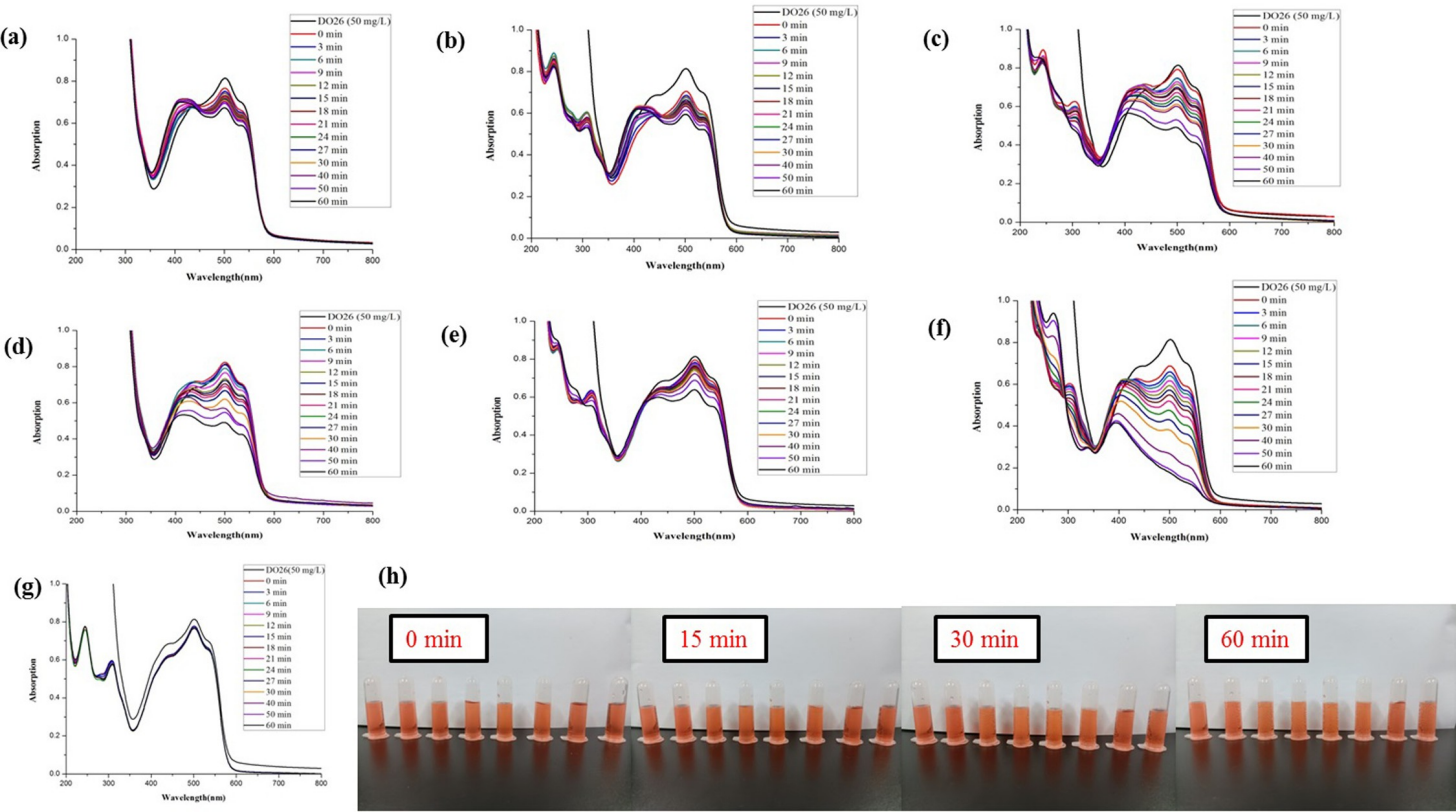
Comparison of the reductive degradation of Direct Orange 26 using different *Z*. *officinale* AgNPs synthesized from fresh ginger extracts: (a) unpeeled fresh ginger water extract AgNPs; (b) peeled fresh ginger water extract AgNPs; (c) fresh ginger peel water extract AgNPs; (d) unpeeled fresh ginger ethanol extract AgNPs; (e) peeled fresh ginger ethanol extract AgNPs; (f) fresh ginger peel ethanol extract AgNPs; (g) CK; (h) The solution appearance comparison of mixtures *Z*. *officinale* AgNPs and DO26 after reaction for 0, 15, 30 and 60 min. The solutions from left to right are DO26 (50 mg/L), CK, unpeeled fresh ginger water extract AgNPs, peeled fresh ginger water extract AgNPs, fresh ginger peel water extract AgNPs, unpeeled fresh ginger ethanol extract AgNPs, peeled fresh ginger ethanol extract AgNPs, and fresh ginger peel ethanol extract AgNPs.

**Fig 10:**
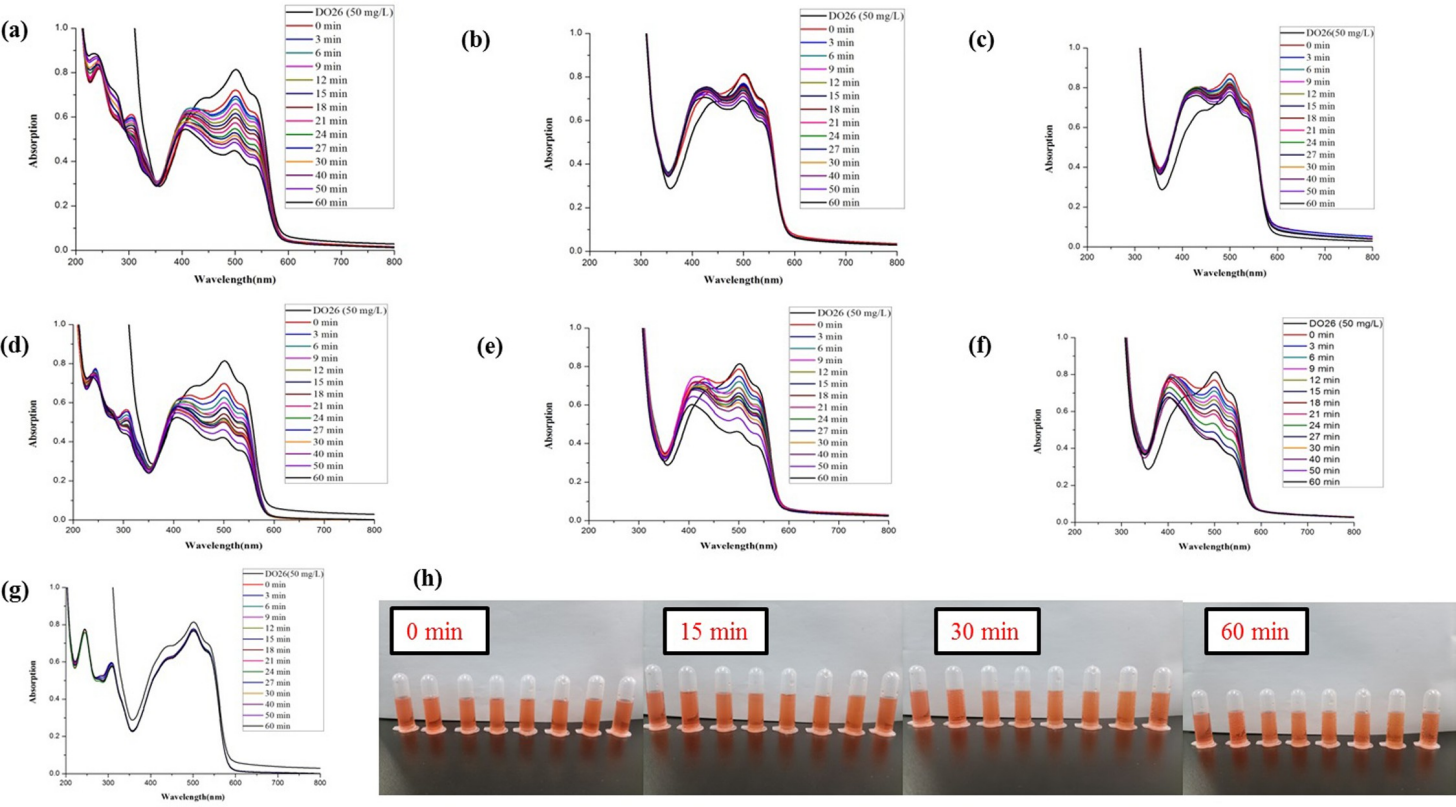
Comparison of the reductive degradation of Direct Orange 26 using different *Z*. *officinale* AgNPs synthesized from dry ginger extracts: (a) unpeeled dry ginger water extract AgNPs; (b) peeled dry ginger water extract AgNPs; (c) dry ginger peel water extract AgNPs; (d) unpeeled dry ginger ethanol extract AgNPs; (e) peeled dry ginger ethanol extract AgNPs; (f) dry ginger peel ethanol extract AgNPs; (g) CK; (h) The solution appearance comparison of mixtures *Z*. *officinale* AgNPs and DO26 after reaction for 0, 15, 30 and 60 min. The solutions from left to right are DO26 (50 mg/L), CK, unpeeled dry ginger water extract AgNPs, peeled dry ginger water extract AgNPs, dry ginger peel water extract AgNPs, unpeeled dry ginger ethanol extract AgNPs, peeled dry ginger ethanol extract AgNPs, and dry ginger peel ethanol extract AgNPs.

The synthesized *Z*. *officinale* AgNPs colloids were also directly used to compare the catalytic activities for the degradation of DO26 without further centrifugation. The maximum absorption of DO26 was at 494 nm, so the absorbance value at 494 nm was chosen to monitor the degradation of the azo dye. Without the addition of colloidal *Z*. *officinale* AgNPs, the characteristic peak of DO26 did not change (*p*>0.05) with the extension of time. However, the characteristic peak of DO26 decreased (*p*<0.05) continuously with the addition of *Z*. *officinale* AgNPs, which indicated that *Z*. *officinale* AgNPs had good catalytic abilities in the reduction of DO26 degradation (Figs 9 and 10). With the addition of *Z*. *officinale* AgNPs, a new peak near 430 nm appeared. The appearance of a new absorption peak was due to adding *Z*. *officinale* AgNPs, which indicates the presence of AgNPs.

In this research, the addition ratio of DO26 (50 mg/L), NaBH_4_ (0.1 mol/L) and AgNPs was *V* (DO26): *V*(NaBH_4_): *V*(AgNPs) = 3:0.1:0.1. In general, the catalytic degradation activity of the synthesized *Z*. *officinale* AgNPs for DO26 was lower than that for DB15 under the same conditions. The catalytic degradation activity of *Z*. *officinale* AgNPs prepared by ginger peel extract for DO26 was higher (*p*<0.05) than that of unpeeled ginger and peeled ginger. Among them, the catalytic degradation activity of *Z*. *officinale* AgNPs prepared from fresh ginger peel was superior to dry ginger peel, and the ethanol extract of ginger peel was better (*p*<0.05) than that of water extract of ginger peel (Figs 9 and 10 and Table 4).

**Table 4:** Summary of degradation rate percentage and rate constant for degradation of DO26 catalyzed by *Z*. *officinale* AgNPs.

Catalyst	^a^ Degradation rate/100%			First-order rate constant/min^-1^		
	15 min	30 min	60 min	15 min	30 min	60 min
unpeeled fresh ginger water extract AgNPs	4.39±0.33g	7.13±0.24gh	11.32±0.66i	0.0034	0.0027	0.0023
peeled fresh ginger water extract AgNPs	5.54±0.29f	8.73±0.21f	14.58±0.90h	0.0041	0.0035	0.0029
fresh ginger peel water extract AgNPs	11.17±0.28e	21.86±0.40e	23.99±0.83f	0.0087	0.0081	0.0078
unpeeled fresh ginger ethanol extract AgNPs	11.61±0.85e	23.69±1.16d	38.95±1.27cd	0.0087	0.0086	0.0085
peeled fresh ginger ethanol extract AgNPs	3.72±0.19g	6.40±0.38h	18.83±0.49g	0.0029	0.0025	0.0029
fresh ginger peel ethanol extract AgNPs	15.98±0.54bc	43.02±1.03a	71.99±1.12a	0.0120	0.0156	0.0206
unpeeled dry ginger water extract AgNPs	13.89±0.75d	27.70±0.83c	36.82±0.60e	0.0104	0.0109	0.0089
peeled dry ginger water extract AgNPs	6.20±0.15f	9.32±0.31f	13.95±0.26h	0.0055	0.0037	0.0029
dry ginger peel water extract AgNPs	5.63±0.36f	8.26±0.14fg	11.85±0.37i	0.0047	0.0034	0.0026
unpeeled dry ginger ethanol extract AgNPs	17.20±0.30a	26.79±0.46c	38.45±0.78d	0.0149	0.0124	0.0095
peeled dry ginger ethanol extract AgNPs	15.26±0.42c	20.87±1.07e	39.87±1.15bc	0.0139	0.0092	0.0084
dry ginger peel ethanol extract AgNPs	16.32±0.48b	39.39±1.12b	41.11±0.42b	0.0126	0.0151	0.0119
CK	0.86±0.04h	1.36±0.08i	1.55±0.06j	0.0008	0.0005	0.0004

a: Different lowercase letters in the same column indicate a significant difference (*p*<0.05).

The degradation rate increased, and the degradation rate constant decreased with the increasing reaction time. *Z*. *officinale* AgNPs prepared from the ethanol extract of fresh ginger peel showed the maximum catalytic degradation activity for DO26, and the degradation rate reached 72%. The *Z*. *officinale* AgNPs prepared from the ethanol extract of dry ginger peel also showed good catalytic degradation activity for DO26, but the degradation rate only reached 41% (Figs 9 and 10 and Table 4).

The comparisons of degradation parameters for DO26 by AgNPs synthesized from plant extracts reported in the references are listed in Table 5. Compared to *R*. *rosea* AgNPs and C-AgNPs [10], the amount of DO26 was lower. On the other hand, the amount of NaBH_4_ and AgNPs was higher. Thus the catalytic degradation ability of *R*. *rosea* AgNPs and C-AgNPs was much higher than synthesized *Z*. *officinale* AgNPs in this study. Compared to the *Eucommia ulmoides* AgNPs [40] and grape seed AgNPs [35] under the same ratio, the AgNPs synthesized from *Z*. *officinale* showed poor catalytic degradation activity for DO26 (Table 5). The *Z*. *officinale* AgNPs prepared using ginger extract have some catalytic degradation activity for DO26 but are lower than the previous report. Ginger extract has a smaller particle size, but its catalytic activity for DB15 and DO26 varied. However, the specific mechanism needs to be further studied.

**Table 5:** Comparisons of degradation parameters for DB15/DO26 by AgNPs synthesized from different plant extracts between reported references.

catalyst	dye	addition ratio /*V*(DB15):*V*(NaBH_4_): *V*(AgNPs)	average diameter /nm	time/min	degradationrate/%	degradation constant /min^-1^	reference
*R*. *rosea* AgNPs	DB15	1:2:0.2	10	18	82.3	0.128	[10]
C-AgNPs	DB15	1:2:0.2	20	18	37.9	0.0374	
Control	DB15	1:2:0.2	-	18	21.3	0.0165	
tea polyphenol AgNPs	DB15	3:0.1:0.1	68	80	91	0.0339	[38]
Control	DB15	3:0.1:0.1	-	80	35	0.0055	
*Z*. *officinale* AgNPs	DB15	3:0.1:0.1	2	15	87.3	0.1361	this research
	DB15	3:0.1:0.1	2	30	97.1	0.1271	
	DB15	3:0.1:0.1	2	60	97.4	0.088	
Control	DB15	3:0.1:0.1	-	15	1.8	0.0013	
	DB15	3:0.1:0.1	-	30	2.8	0.0011	
	DB15	3:0.1:0.1	-	60	3.7	0.0008	
*R*. *rosea* AgNPs	DO26	1:2:0.2	10	18	72.8	0.0818	[10]
C-AgNPs	DO26	1:2:0.2	20	18	40.5	0.0359	
Control	DO26	1:2:0.2	-	18	21.1	0.0138	
*E*. *ulmoides* AgNPs	DO26	3:0.1:0.1	40	30	93.2	0.088	[40]
Control	DO26	3:0.1:0.1	-	30	NR	0.0018	
grape seed AgNPs	DO26	3:0.1:0.1	54.8	18	82.6	0.1003	[35]
Control	DO26	3:0.1:0.1	-	18	NR	NR	
*Z*. *officinale* AgNPs	DO26	3:0.1:0.1	2	15	16.5	0.012	this research
	DO26	3:0.1:0.1	2	30	43.7	0.0156	
	DO26	3:0.1:0.1	2	60	72.9	0.0206	
Control	DO26	3:0.1:0.1	-	15	0.9	0.0008	
	DO26	3:0.1:0.1	-	30	1.4	0.0005	
	DO26	3:0.1:0.1	-	60	1.6	0.0004	

#### Catalytic activity of ginger extract on the reduction of Direct Orange 26 by NaBH_4_

**Fig 11:**
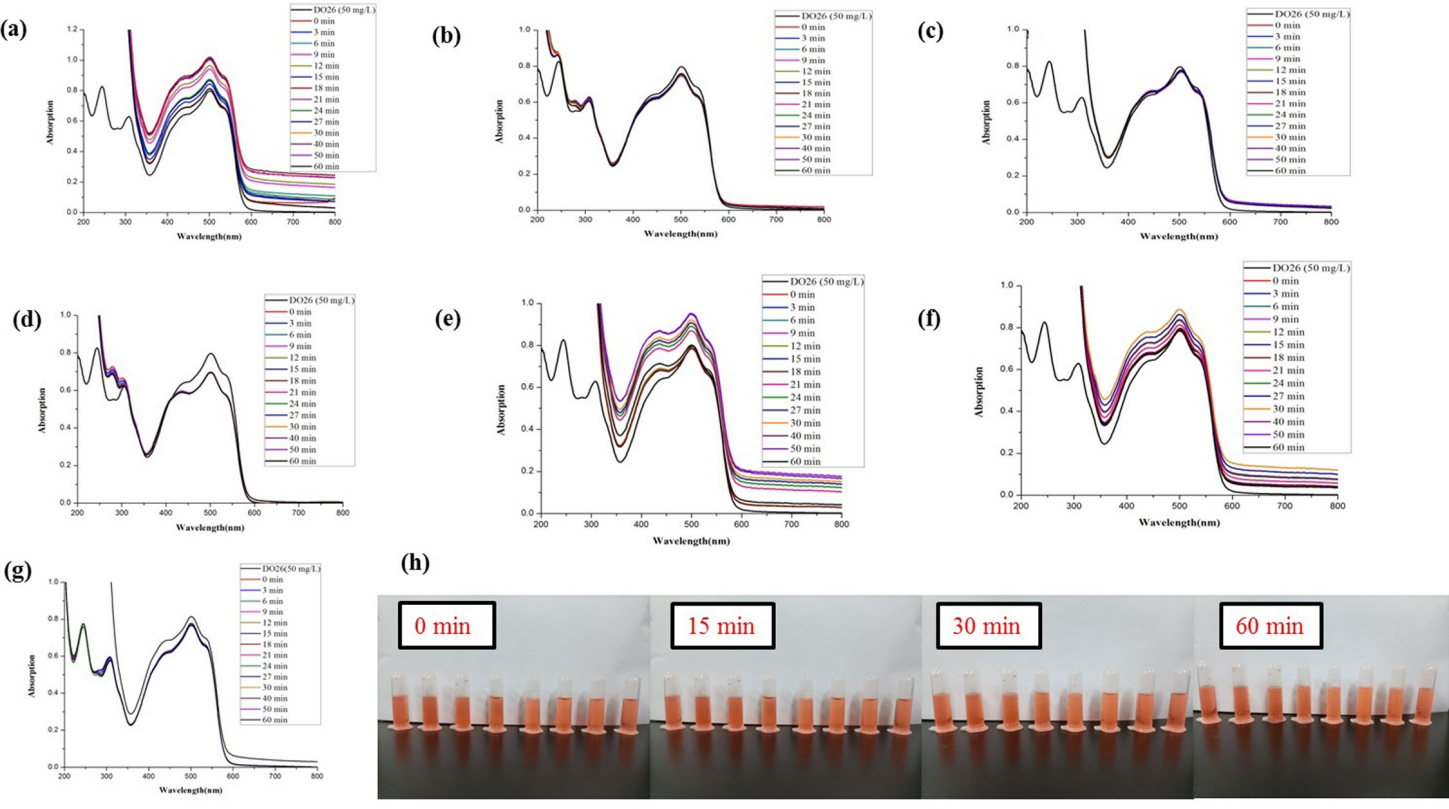
Comparison of the reductive degradation of Direct Orange 26 using different fresh ginger extracts: (a) unpeeled fresh ginger water extract; (b) peeled fresh ginger water extract; (c) fresh ginger peel water extract; (d) unpeeled fresh ginger ethanol extract; (e) peeled fresh ginger ethanol extract; (f) fresh ginger peel ethanol extract; (g) CK; (h) The solution appearance comparison of mixtures different fresh ginger extract and DO26 after reaction for 0, 15, 30 and 60 min. The solutions from left to right are DO26 (50 mg/L), CK, unpeeled fresh ginger water extract, peeled fresh ginger water extract, fresh ginger peel water extract, unpeeled fresh ginger ethanol extract, peeled fresh ginger ethanol extract, and fresh ginger peel ethanol extract.

**Fig 12:**
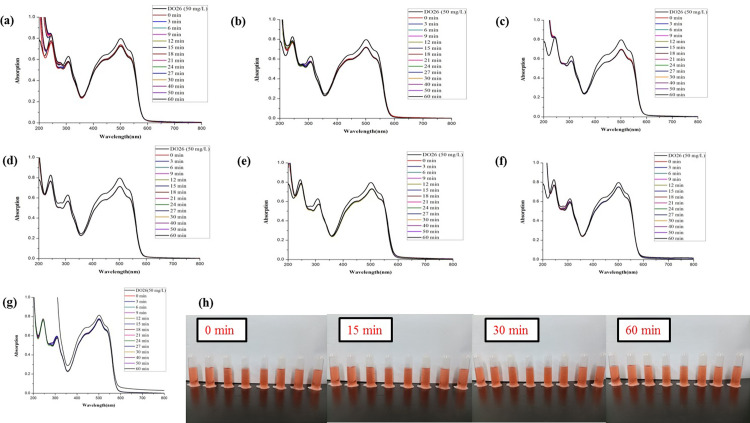
Comparison of the reductive degradation of Direct Orange 26 using different dry ginger extracts: (a) unpeeled dry ginger water extract; (b) peeled dry ginger water extract; (c) ginger dry peel water extract; (d) unpeeled dry ginger ethanol extract; (e) peeled dry ginger ethanol extract; (f) dry ginger peel ethanol extract; (g) CK; (h) The solution appearance comparison of mixtures different dry ginger extract and DO26 after reaction for 0, 15, 30 and 60 min. The solutions from left to right are DO26 (50 mg/L), CK, unpeeled dry ginger water extract, peeled dry ginger water extract, dry ginger peel water extract, unpeeled dry ginger ethanol extract, peeled dry ginger ethanol extract, and dry ginger peel ethanol extract.

As shown in Figs 11 and 12, the water and ethanol extracts from different parts of fresh and dried ginger had no catalytic degradation activities of DO26.

## Conclusions

This study concentrated on the facile green synthesis of AgNPs using ethanol or water extracts from different parts (unpeeled ginger, peeled ginger, and ginger peel) of the ginger root by the ultrasound-assisted method. We studied their antioxidant activity and catalytic degradation of hazardous dye DB15 and DO26. The surface plasmon resonance (SPR) peak of AgNPs was at 428–443 nm. The colloidal *Z*. *officinale* AgNPs synthesized from different ginger extract show good stability. The biogenic *Z*. *officinale* AgNPs synthesized using water extract of fresh ginger peel were approximately 2 nm in size with a regular spherical shape identified from TEM analysis. But the AgNPs synthesized using ethanol extract of fresh unpeeled ginger, or water extract of the dry unpeeled ginger show a diameter of about 20 nm with a cluster distribution. The ethanol extracts of unpeeled dry ginger and peeled dry ginger, peeled fresh ginger, fresh ginger peel, and the *Z*. *officinale* AgNPs synthesized by ethanol extract of unpeeled dry ginger showed the best antioxidant activity. Their scavenging activities were significantly better than BHT (*p* <0.05). The different parts of ginger extracts showed no catalytic degradation activities of DB15 and DO26. Still, the synthesized *Z*. *officinale* AgNPs exhibited good catalytic degradation activities, while their ability to catalytic degradation to DB15 was better than DO26. In the additive ratio of 3 mL DB15, 0.1 mL NaBH_4_ and 0.1 mL AgNPs, the degradation rates of DB15 (or DO26) at 15 min, 30 min and 60 min were only 1.8% (0.9%), 2.8% (1.4%) and 3.5% (1.6%) in the absence of AgNPs. When adding *Z*. *officinale* AgNPs prepared from ethanol extract of unpeeled dry ginger or water extract of fresh ginger peel, the degradation rates of DB15 sharply increased to 97% and 93% after 30 min, respectively. In conclusion, ginger extract has good antioxidant properties. *Z*. *officinale* AgNPs biosynthesis from ginger extract exhibit excellent catalytic degradation activities, especially for the ginger peel extract. They have application value in the treatment of textile effluents and provide a new idea and method for the comprehensive development and utilization of ginger resources.

## Supporting information

S1 File (ZIP)
