# Supplementary figures and images for: Ginger (Zingiber officinale) extract mediated green synthesis of silver nanoparticles and evaluation of their antioxidant activity and potential catalytic reduction activities with Direct Blue 15 or Direct Orange 26
Source: PLoS One. 2022 Aug 25;17(8):e0271408. doi: 10.1371/journal.pone.0271408 (PMC9409512; doi:10.1371/journal.pone.0271408)

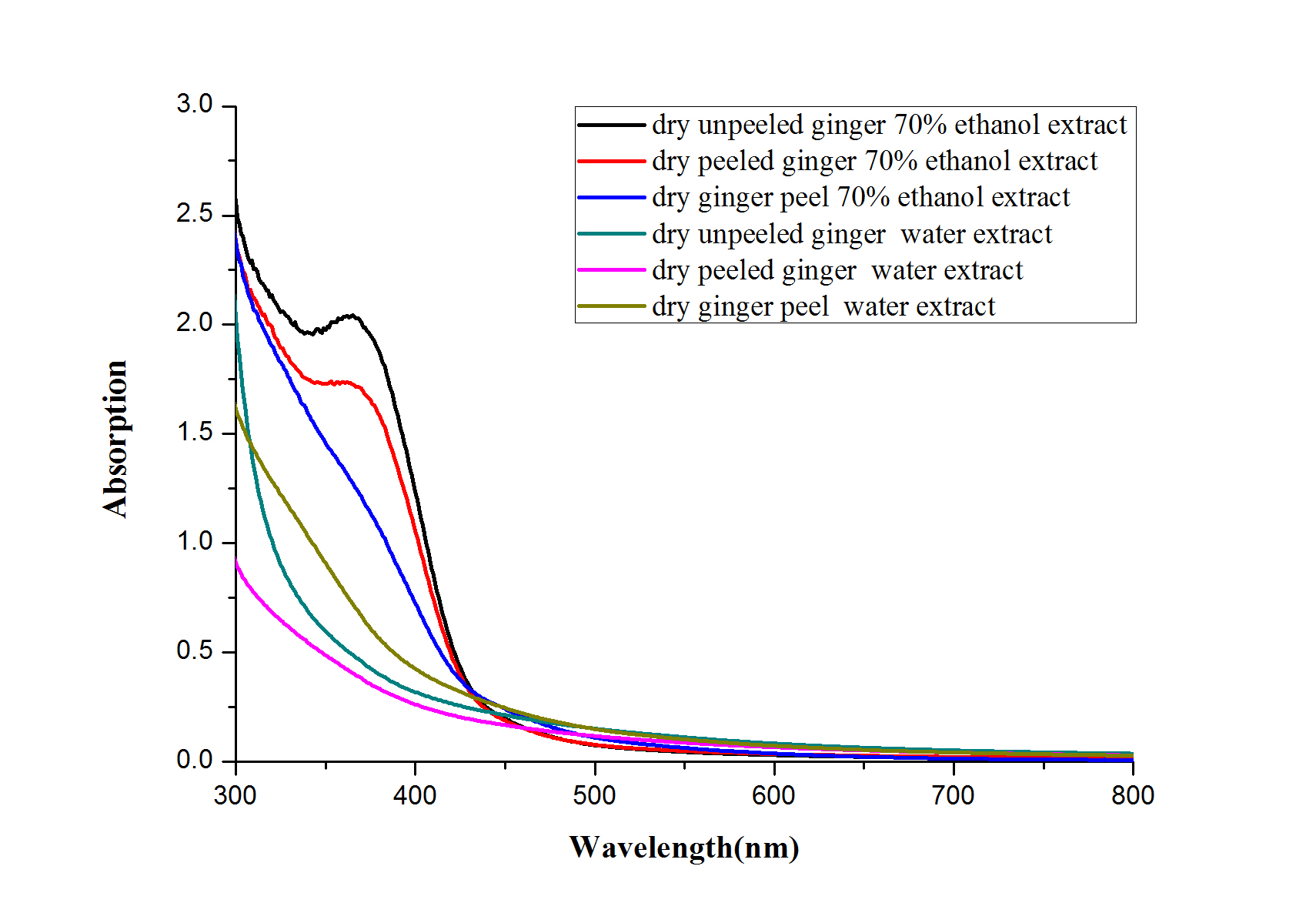

Supplement: S1 File — (ZIP) [file pone.0271408.s001.zip › supporting informations-PONE-D-22-11812/Fig 2-extract+AgNPs-UV/╕╔╓╞╠ß╚í╬∩.jpg]

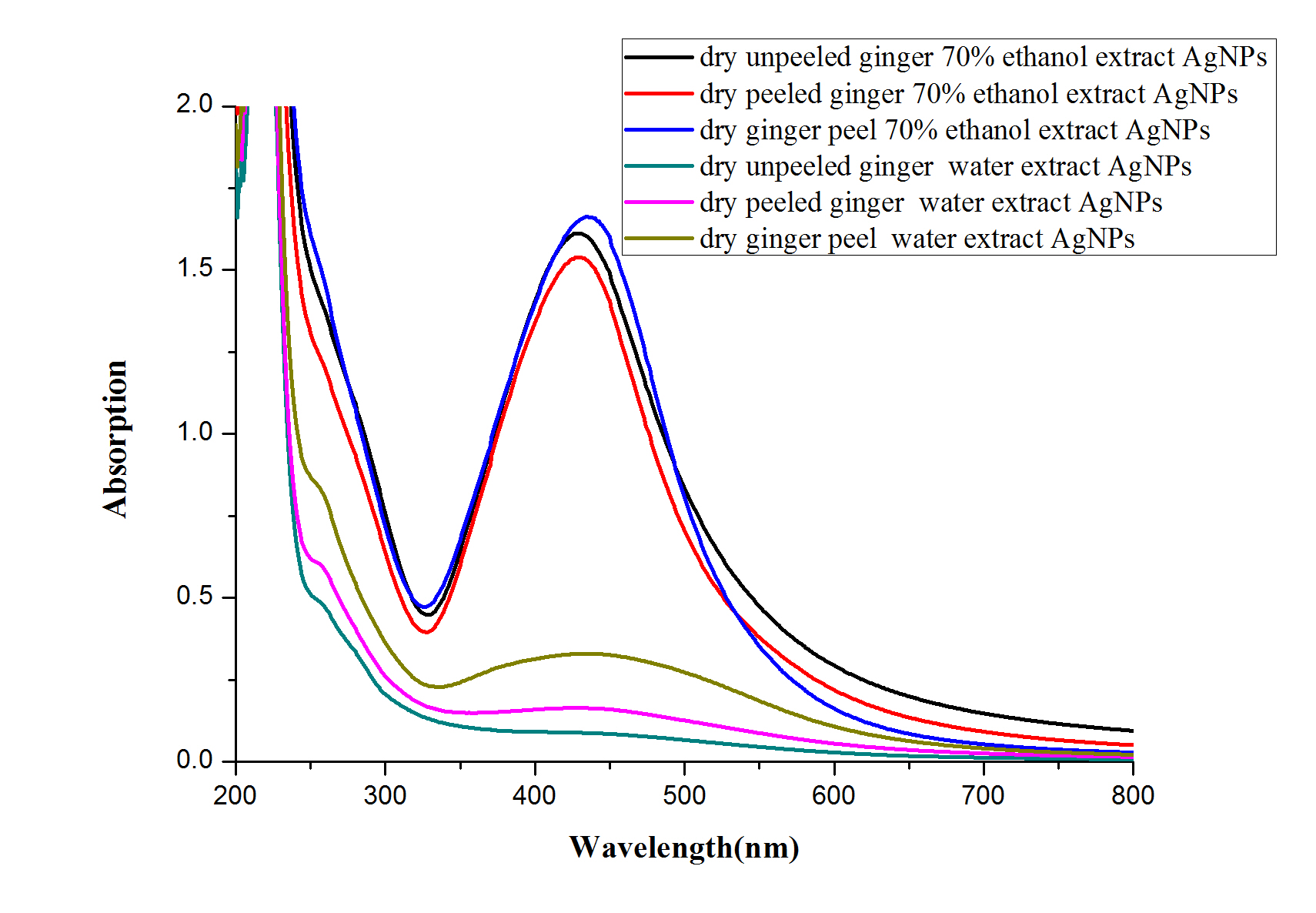

Supplement: S1 File — (ZIP) [file pone.0271408.s001.zip › supporting informations-PONE-D-22-11812/Fig 2-extract+AgNPs-UV/╕╔╓╞─╔├╫╥°.jpg]

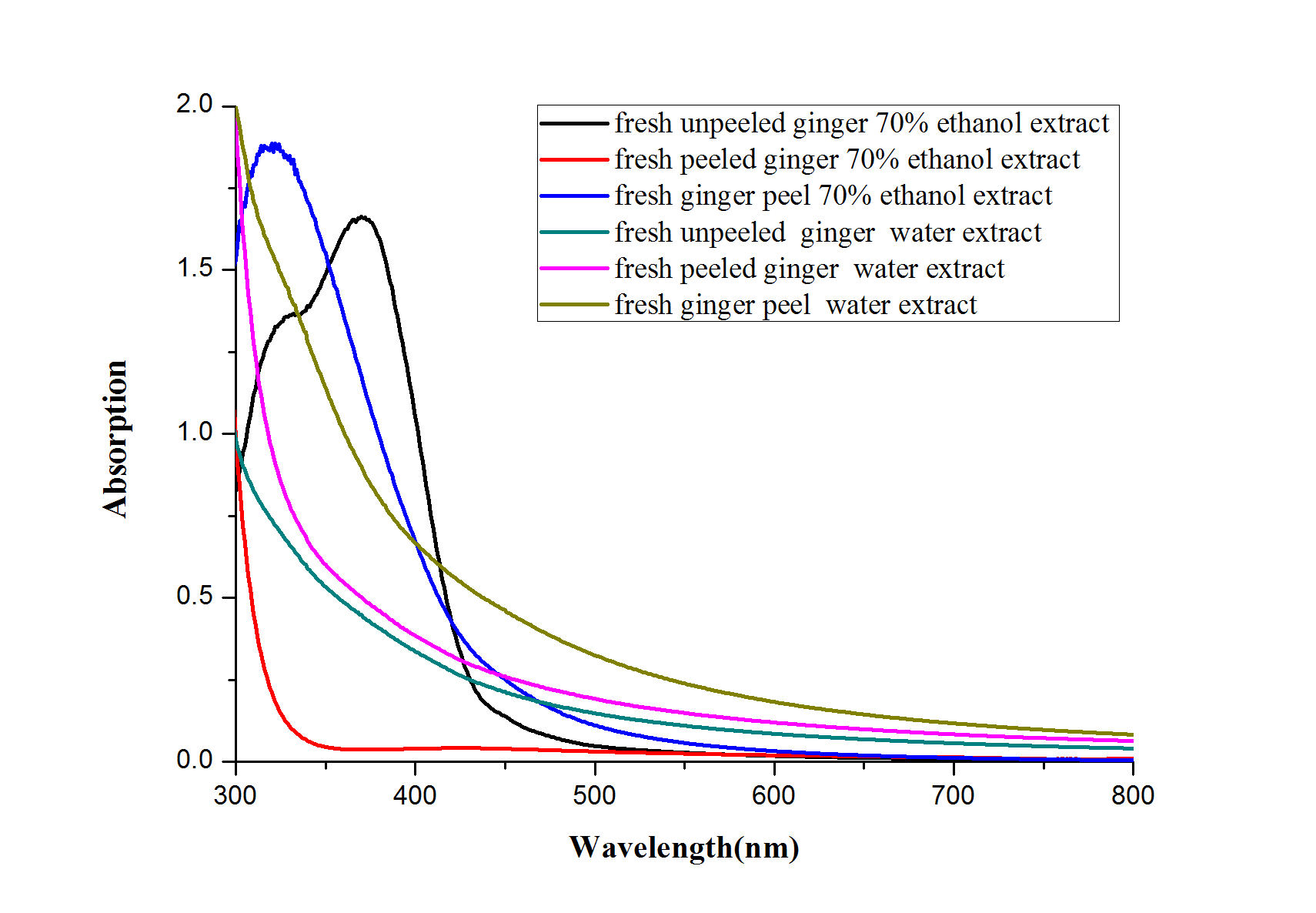

Supplement: S1 File — (ZIP) [file pone.0271408.s001.zip › supporting informations-PONE-D-22-11812/Fig 2-extract+AgNPs-UV/╨┬╧╩╠ß╚í╬∩.jpg]

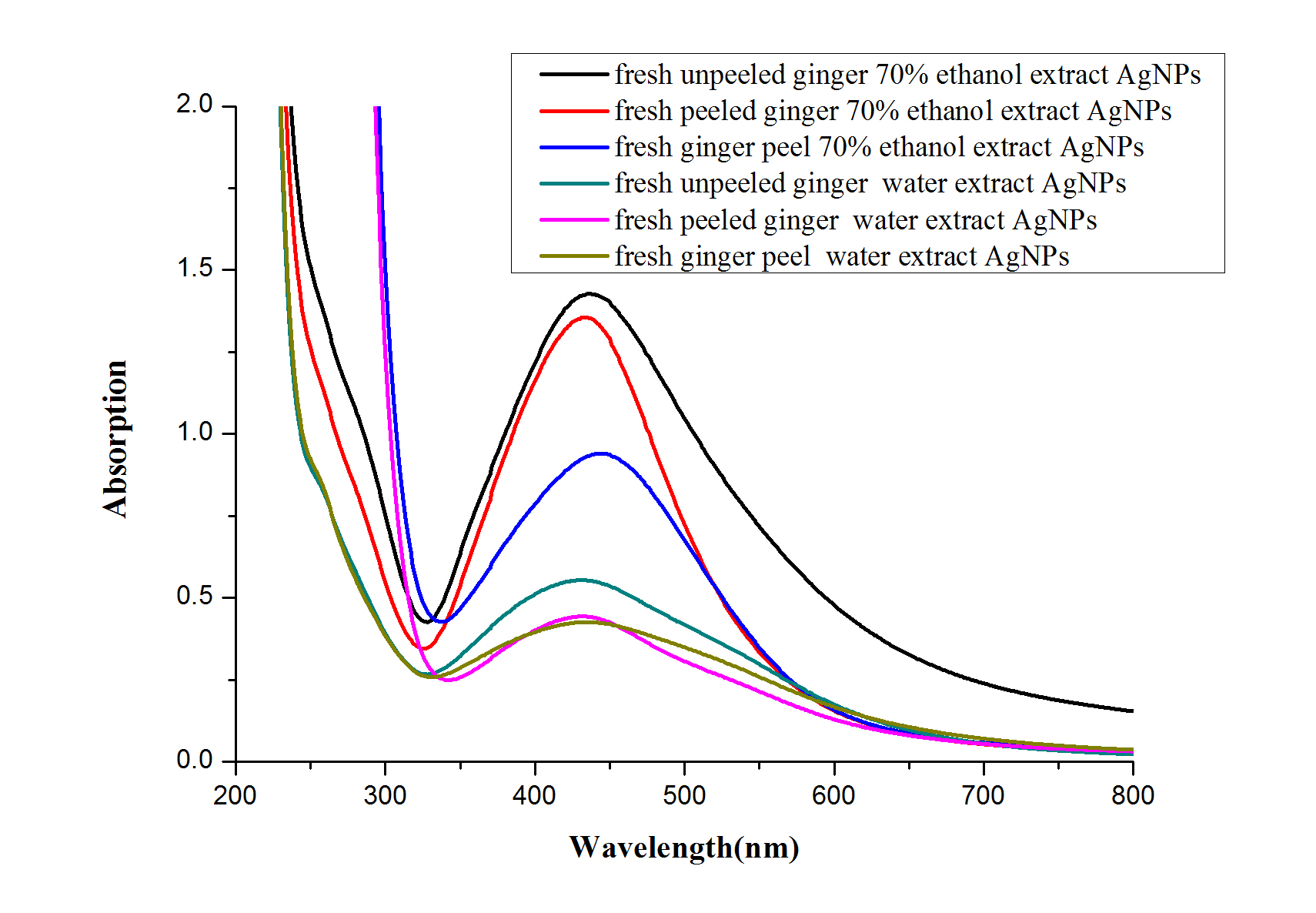

Supplement: S1 File — (ZIP) [file pone.0271408.s001.zip › supporting informations-PONE-D-22-11812/Fig 2-extract+AgNPs-UV/╨┬╧╩─╔├╫╥°.jpg]

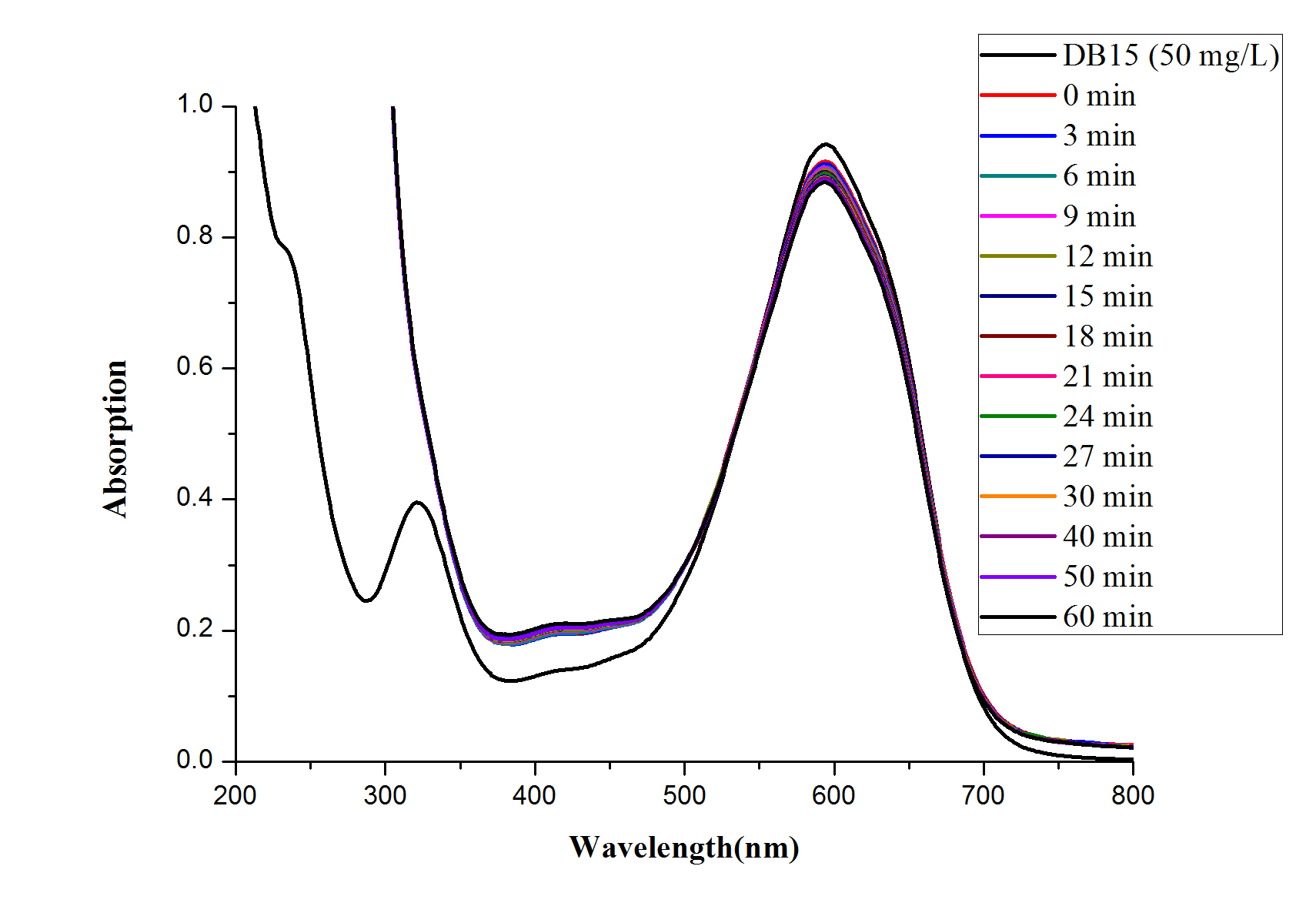

Supplement: S1 File — (ZIP) [file pone.0271408.s001.zip › supporting informations-PONE-D-22-11812/Figs5-12/DB15/DB15-CK.jpg]

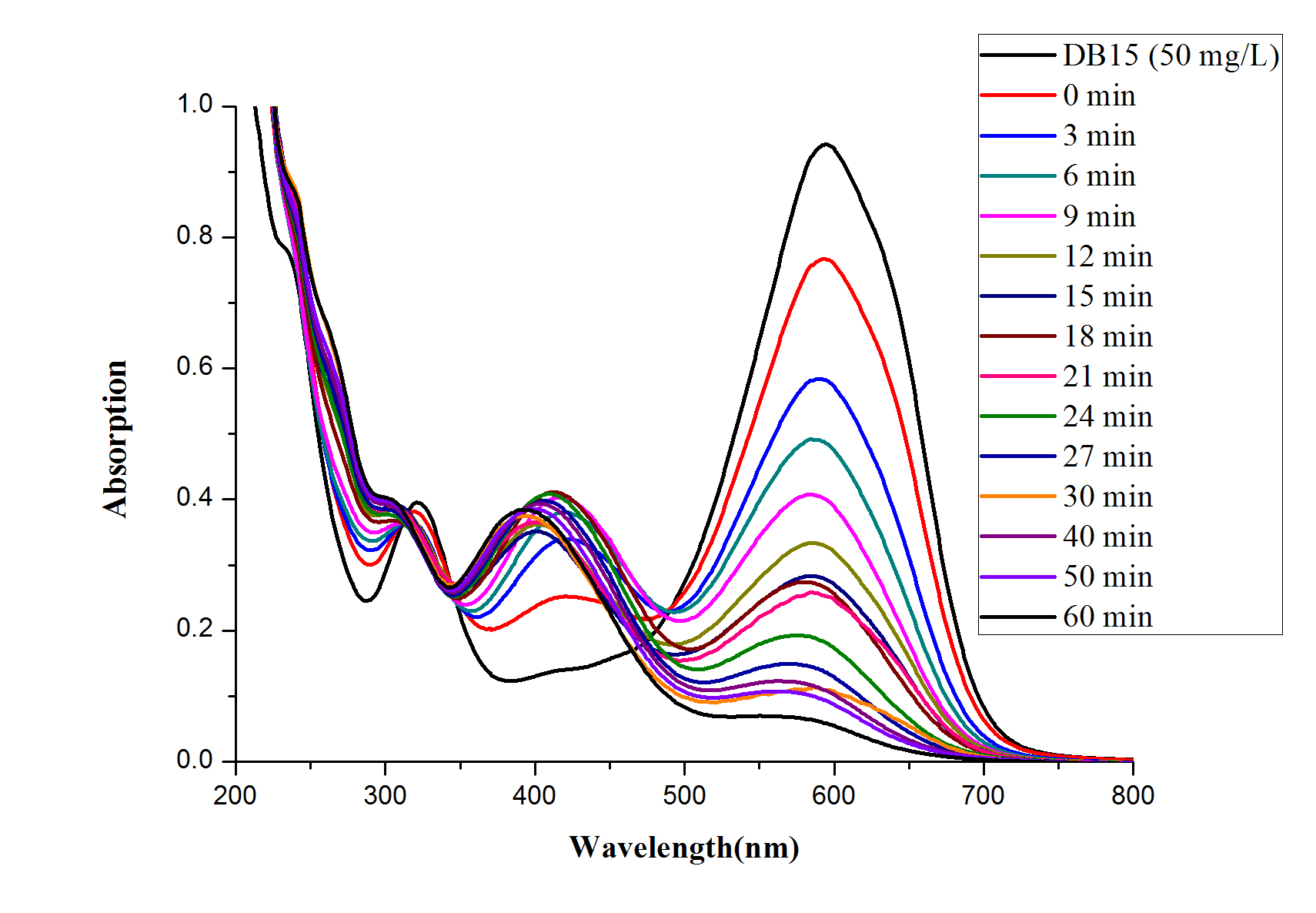

Supplement: S1 File — (ZIP) [file pone.0271408.s001.zip › supporting informations-PONE-D-22-11812/Figs5-12/DB15/dry AgNPs/6a 3mLDB15+0.1mL╕╔╓╞╬┤╚Ñ╞ñ╔·╜¬─╔├╫╥°ú¿╦«╠ß╬∩ú⌐-2.jpg]

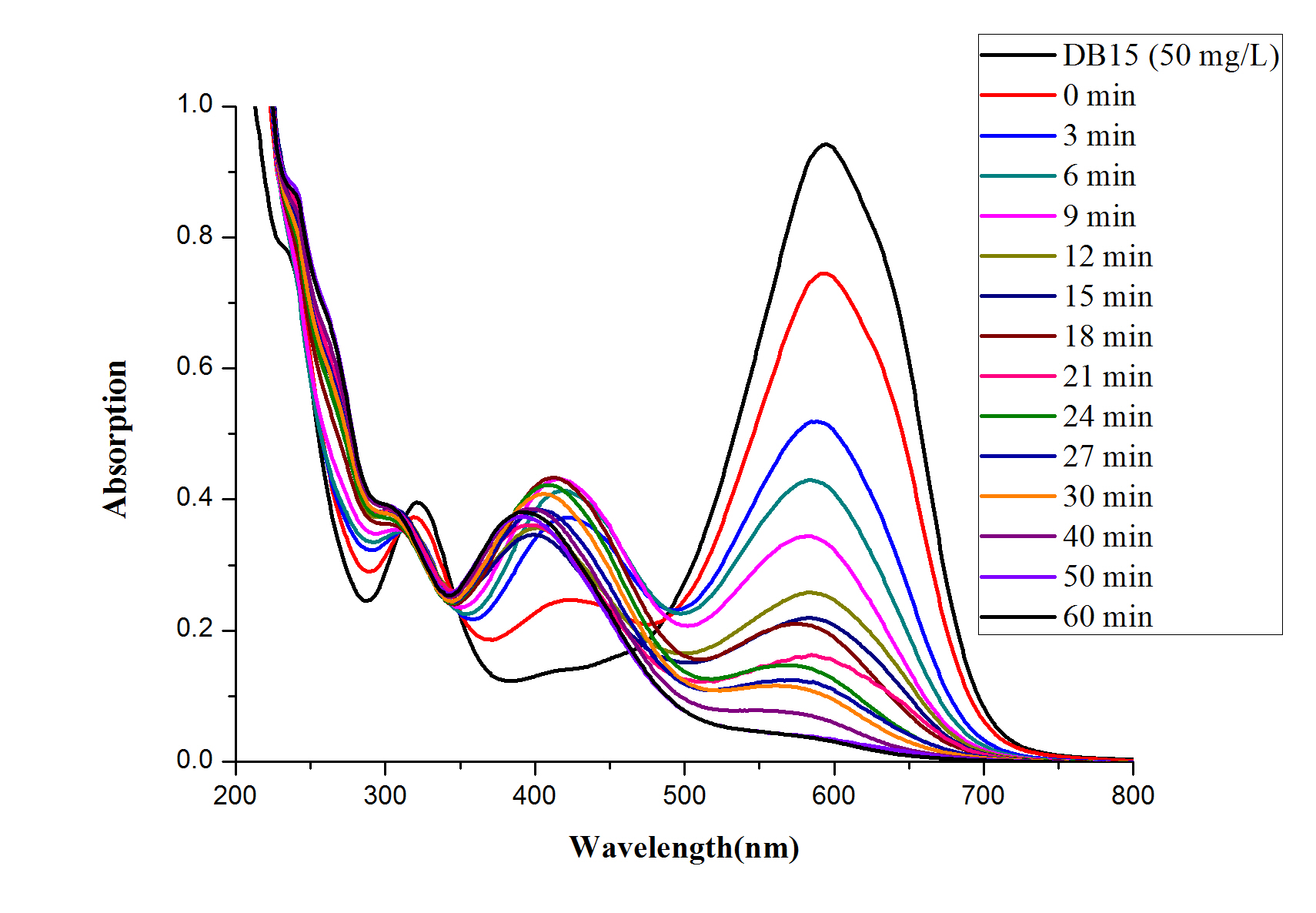

Supplement: S1 File — (ZIP) [file pone.0271408.s001.zip › supporting informations-PONE-D-22-11812/Figs5-12/DB15/dry AgNPs/6b 3mLDB15+0.1mL╕╔╓╞╚Ñ╞ñ╔·╜¬─╔├╫╥°ú¿╦«╠ß╬∩ú⌐-2.jpg]

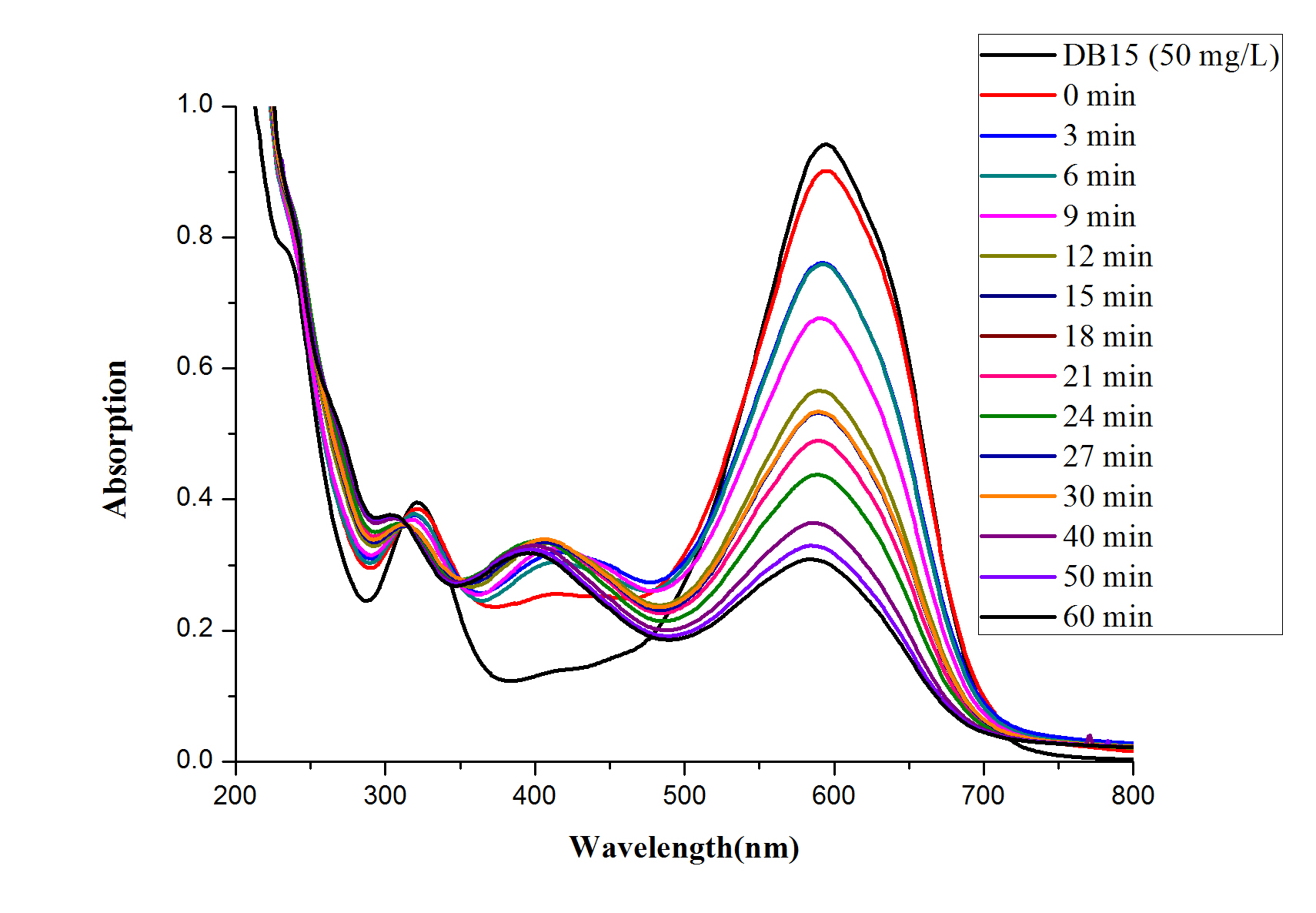

Supplement: S1 File — (ZIP) [file pone.0271408.s001.zip › supporting informations-PONE-D-22-11812/Figs5-12/DB15/dry AgNPs/6c 3mLDB15+0.1mL╕╔╓╞╔·╜¬╞ñ─╔├╫╥°ú¿╦«╠ß╬∩ú⌐-2.jpg]

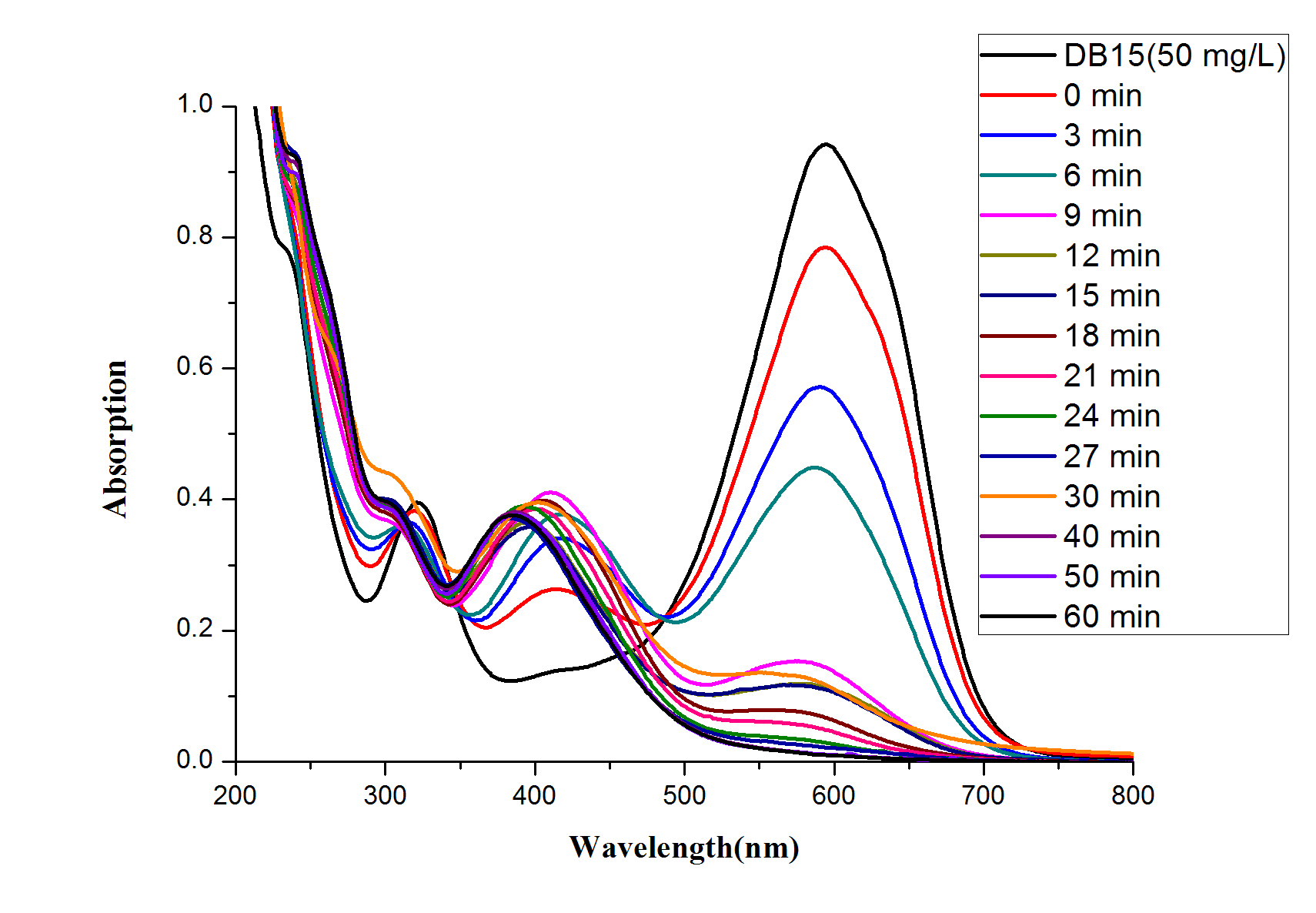

Supplement: S1 File — (ZIP) [file pone.0271408.s001.zip › supporting informations-PONE-D-22-11812/Figs5-12/DB15/dry AgNPs/6d 3mLDB15+0.1mL╕╔╓╞╬┤╚Ñ╞ñ╔·╜¬─╔├╫╥°ú¿┤╝╠ß╬∩ú⌐-2.jpg]

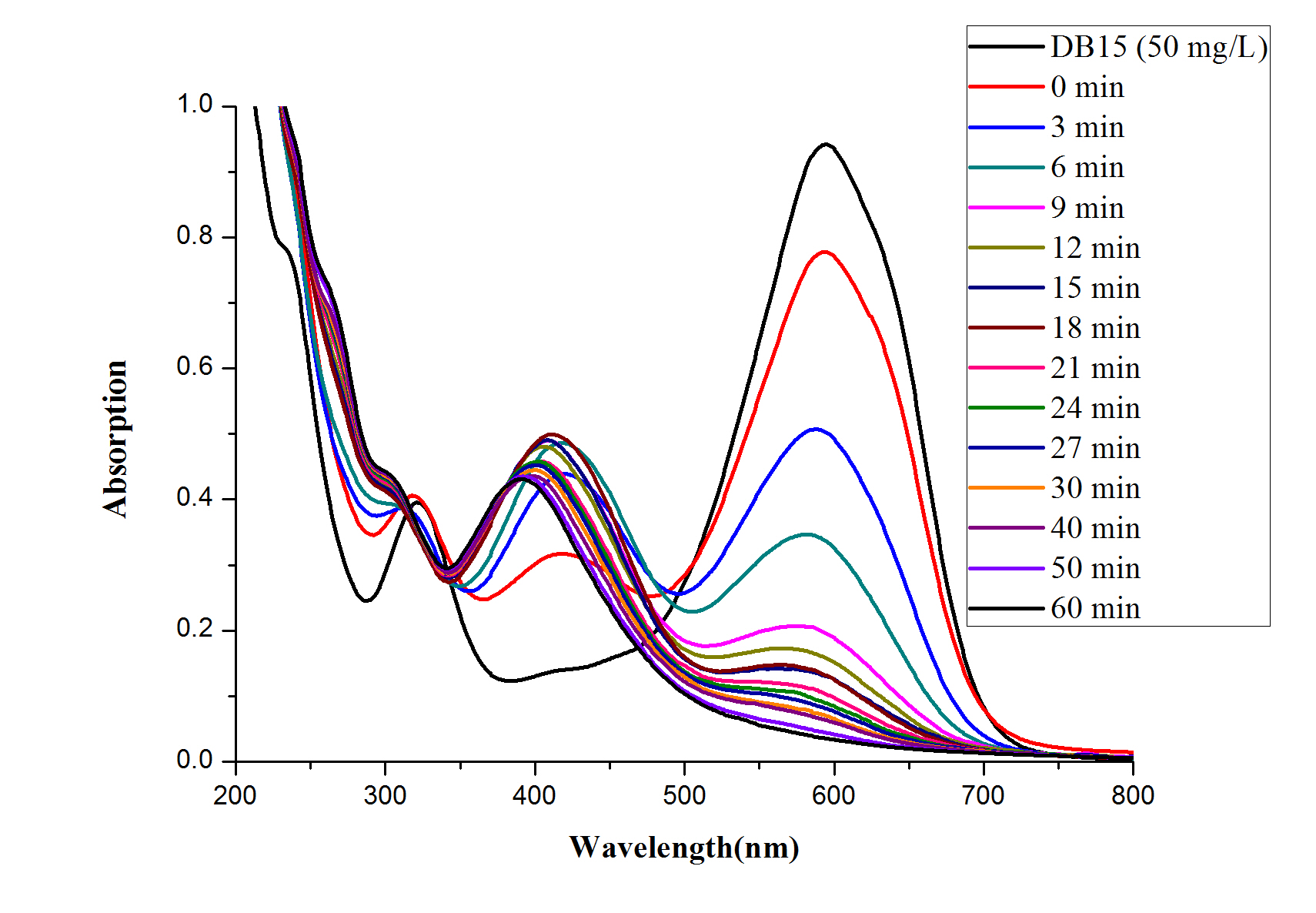

Supplement: S1 File — (ZIP) [file pone.0271408.s001.zip › supporting informations-PONE-D-22-11812/Figs5-12/DB15/dry AgNPs/6e 3mLDB15+0.1mL╕╔╓╞╚Ñ╞ñ╔·╜¬─╔├╫╥°ú¿┤╝╠ß╬∩ú⌐-2.jpg]

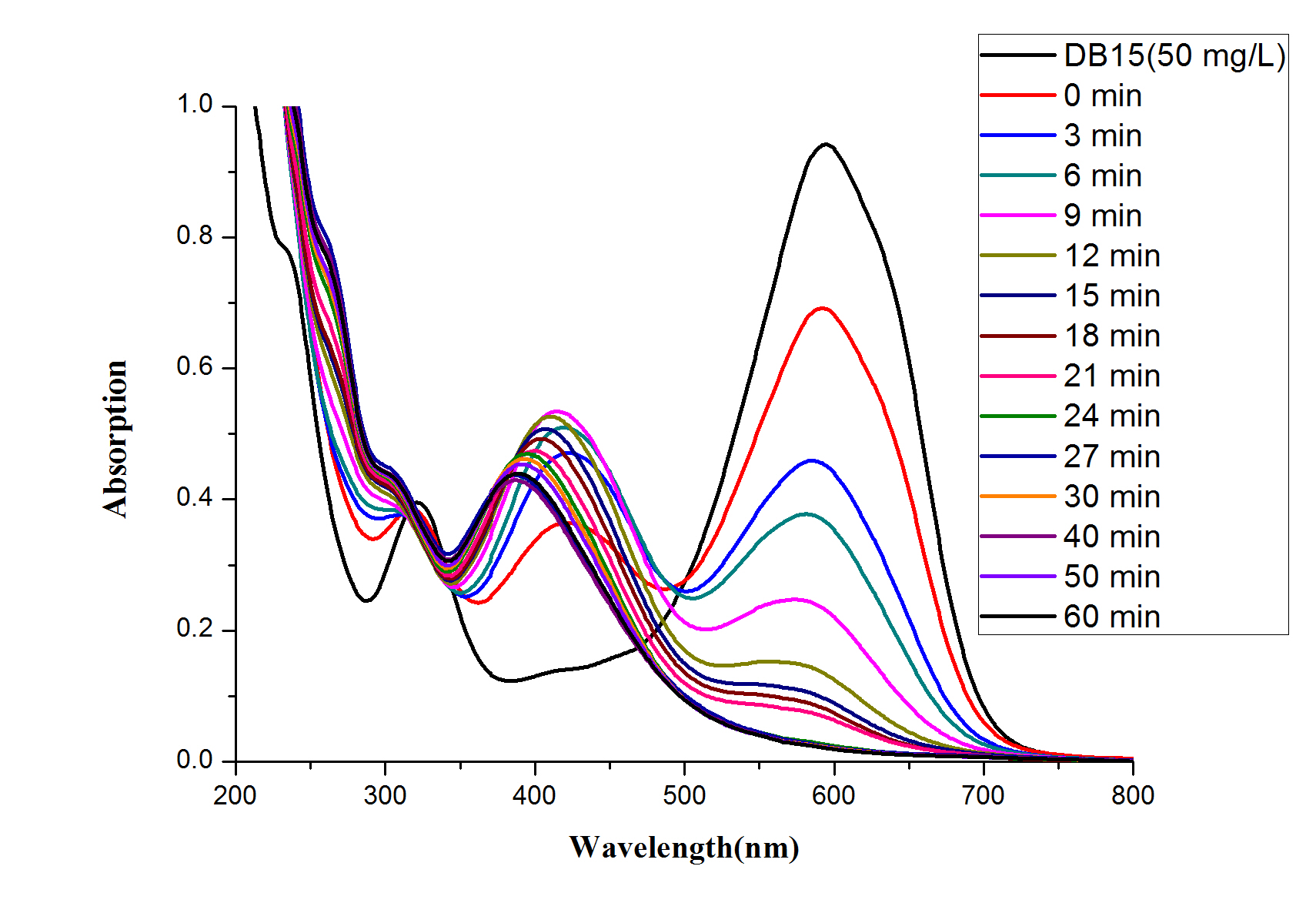

Supplement: S1 File — (ZIP) [file pone.0271408.s001.zip › supporting informations-PONE-D-22-11812/Figs5-12/DB15/dry AgNPs/6f 3mLDB15+0.1mL╕╔╓╞╔·╜¬╞ñ─╔├╫╥°ú¿┤╝╠ß╬∩ú⌐-2.jpg]

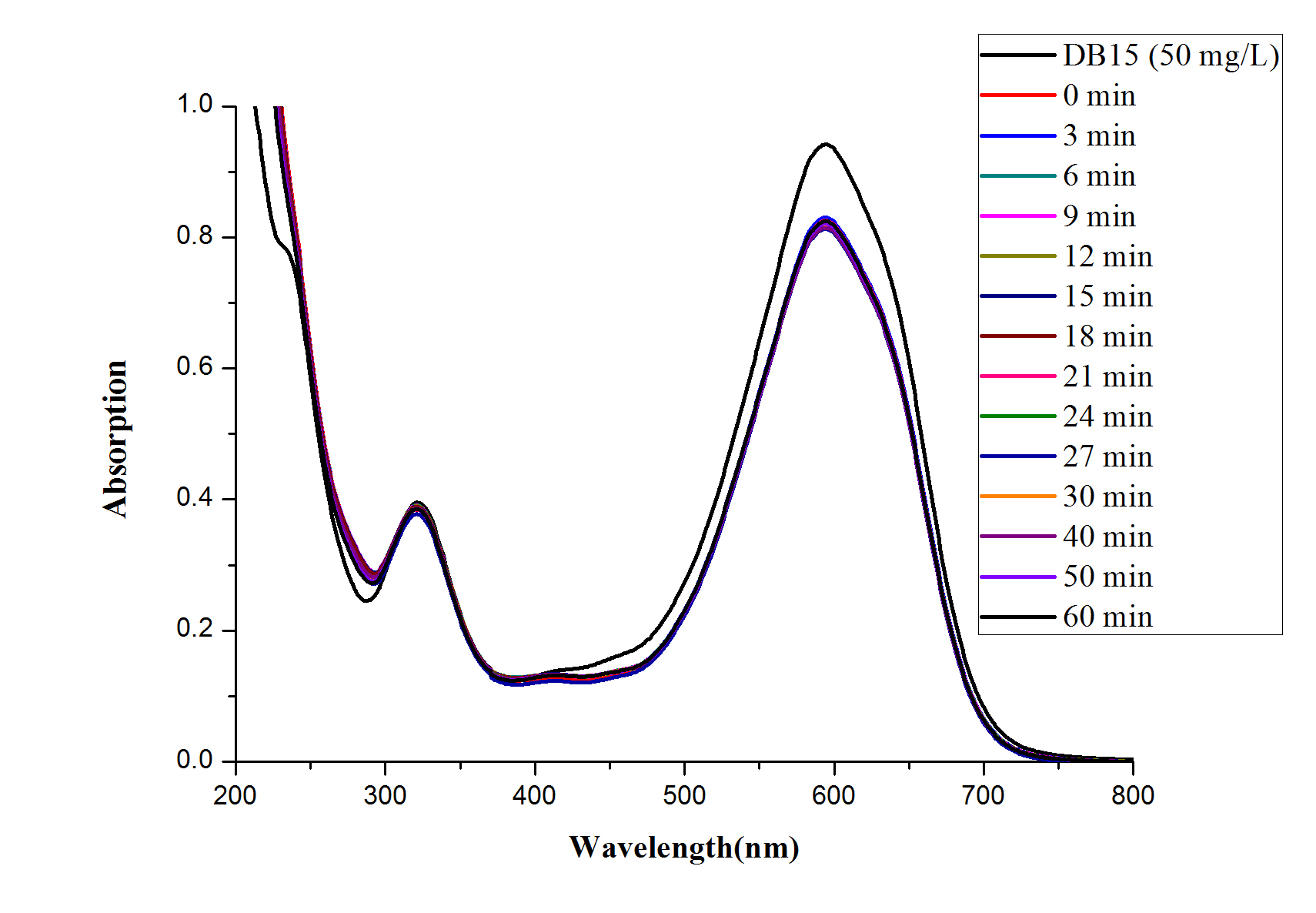

Supplement: S1 File — (ZIP) [file pone.0271408.s001.zip › supporting informations-PONE-D-22-11812/Figs5-12/DB15/dry extract/8a 3mLDB15+0.1mL╕╔╓╞╬┤╚Ñ╞ñ╔·╜¬ú¿╦«╠ß╬∩ú⌐-.jpg]

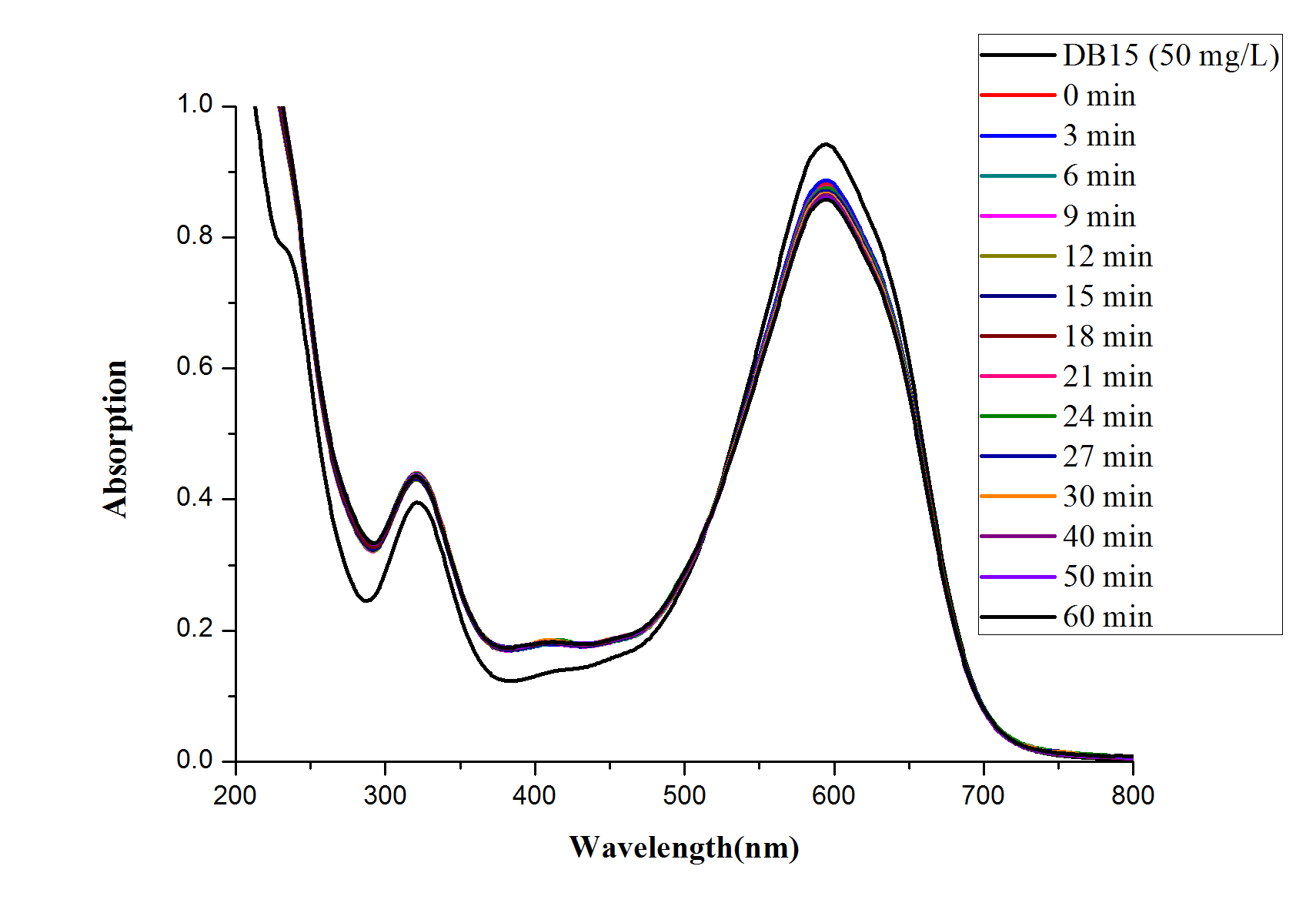

Supplement: S1 File — (ZIP) [file pone.0271408.s001.zip › supporting informations-PONE-D-22-11812/Figs5-12/DB15/dry extract/8b 3mLDB15+0.1mL╕╔╓╞╚Ñ╞ñ╔·╜¬ú¿╦«╠ß╬∩ú⌐-.jpg]

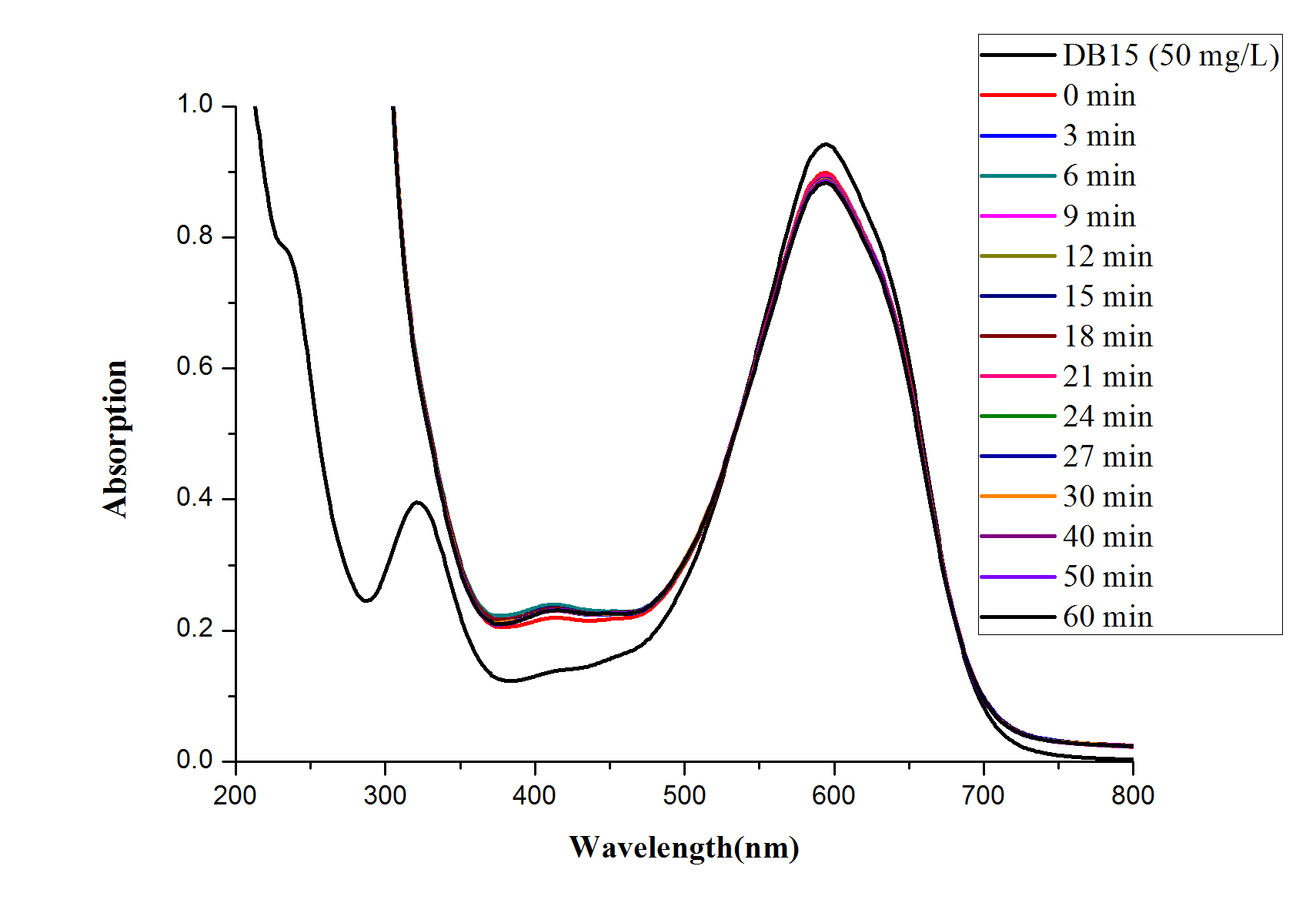

Supplement: S1 File — (ZIP) [file pone.0271408.s001.zip › supporting informations-PONE-D-22-11812/Figs5-12/DB15/dry extract/8c 3mLDB15+0.1mL╕╔╓╞╔·╜¬╞ñú¿╦«╠ß╬∩ú⌐-.jpg]

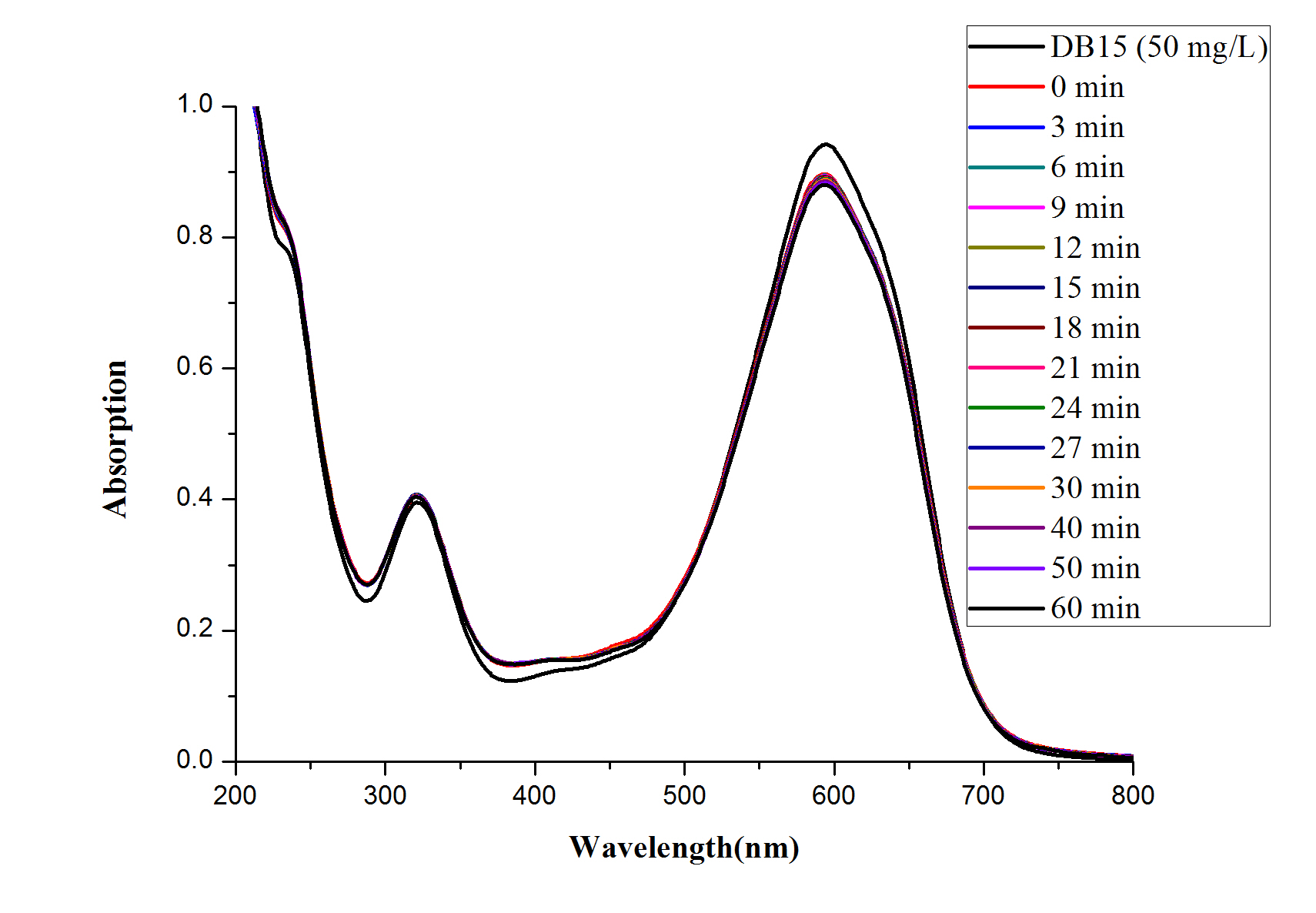

Supplement: S1 File — (ZIP) [file pone.0271408.s001.zip › supporting informations-PONE-D-22-11812/Figs5-12/DB15/dry extract/8d 3mLDB15+0.1mL╕╔╓╞╬┤╚Ñ╞ñ╔·╜¬┤╝╠ß╬∩.jpg]

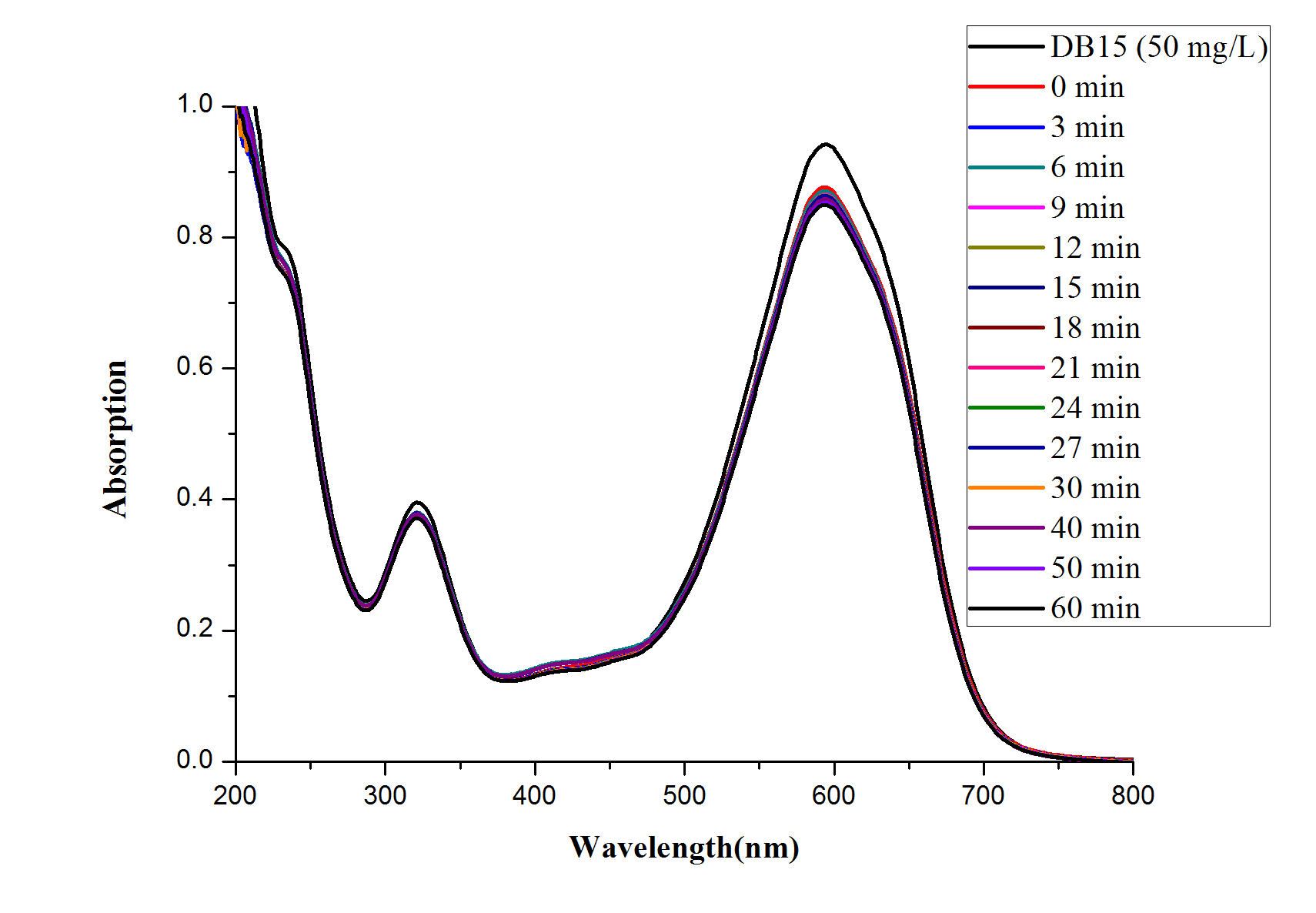

Supplement: S1 File — (ZIP) [file pone.0271408.s001.zip › supporting informations-PONE-D-22-11812/Figs5-12/DB15/dry extract/8e 3mLDB15+0.1mL╕╔╓╞╚Ñ╞ñ╔·╜¬┤╝╠ß╬∩.jpg]

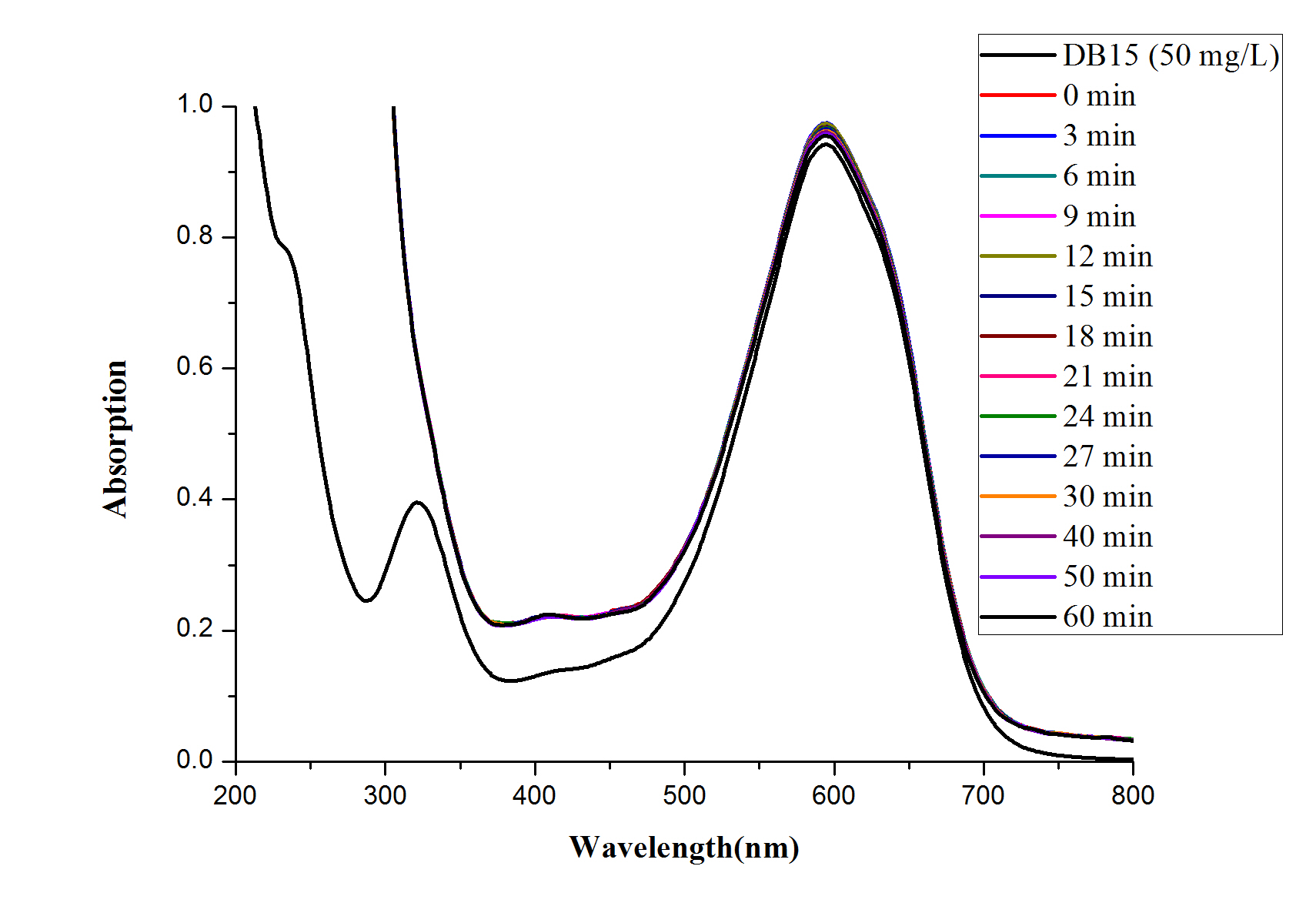

Supplement: S1 File — (ZIP) [file pone.0271408.s001.zip › supporting informations-PONE-D-22-11812/Figs5-12/DB15/dry extract/8f 3mLDB15+0.1mL╕╔╓╞╔·╜¬╞ñú¿┤╝╠ß╬∩ú⌐-.jpg]

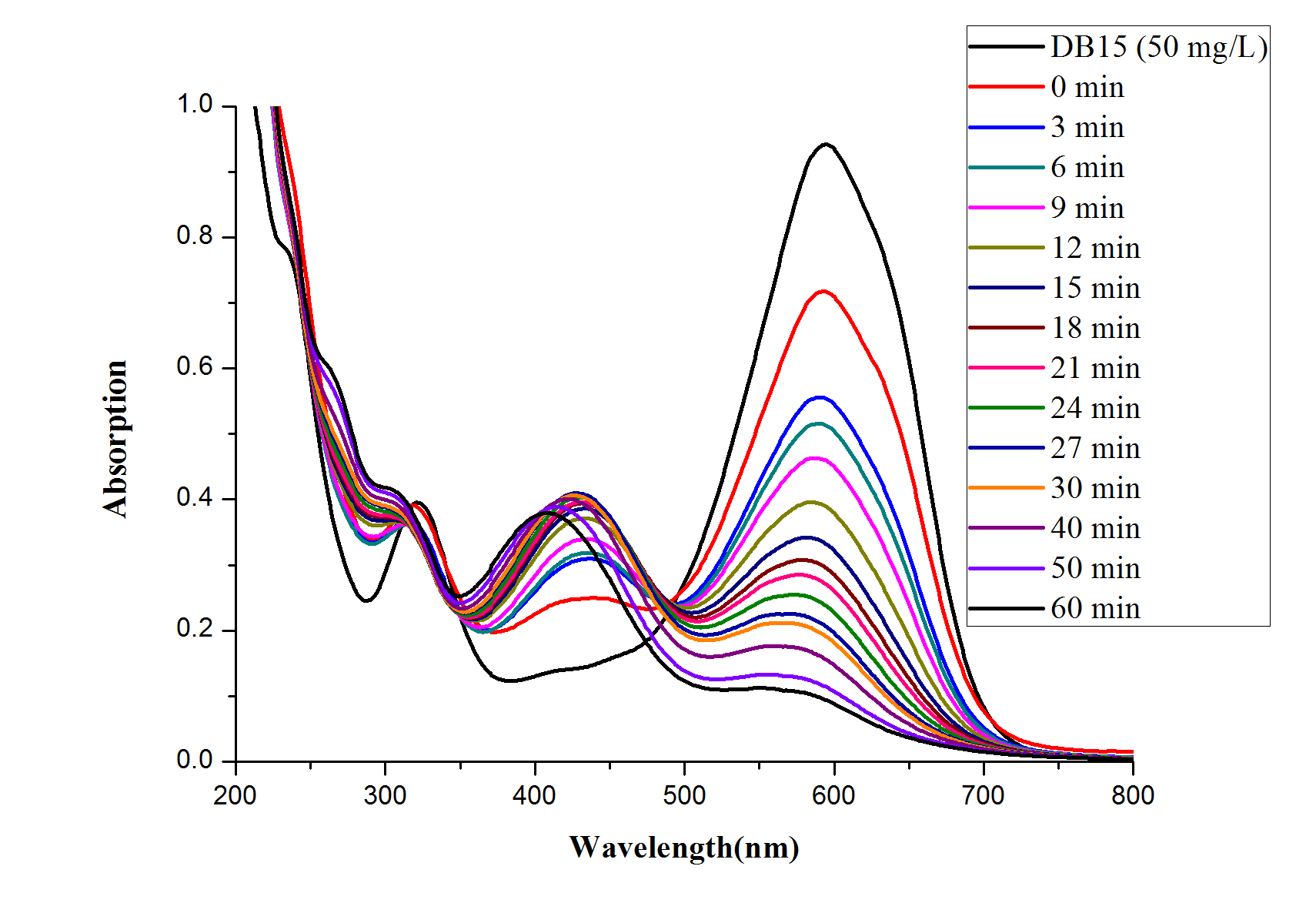

Supplement: S1 File — (ZIP) [file pone.0271408.s001.zip › supporting informations-PONE-D-22-11812/Figs5-12/DB15/fresh AgNPs/5a 3mLDB15+0.1mL╨┬╧╩╬┤╚Ñ╞ñ╔·╜¬─╔├╫╥°ú¿╦«╠ß╬∩ú⌐-2.jpg]

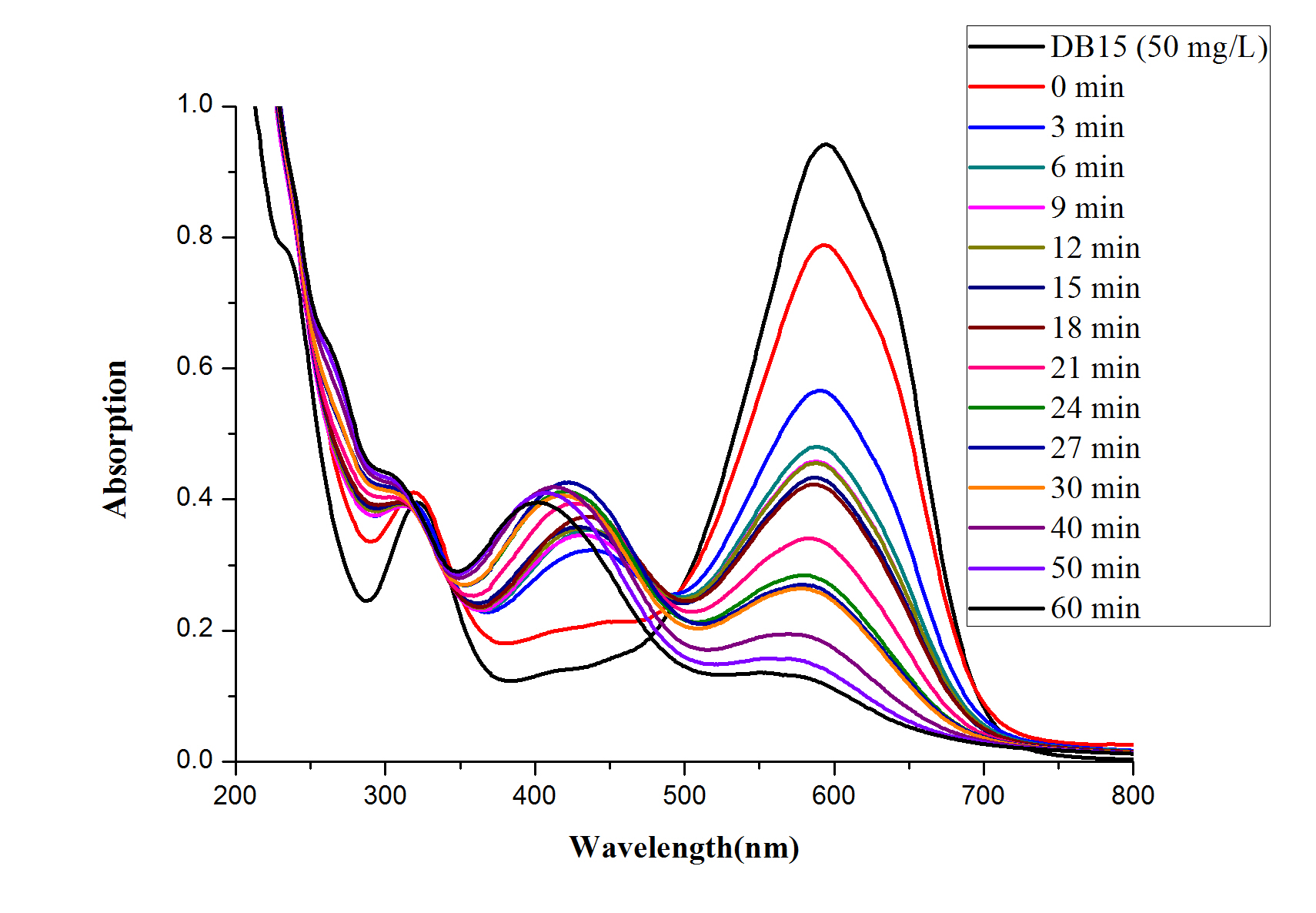

Supplement: S1 File — (ZIP) [file pone.0271408.s001.zip › supporting informations-PONE-D-22-11812/Figs5-12/DB15/fresh AgNPs/5b 3mLDB15+0.1mL╨┬╧╩╚Ñ╞ñ╔·╜¬─╔├╫╥°ú¿╦«ú⌐-2.jpg]

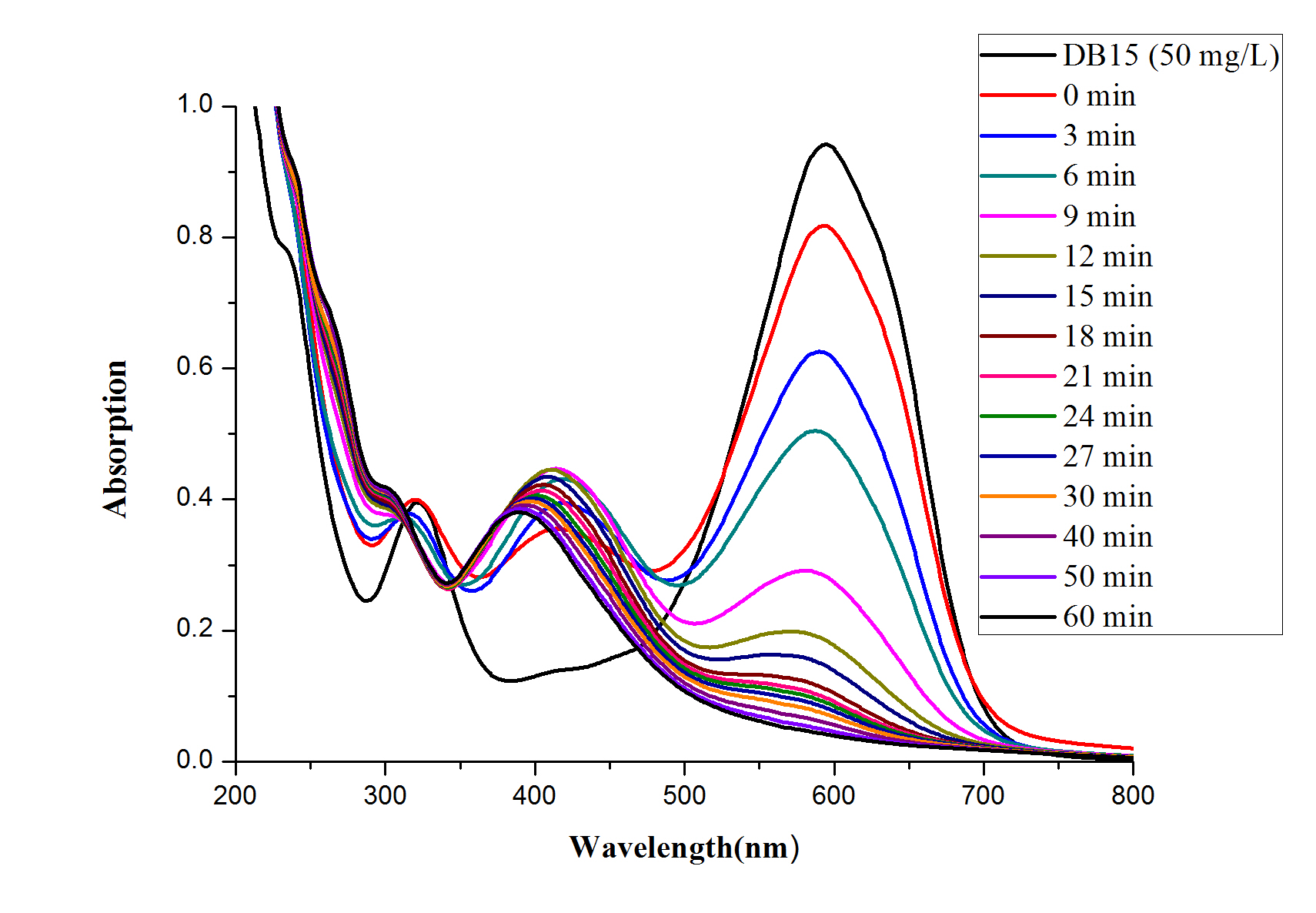

Supplement: S1 File — (ZIP) [file pone.0271408.s001.zip › supporting informations-PONE-D-22-11812/Figs5-12/DB15/fresh AgNPs/5c 3mLDB15+0.1mL╨┬╧╩╔·╜¬╞ñ─╔├╫╥°ú¿╦«ú⌐-2.jpg]

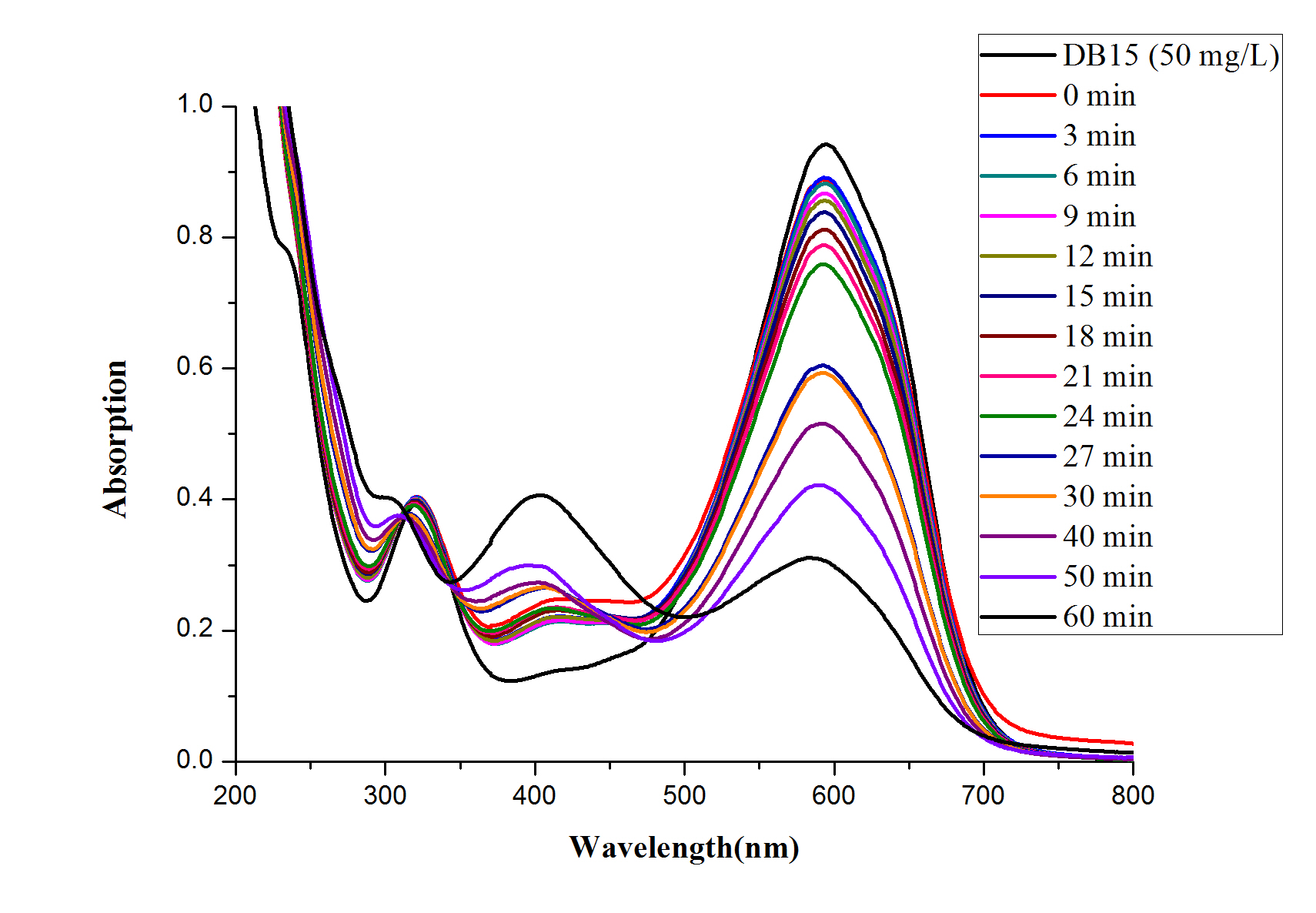

Supplement: S1 File — (ZIP) [file pone.0271408.s001.zip › supporting informations-PONE-D-22-11812/Figs5-12/DB15/fresh AgNPs/5d 3mLDB15+0.1mL╨┬╧╩╬┤╚Ñ╞ñ╔·╜¬─╔├╫╥°ú¿┤╝ú⌐-2.jpg]

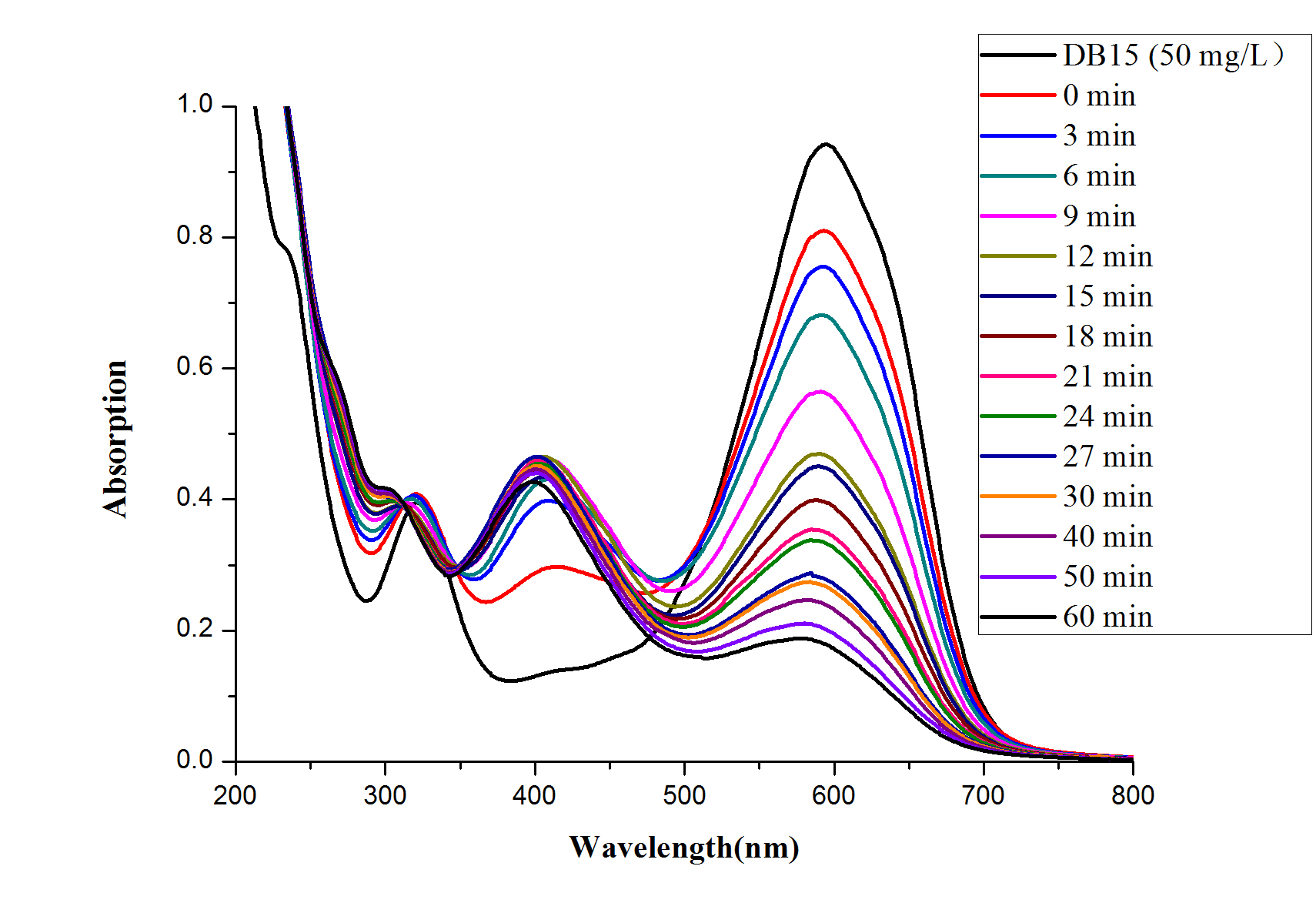

Supplement: S1 File — (ZIP) [file pone.0271408.s001.zip › supporting informations-PONE-D-22-11812/Figs5-12/DB15/fresh AgNPs/5e 3mLDB15+0.1mL╨┬╧╩╚Ñ╞ñ╔·╜¬─╔├╫╥°ú¿┤╝ú⌐-2.jpg]

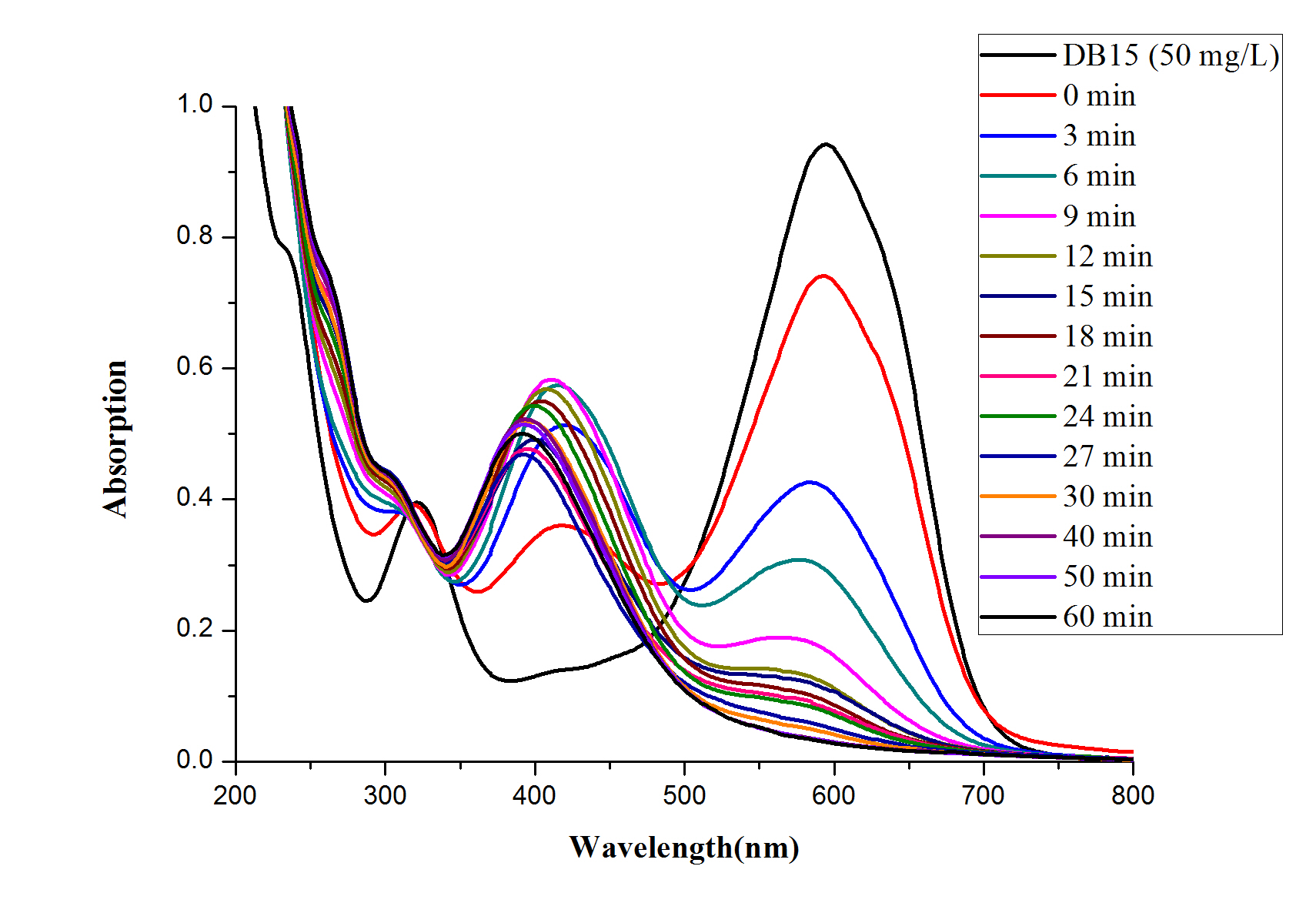

Supplement: S1 File — (ZIP) [file pone.0271408.s001.zip › supporting informations-PONE-D-22-11812/Figs5-12/DB15/fresh AgNPs/5f 3mLDB15+0.1mL╨┬╧╩╔·╜¬╞ñ─╔├╫╥°ú¿┤╝ú⌐-2.jpg]

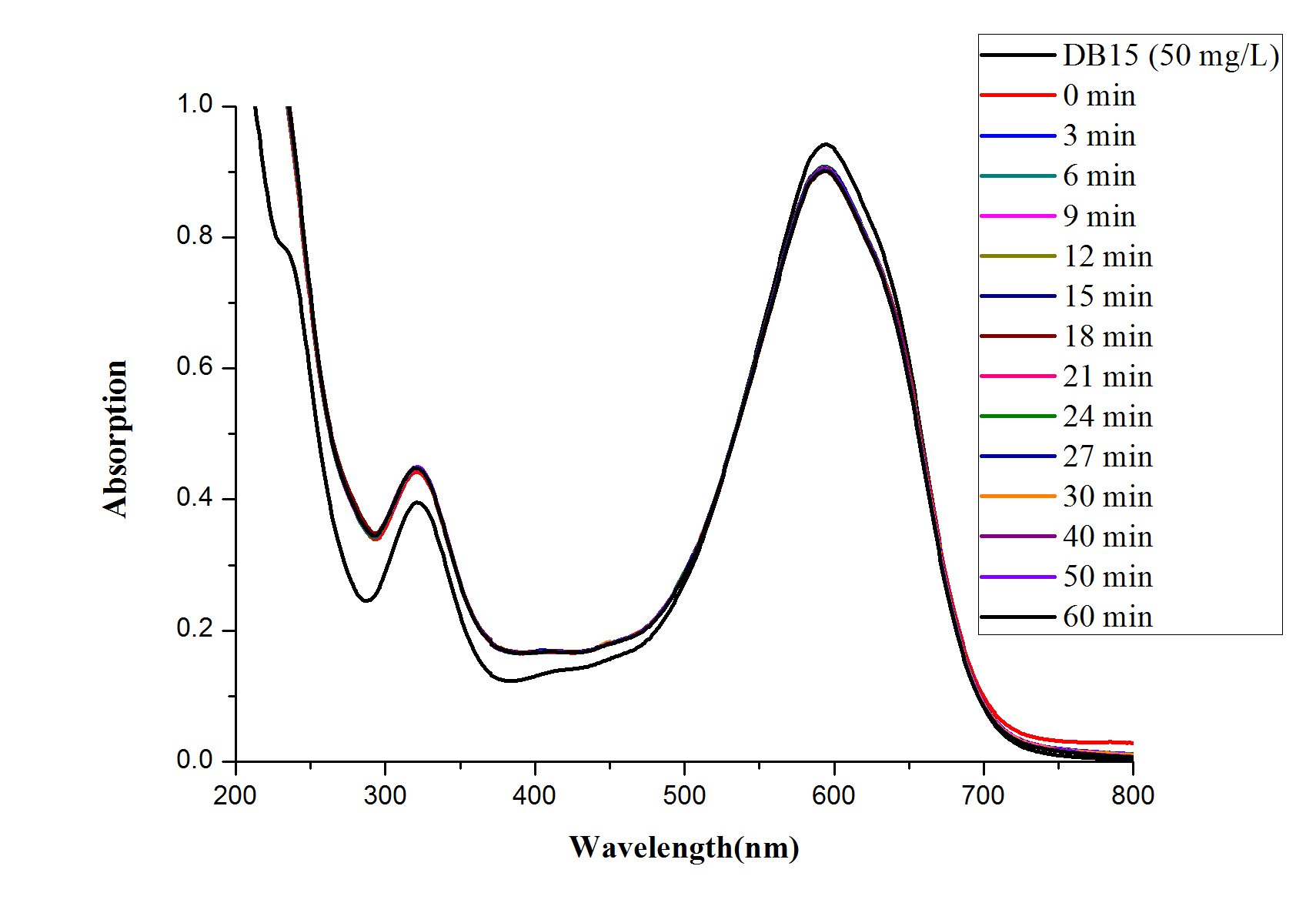

Supplement: S1 File — (ZIP) [file pone.0271408.s001.zip › supporting informations-PONE-D-22-11812/Figs5-12/DB15/fresh extract/7a 3mLDB15+0.1mL╨┬╧╩╬┤╚Ñ╞ñ╔·╜¬╦«╠ß╚í╬∩-.jpg]

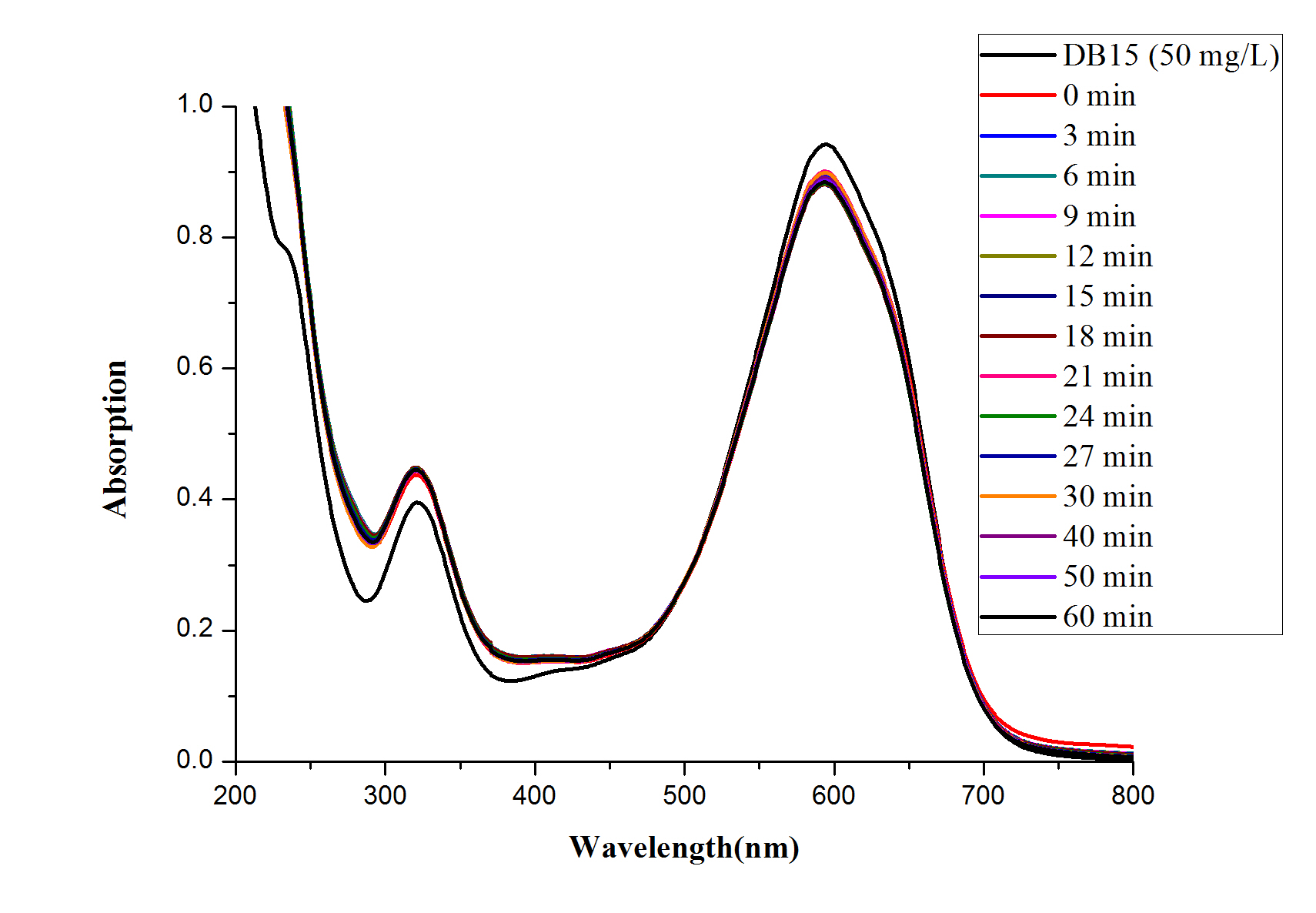

Supplement: S1 File — (ZIP) [file pone.0271408.s001.zip › supporting informations-PONE-D-22-11812/Figs5-12/DB15/fresh extract/7b 3mLDB15+0.1mL╨┬╧╩╚Ñ╞ñ╔·╜¬╦«╠ß╚í╬∩-.jpg]

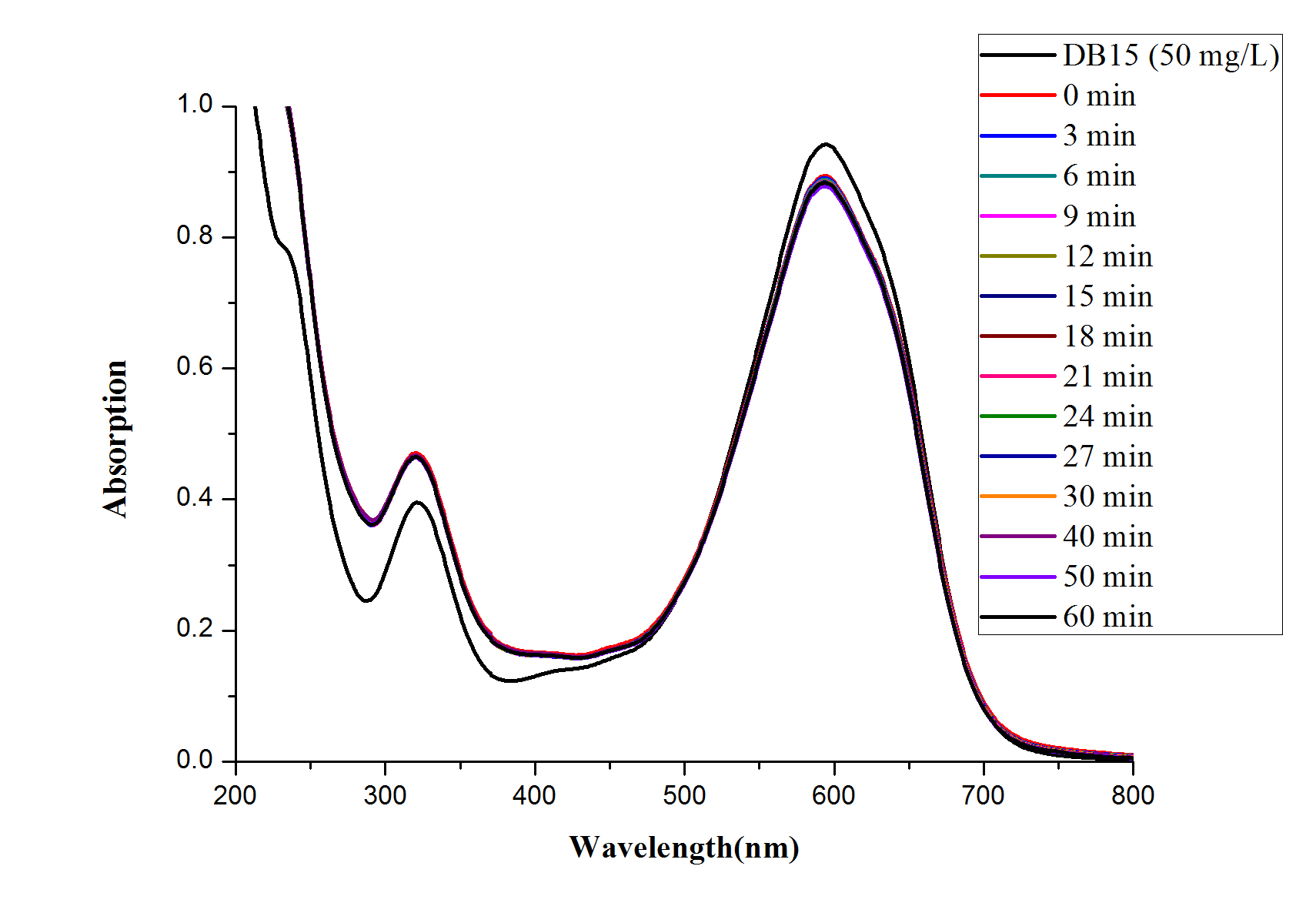

Supplement: S1 File — (ZIP) [file pone.0271408.s001.zip › supporting informations-PONE-D-22-11812/Figs5-12/DB15/fresh extract/7c 3mLDB15+0.1mL╨┬╧╩╔·╜¬╞ñ╦«╠ß╚í╬∩-.jpg]

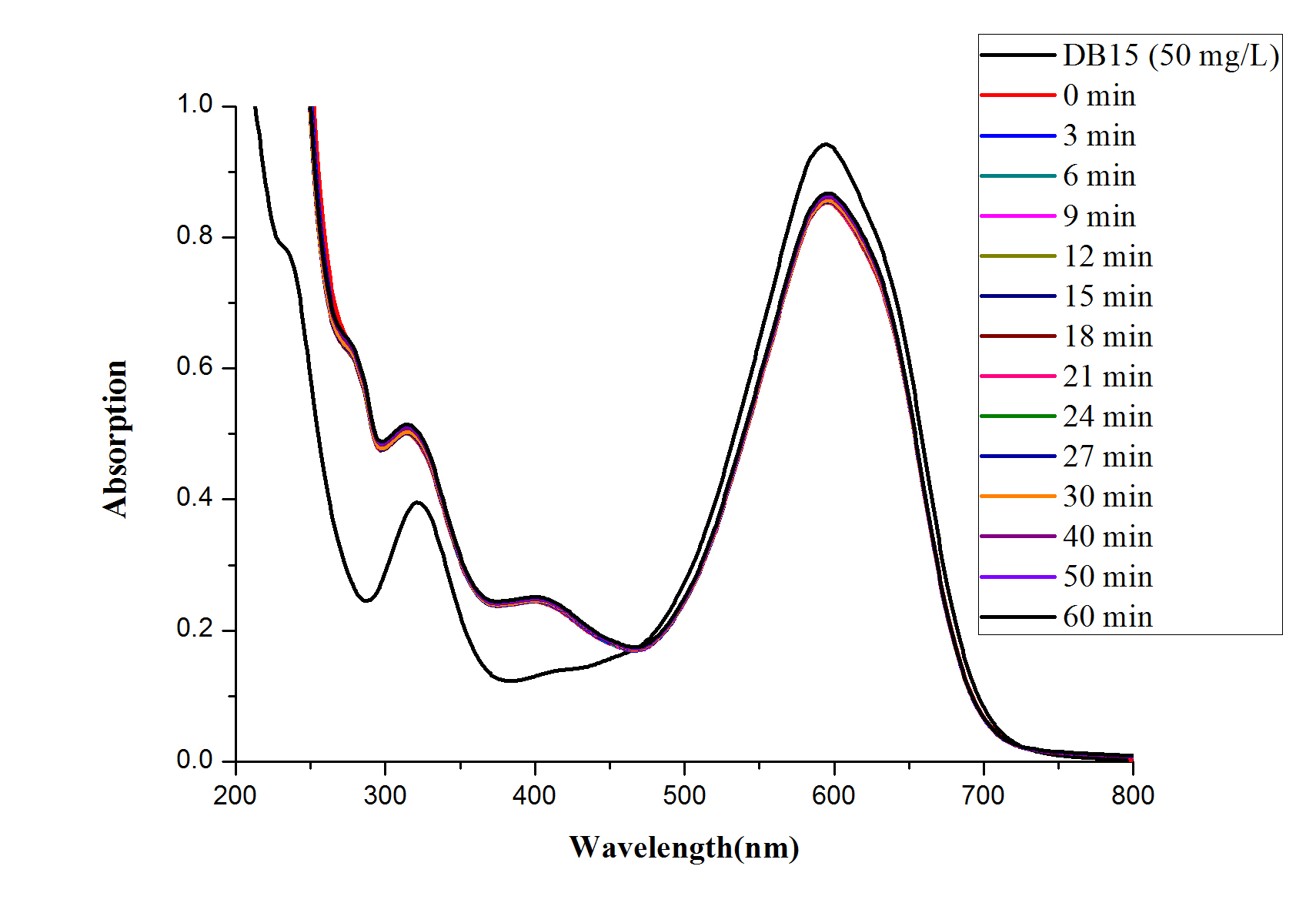

Supplement: S1 File — (ZIP) [file pone.0271408.s001.zip › supporting informations-PONE-D-22-11812/Figs5-12/DB15/fresh extract/7d 3mLDB15+0.1mL╨┬╧╩╬┤╚Ñ╞ñ╔·╜¬┤╝╠ß╚í╬∩-.jpg]

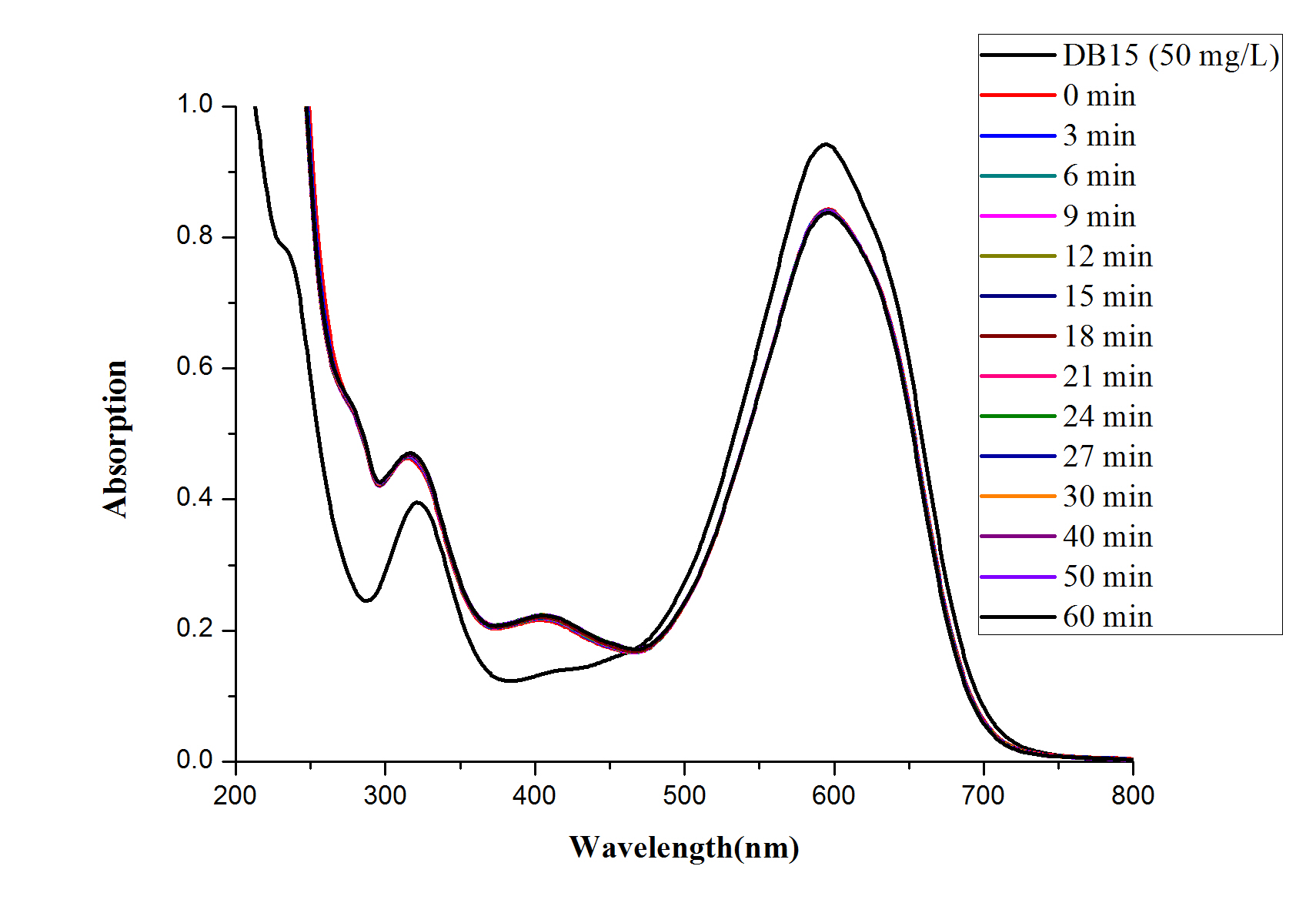

Supplement: S1 File — (ZIP) [file pone.0271408.s001.zip › supporting informations-PONE-D-22-11812/Figs5-12/DB15/fresh extract/7e 3mLDB15+0.1mL╨┬╧╩╚Ñ╞ñ╔·╜¬┤╝╠ß╚í╬∩-.jpg]

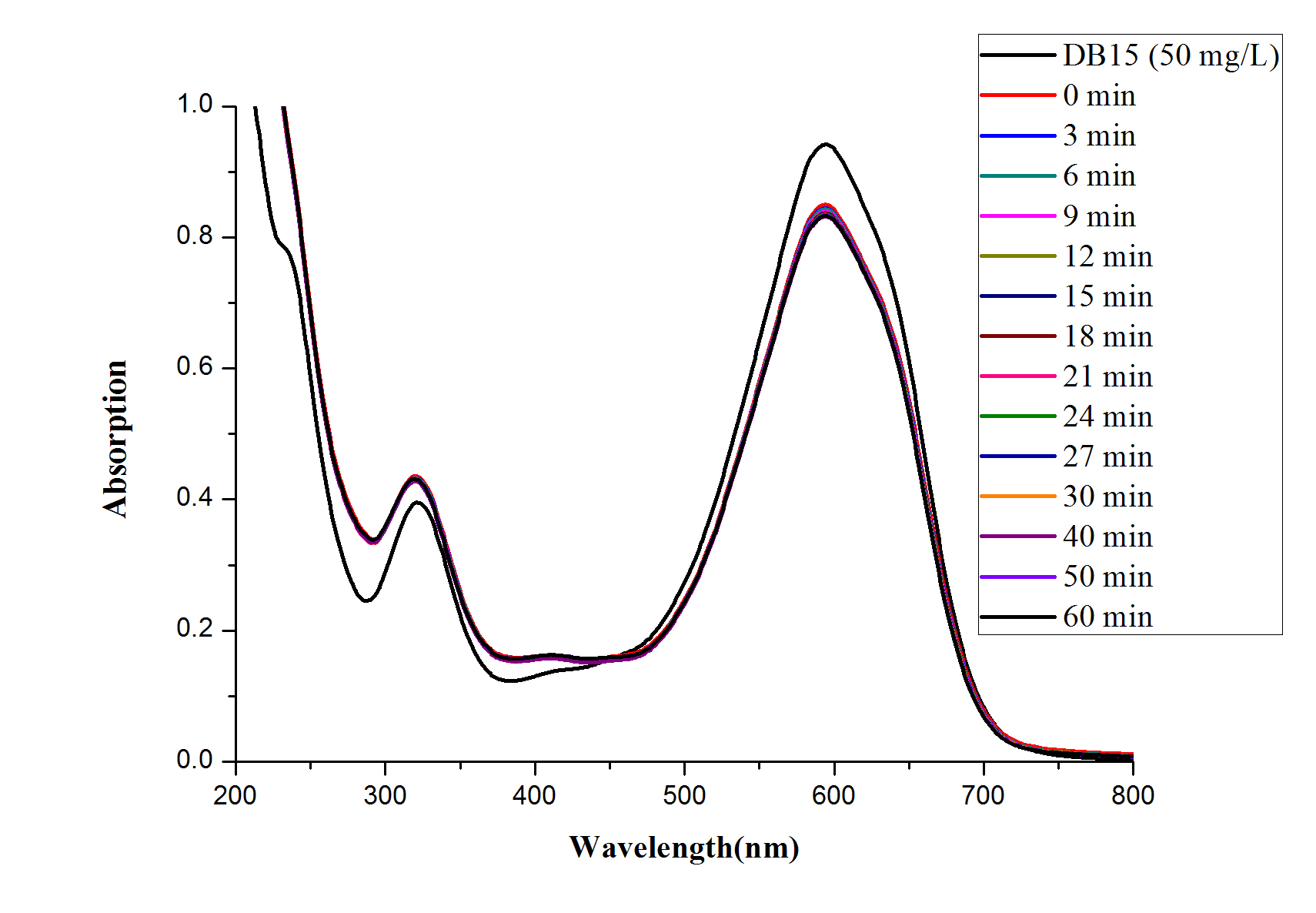

Supplement: S1 File — (ZIP) [file pone.0271408.s001.zip › supporting informations-PONE-D-22-11812/Figs5-12/DB15/fresh extract/7f 3mLDB15+0.1mL╨┬╧╩╔·╜¬╞ñ┤╝╠ß╚í╬∩-.jpg]

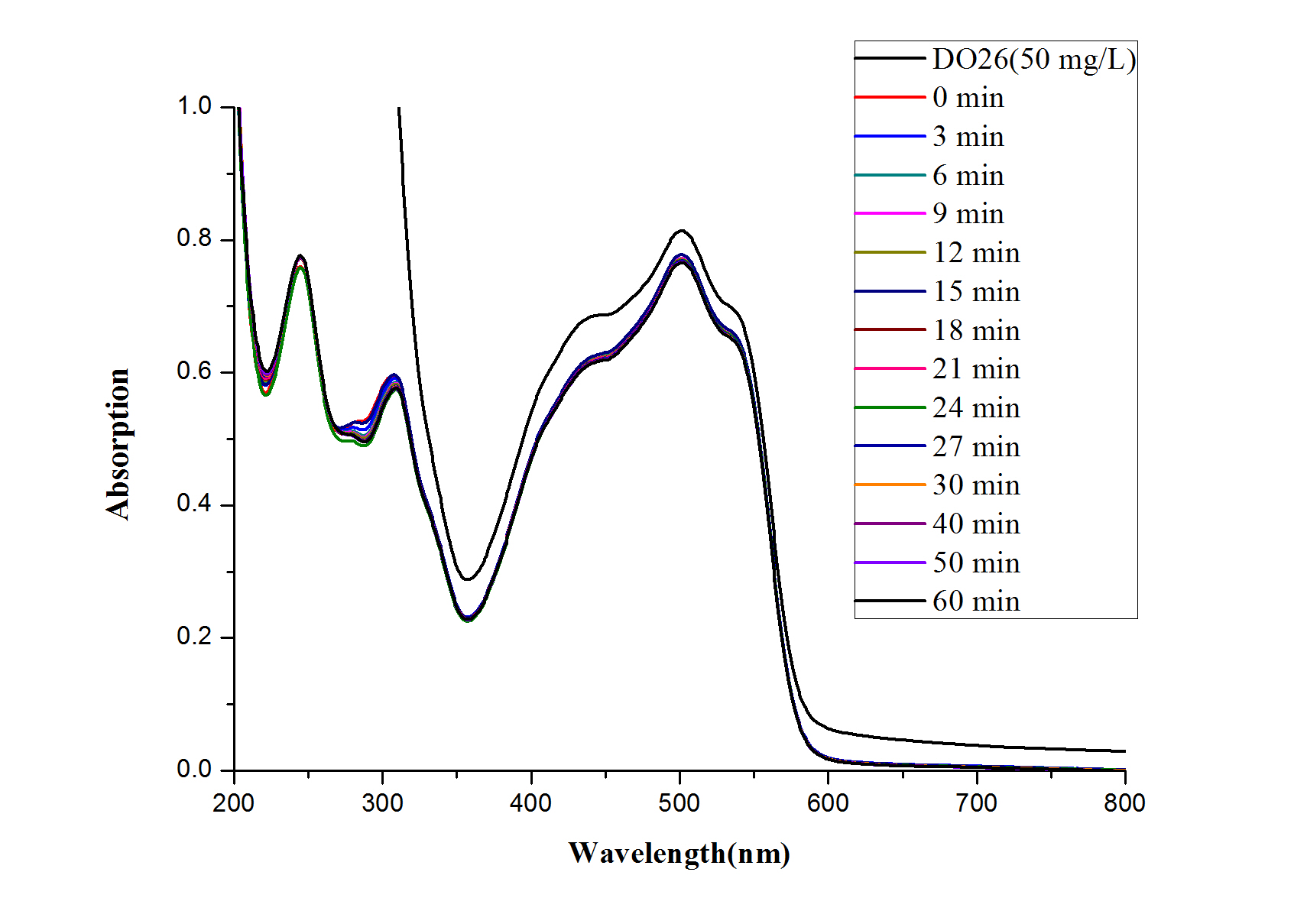

Supplement: S1 File — (ZIP) [file pone.0271408.s001.zip › supporting informations-PONE-D-22-11812/Figs5-12/DO26/DO26+╦«-CK.jpg]

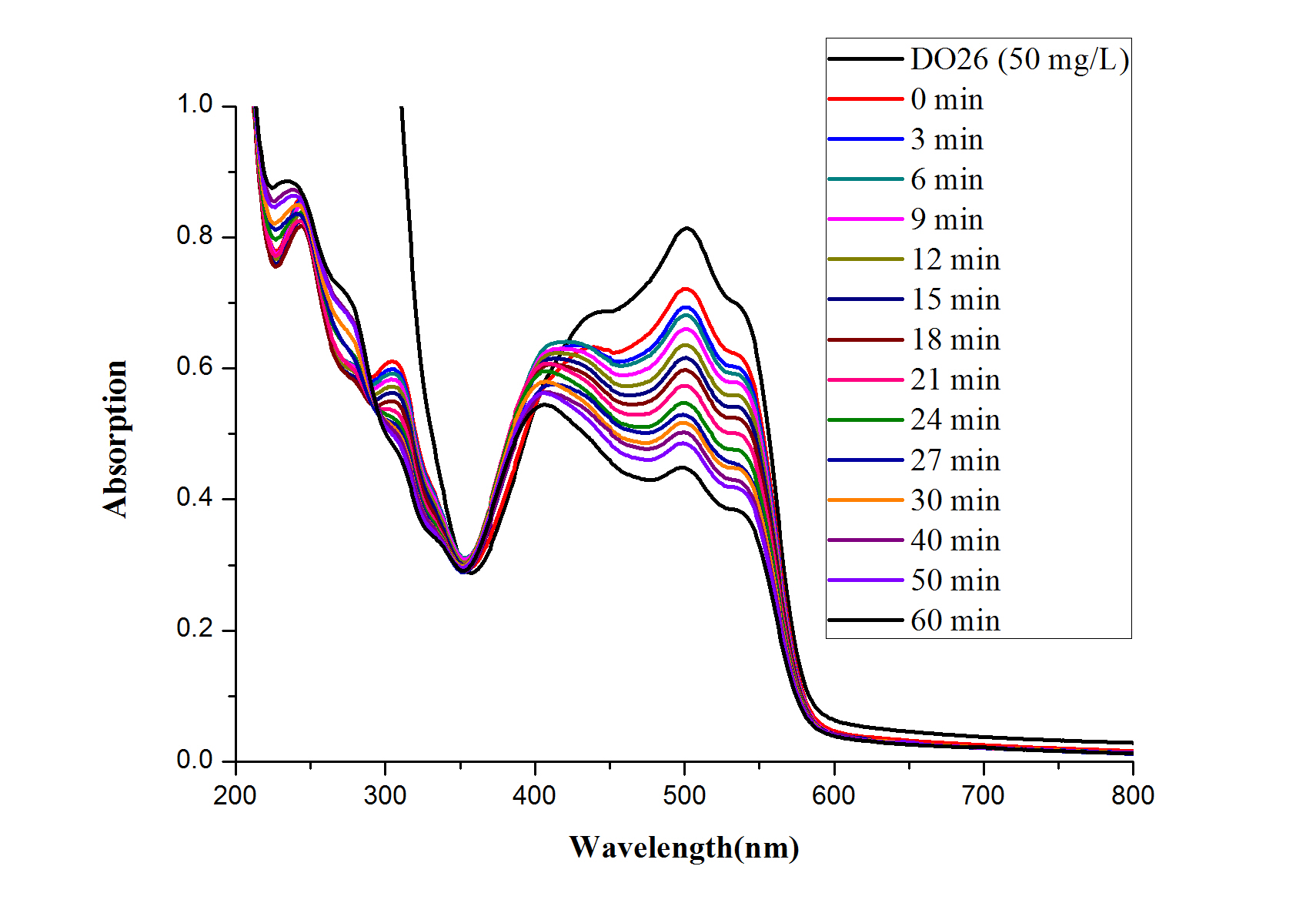

Supplement: S1 File — (ZIP) [file pone.0271408.s001.zip › supporting informations-PONE-D-22-11812/Figs5-12/DO26/dry AgNPs/10a 3mLDO26+0.1mL╕╔╓╞╬┤╚Ñ╞ñ╔·╜¬─╔├╫╥°ú¿╦«╠ß╬∩ú⌐-2.jpg]

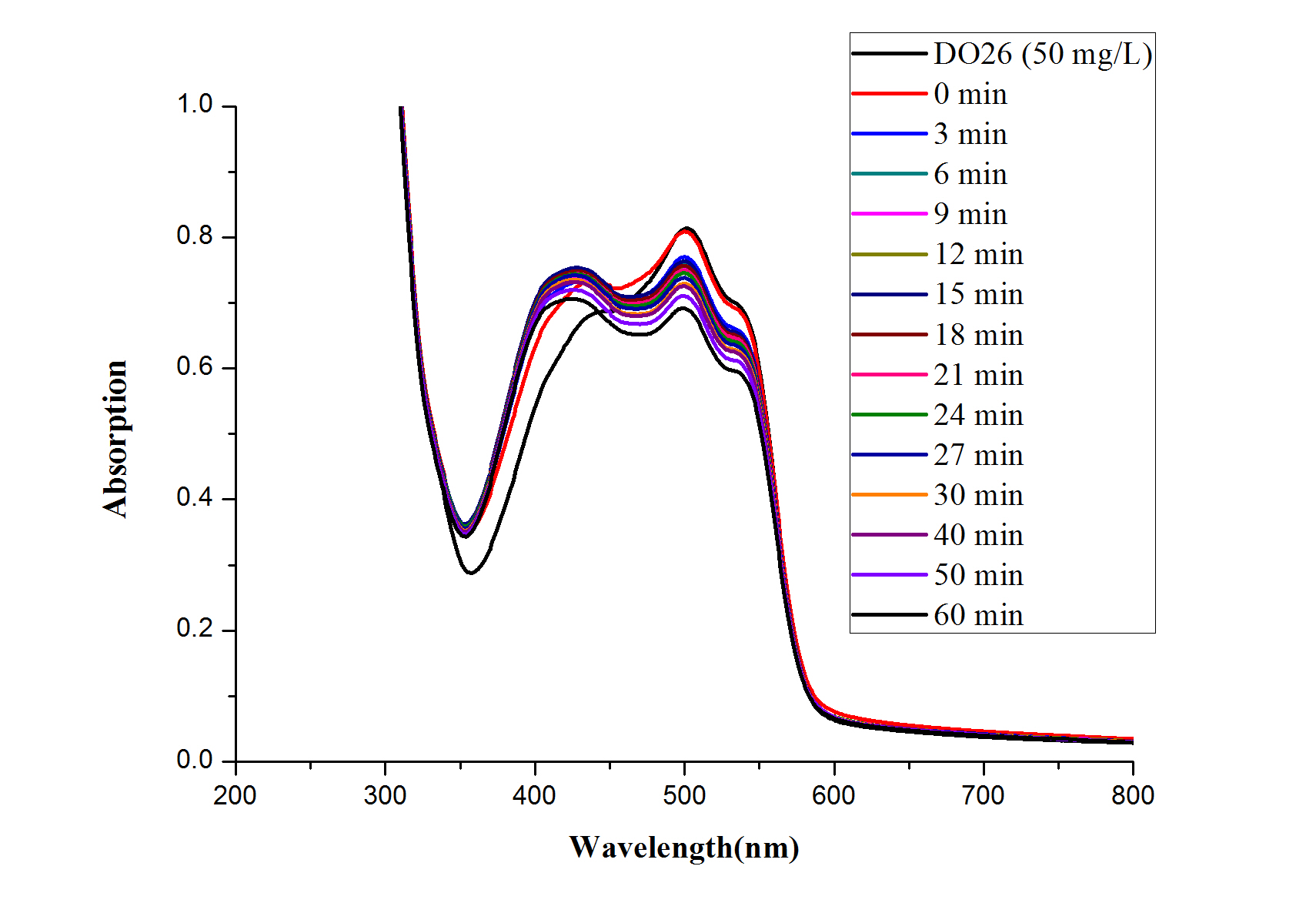

Supplement: S1 File — (ZIP) [file pone.0271408.s001.zip › supporting informations-PONE-D-22-11812/Figs5-12/DO26/dry AgNPs/10b 3mLDO26+0.1mL╕╔╓╞╚Ñ╞ñ╔·╜¬─╔├╫╥°ú¿╦«╠ß╬∩ú⌐-2.jpg]

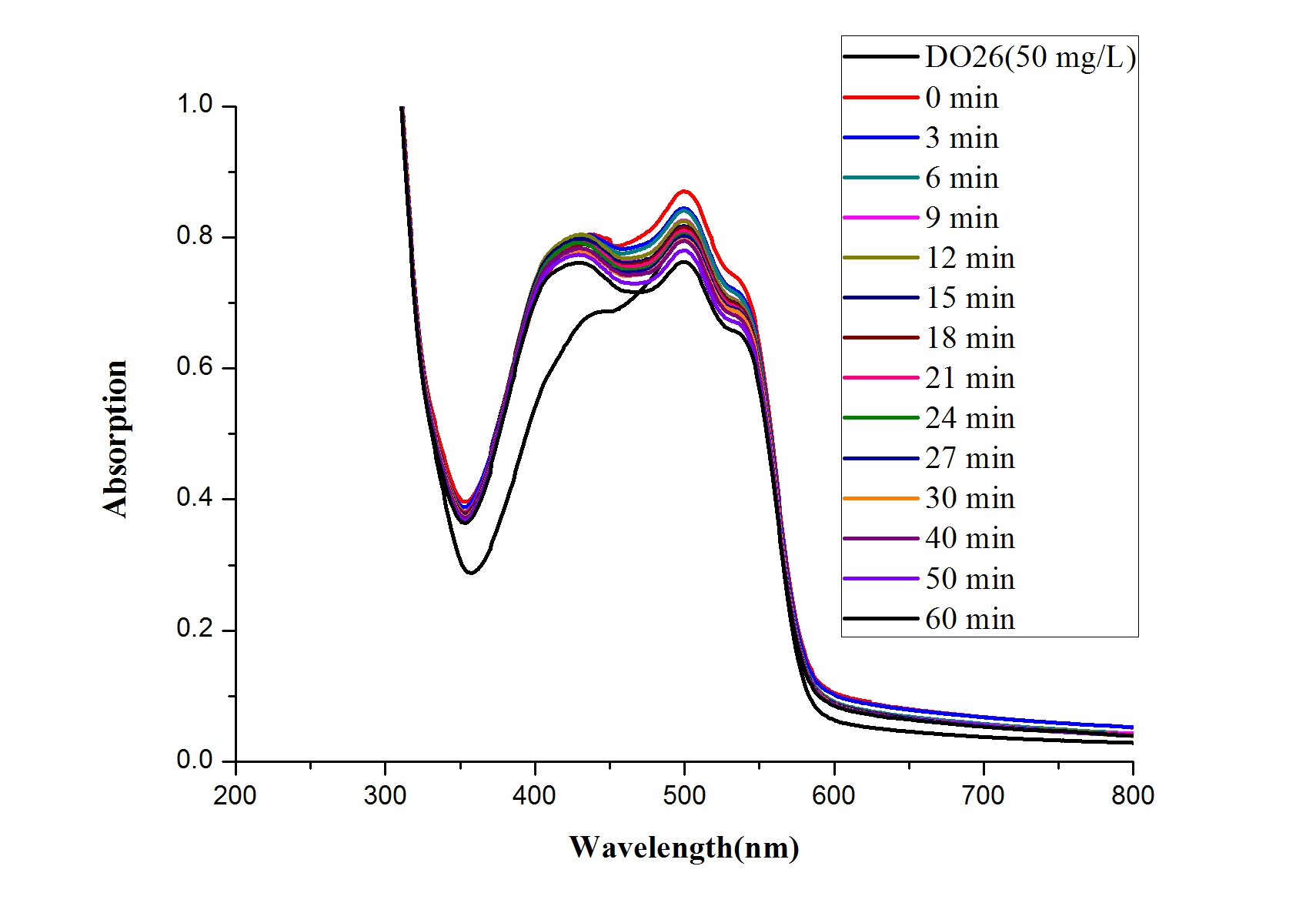

Supplement: S1 File — (ZIP) [file pone.0271408.s001.zip › supporting informations-PONE-D-22-11812/Figs5-12/DO26/dry AgNPs/10c 3mLDO26+0.1mL╕╔╓╞╔·╜¬╞ñ─╔├╫╥°ú¿╦«╠ß╬∩ú⌐-2.jpg]

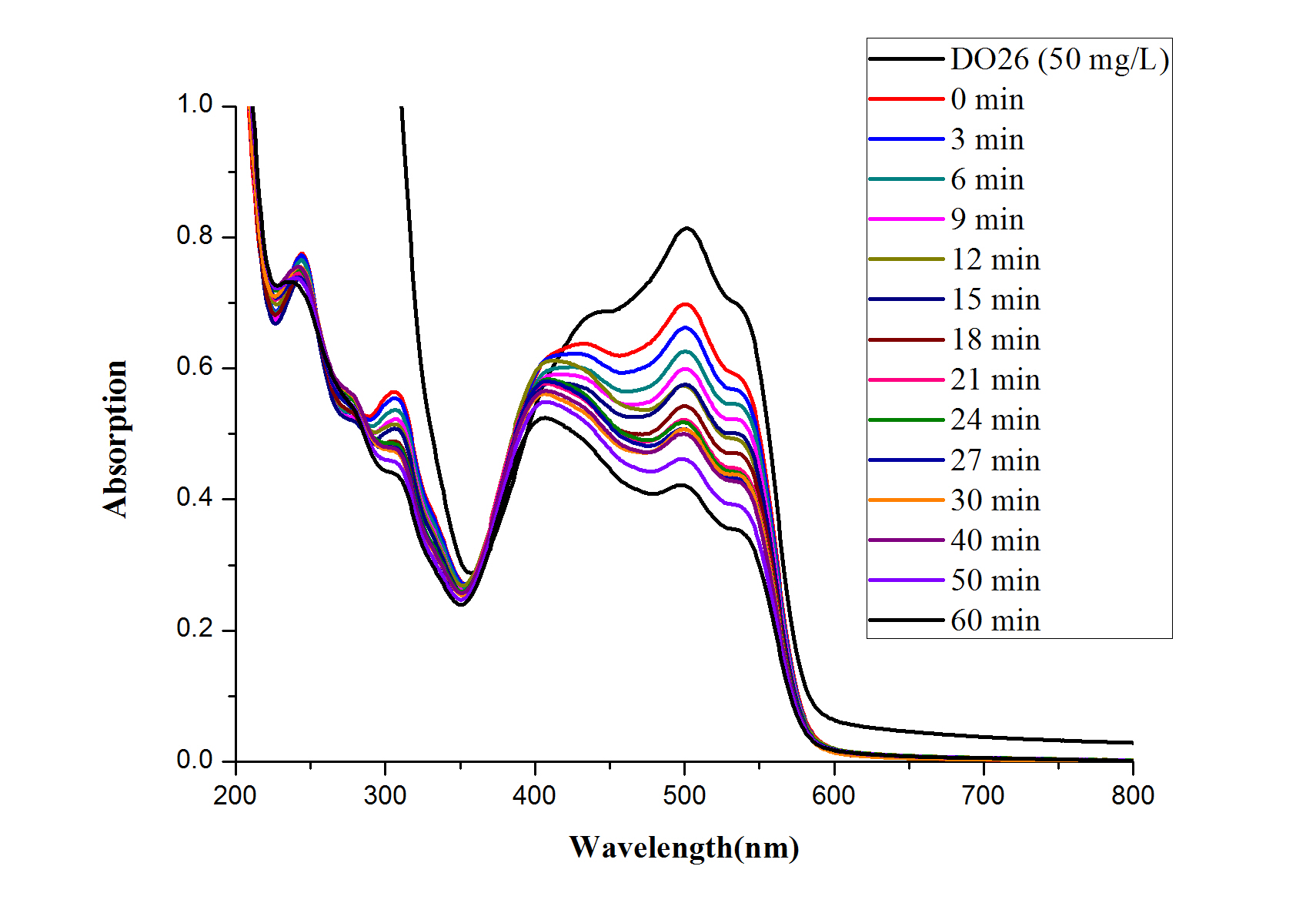

Supplement: S1 File — (ZIP) [file pone.0271408.s001.zip › supporting informations-PONE-D-22-11812/Figs5-12/DO26/dry AgNPs/10d 3mLDO26+0.1mL╕╔╓╞╬┤╚Ñ╞ñ─╔├╫╥°ú¿┤╝╠ß╬∩ú⌐-2.jpg]

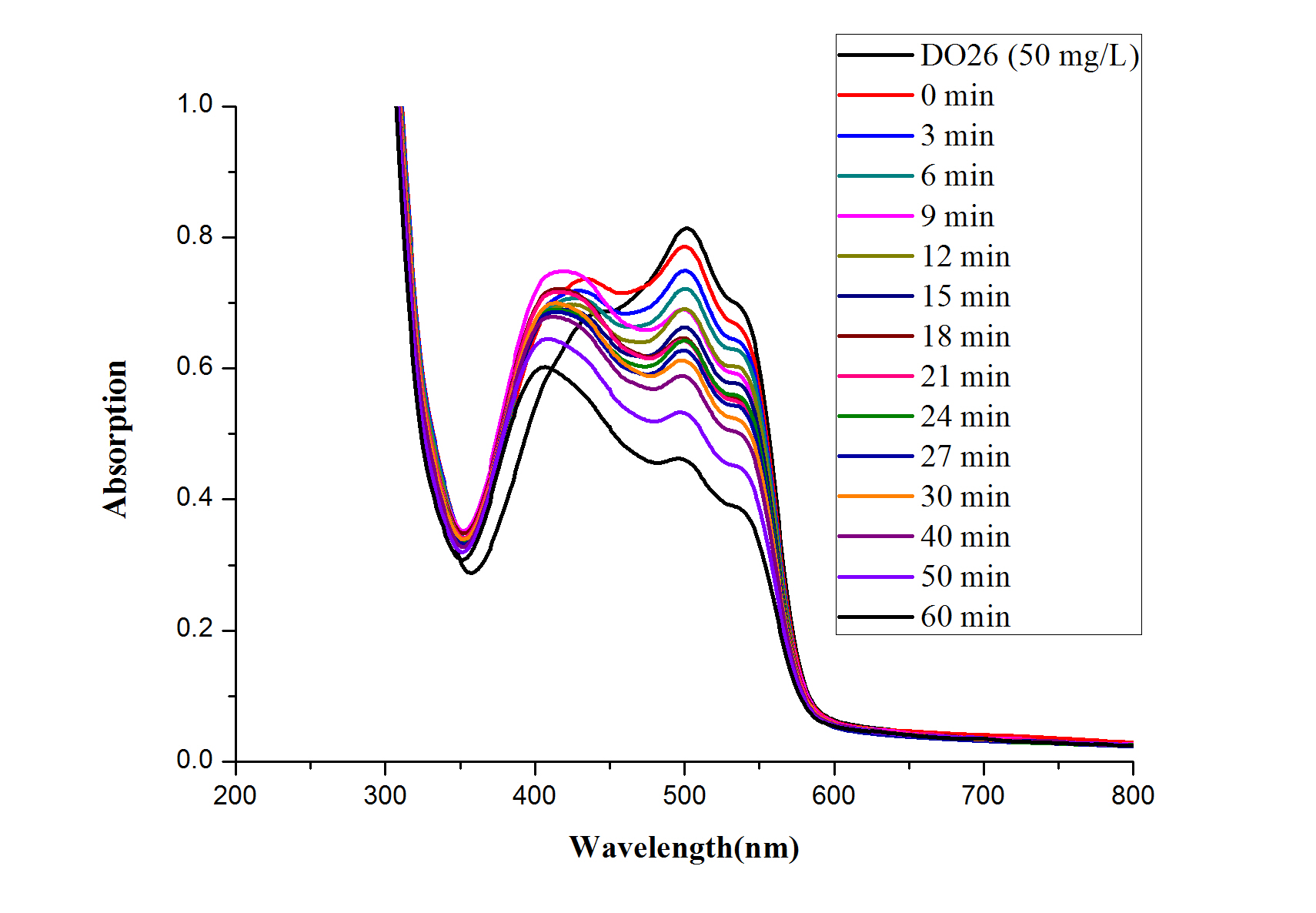

Supplement: S1 File — (ZIP) [file pone.0271408.s001.zip › supporting informations-PONE-D-22-11812/Figs5-12/DO26/dry AgNPs/10e 3mLDO26+0.1mL╕╔╓╞╚Ñ╞ñ╔·╜¬─╔├╫╥°ú¿┤╝╠ß╬∩ú⌐-2.jpg]

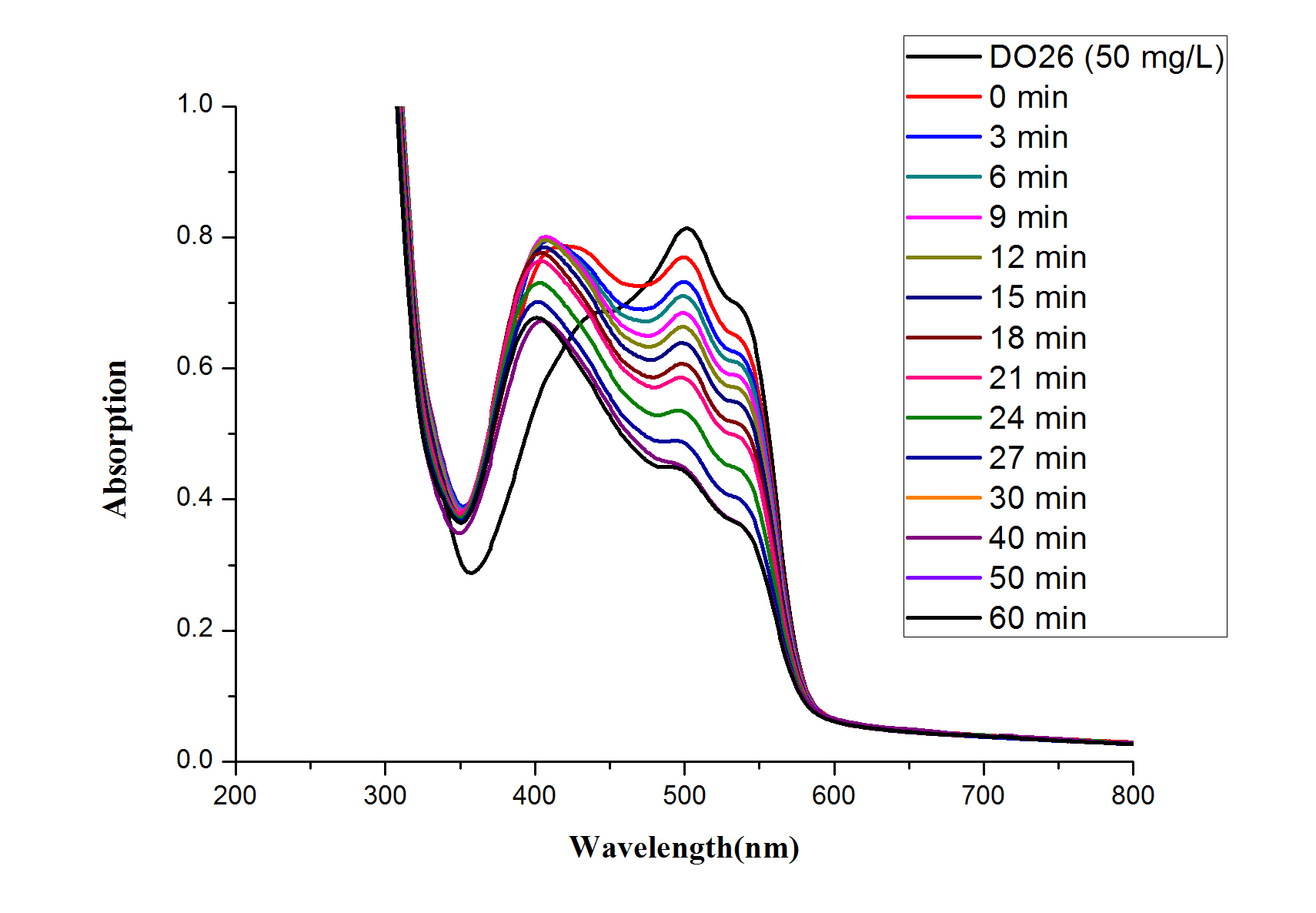

Supplement: S1 File — (ZIP) [file pone.0271408.s001.zip › supporting informations-PONE-D-22-11812/Figs5-12/DO26/dry AgNPs/10f 3mLDO26+0.1mL╕╔╓╞╔·╜¬╞ñ─╔├╫╥°ú¿┤╝╠ß╬∩ú⌐-2.jpg]

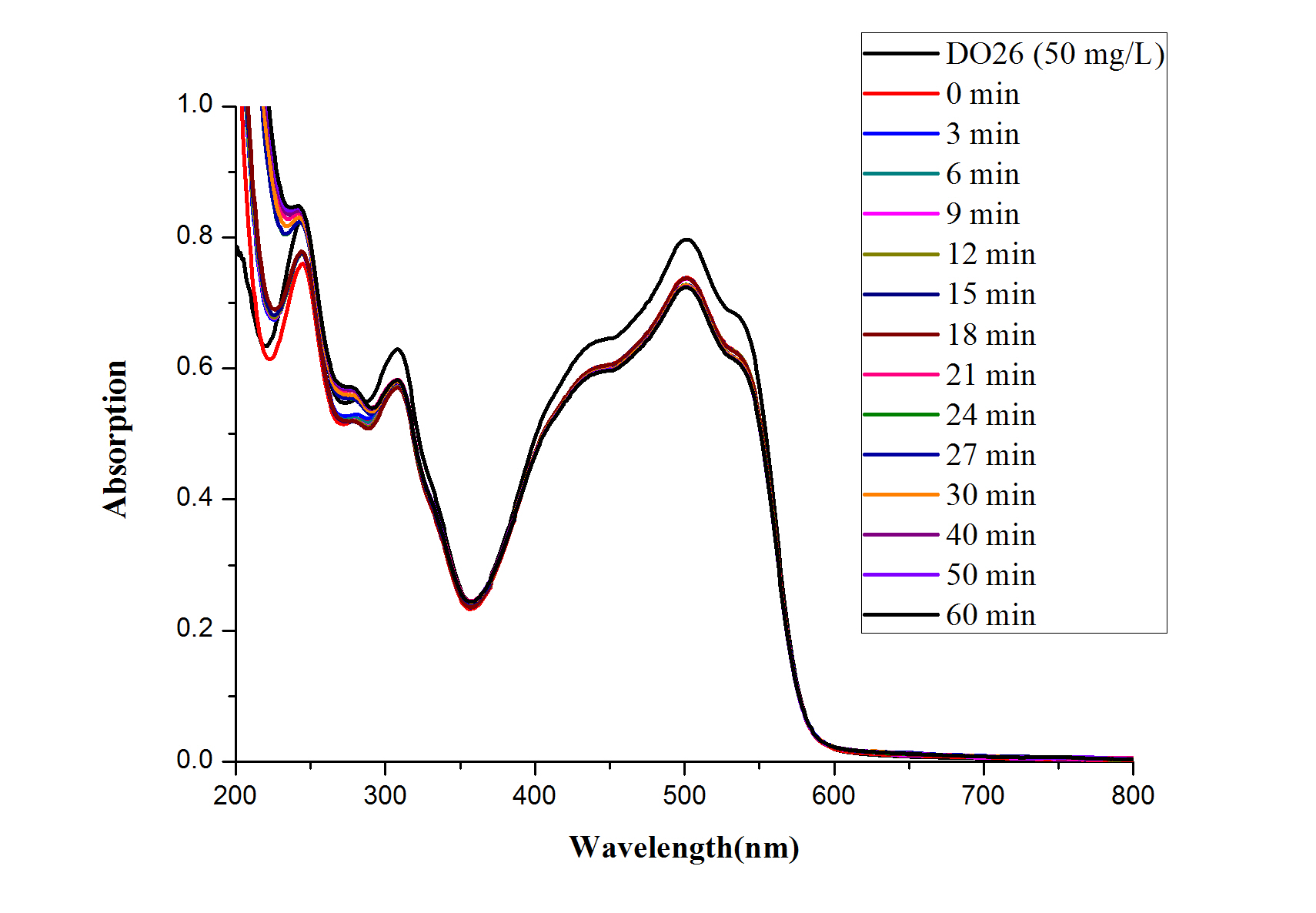

Supplement: S1 File — (ZIP) [file pone.0271408.s001.zip › supporting informations-PONE-D-22-11812/Figs5-12/DO26/dry extract/12a 3mLDO26+0.1mL╕╔╓╞╬┤╚Ñ╞ñ╔·╜¬ú¿╦«╠ß╬∩ú⌐-.jpg]

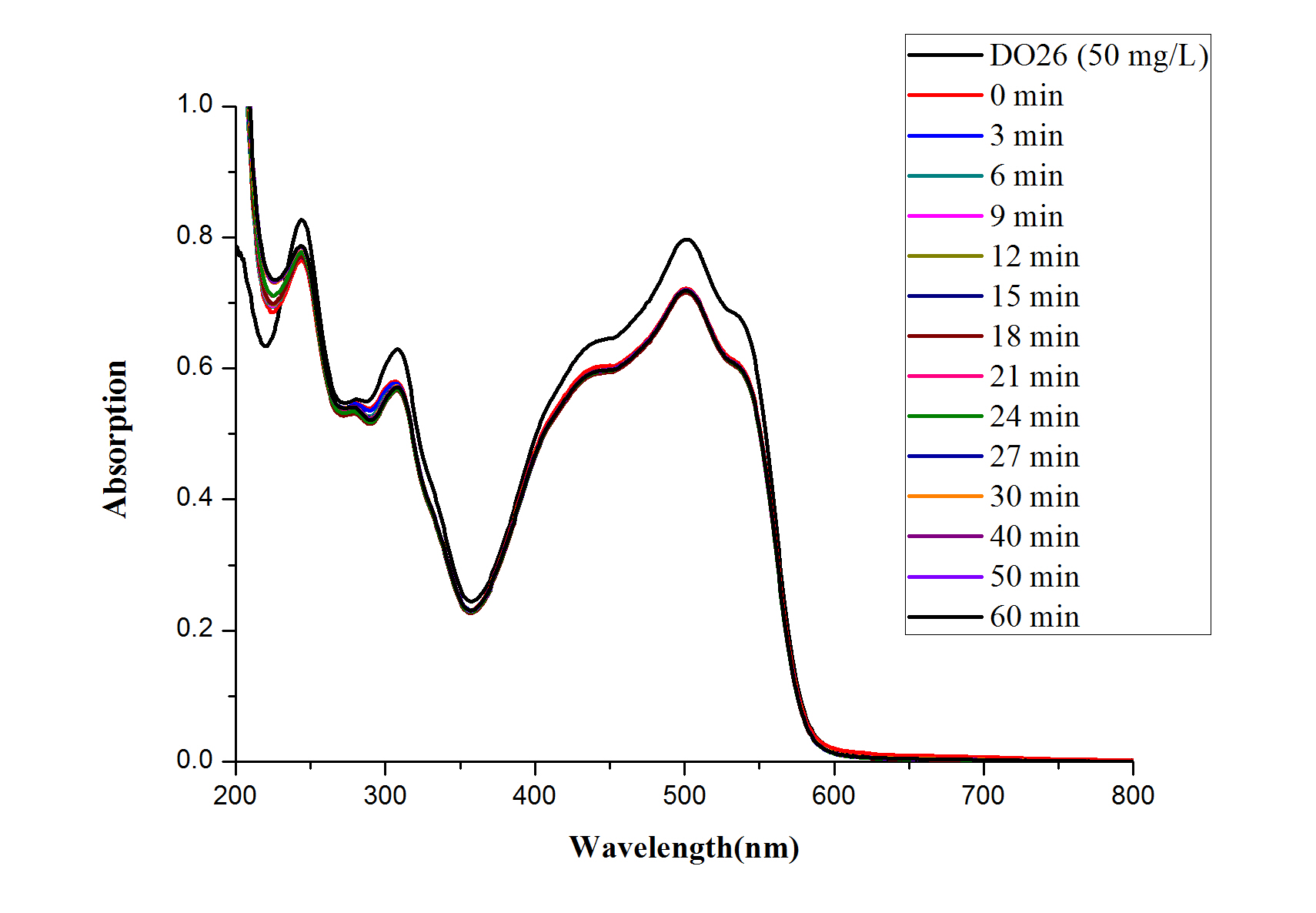

Supplement: S1 File — (ZIP) [file pone.0271408.s001.zip › supporting informations-PONE-D-22-11812/Figs5-12/DO26/dry extract/12b 3mLDO26+0.1mL╕╔╓╞╚Ñ╞ñ╔·╜¬ú¿╦«╠ß╬∩ú⌐-.jpg]

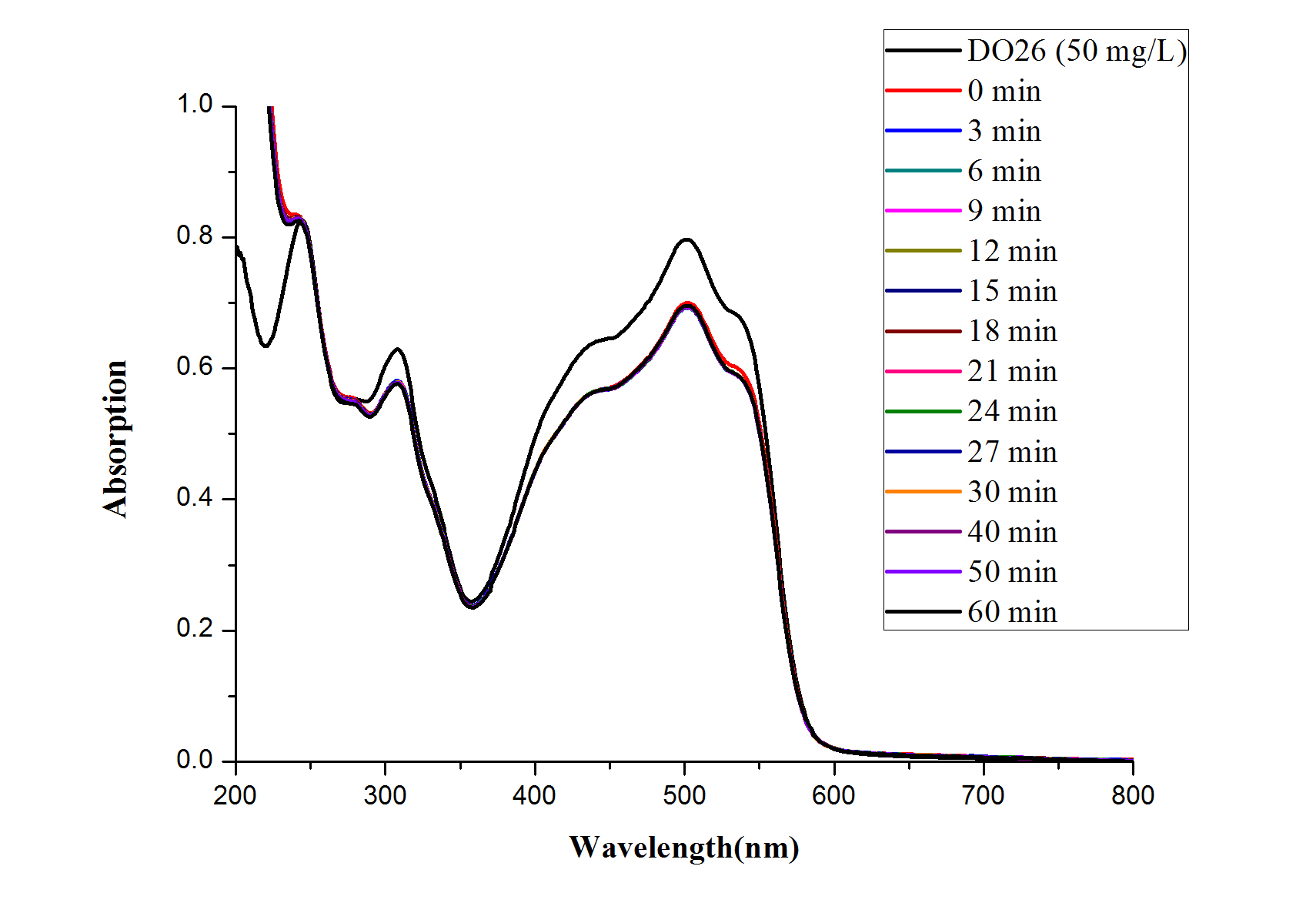

Supplement: S1 File — (ZIP) [file pone.0271408.s001.zip › supporting informations-PONE-D-22-11812/Figs5-12/DO26/dry extract/12c 3mLDO26+0.1mL╕╔╓╞╔·╜¬╞ñ╦«╠ß╬∩.jpg]

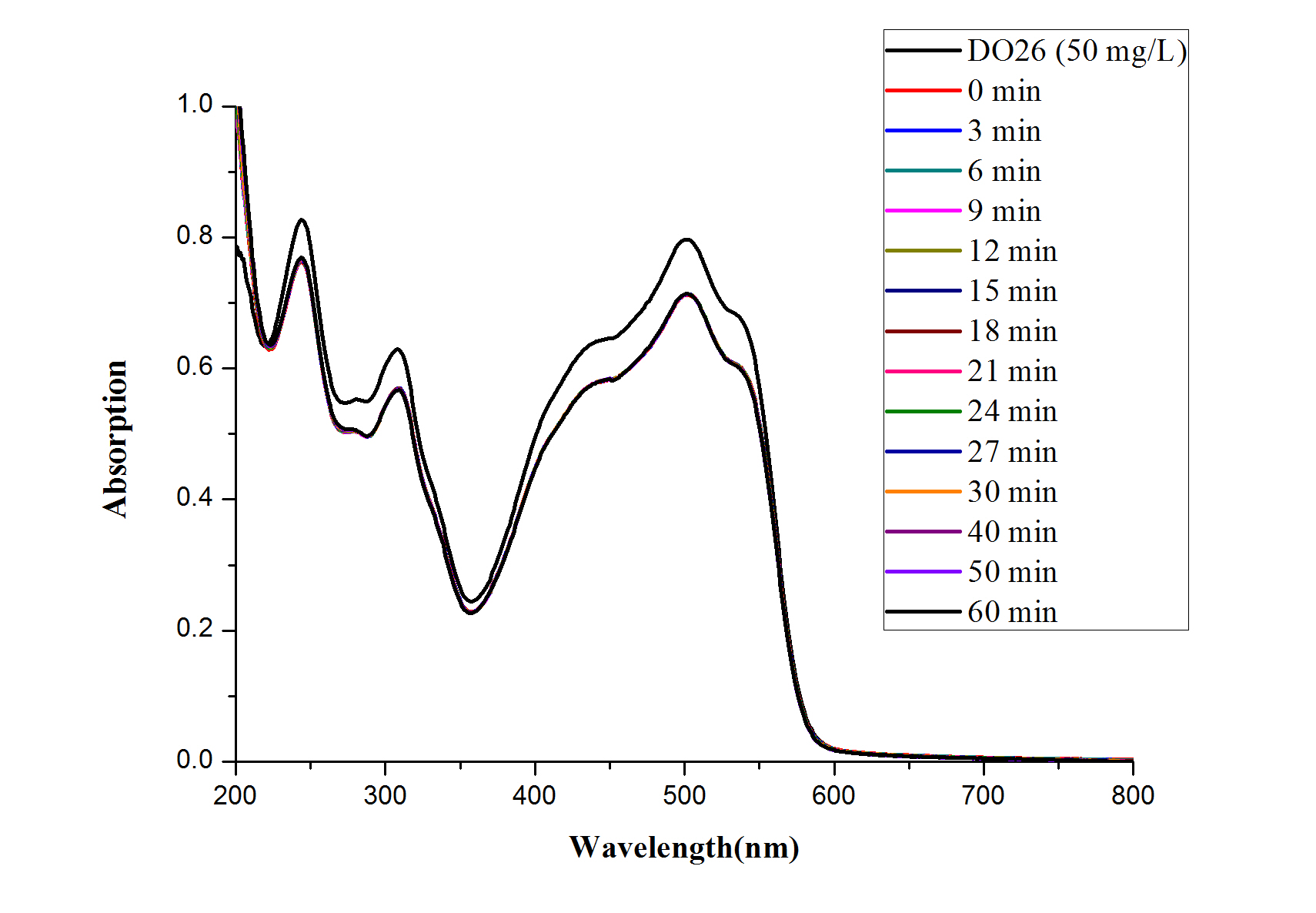

Supplement: S1 File — (ZIP) [file pone.0271408.s001.zip › supporting informations-PONE-D-22-11812/Figs5-12/DO26/dry extract/12d 3mLDO26+0.1mL╕╔╓╞╬┤╚Ñ╞ñ╔·╜¬┤╝╠ß╬∩.jpg]

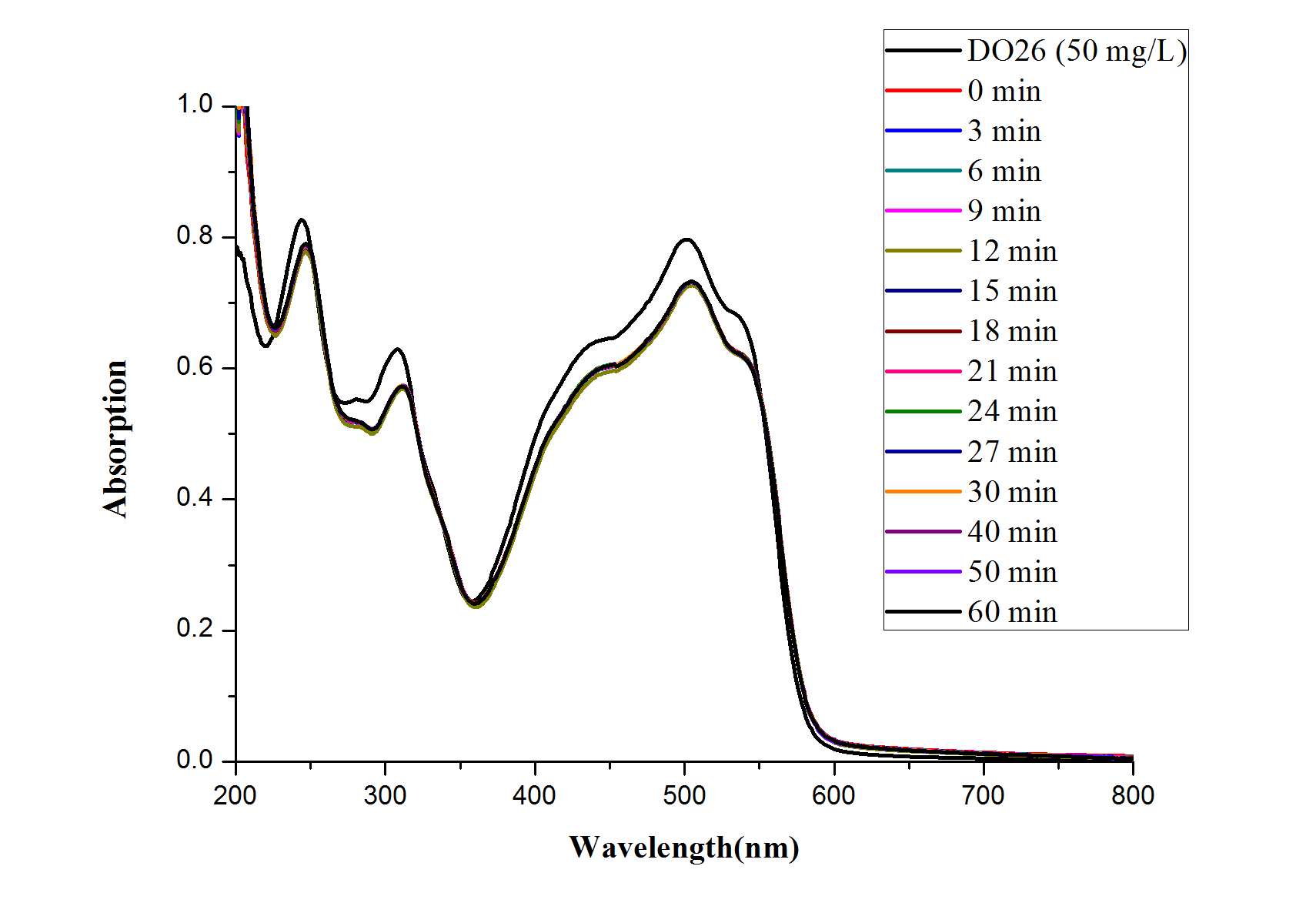

Supplement: S1 File — (ZIP) [file pone.0271408.s001.zip › supporting informations-PONE-D-22-11812/Figs5-12/DO26/dry extract/12e 3mLDO26+0.1mL╕╔╓╞╚Ñ╞ñ╔·╜¬┤╝╠ß╬∩.jpg]

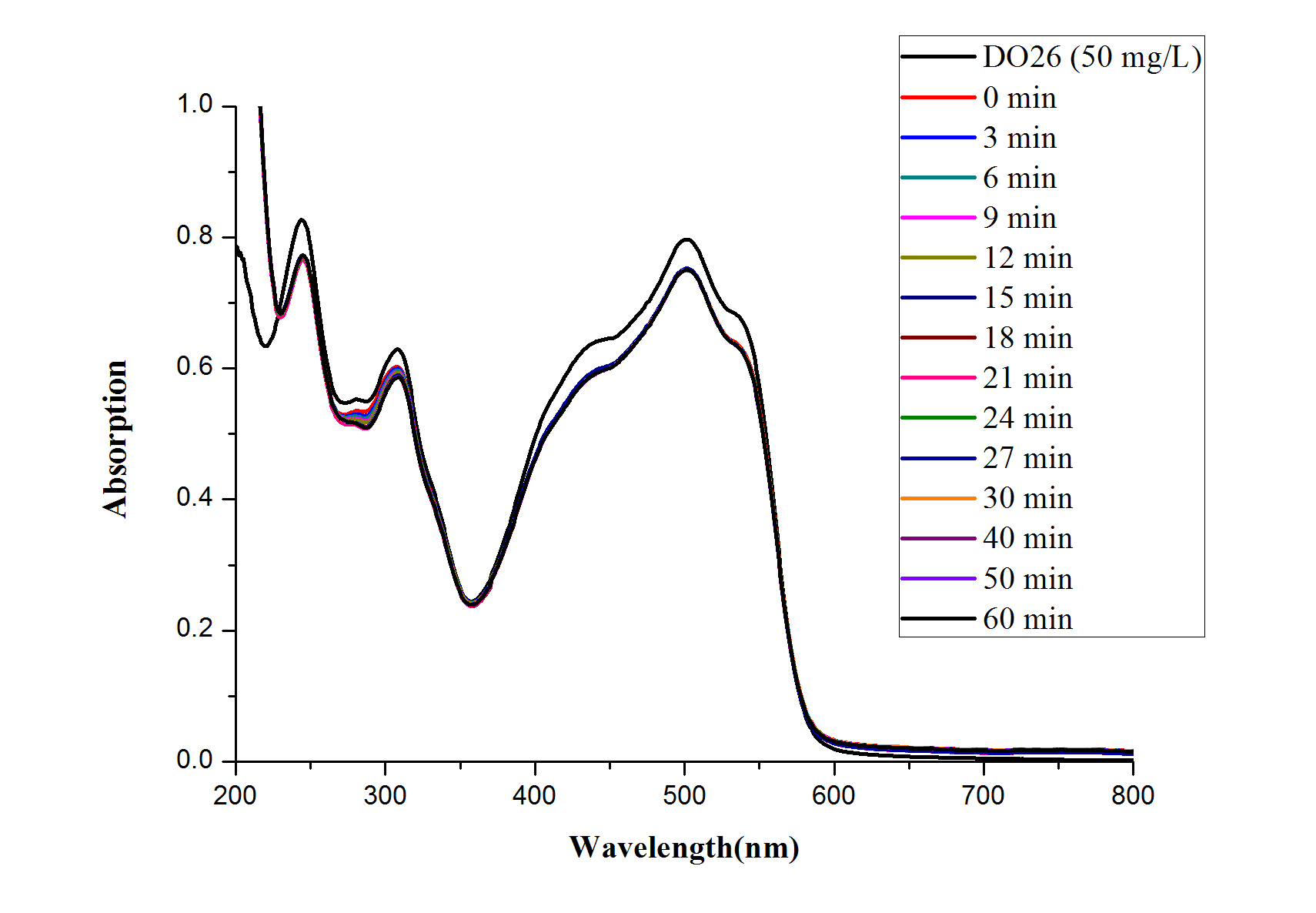

Supplement: S1 File — (ZIP) [file pone.0271408.s001.zip › supporting informations-PONE-D-22-11812/Figs5-12/DO26/dry extract/12f 3mLDO26+0.1mL╕╔╓╞╔·╜¬╞ñú¿┤╝╠ß╬∩ú⌐-.jpg]

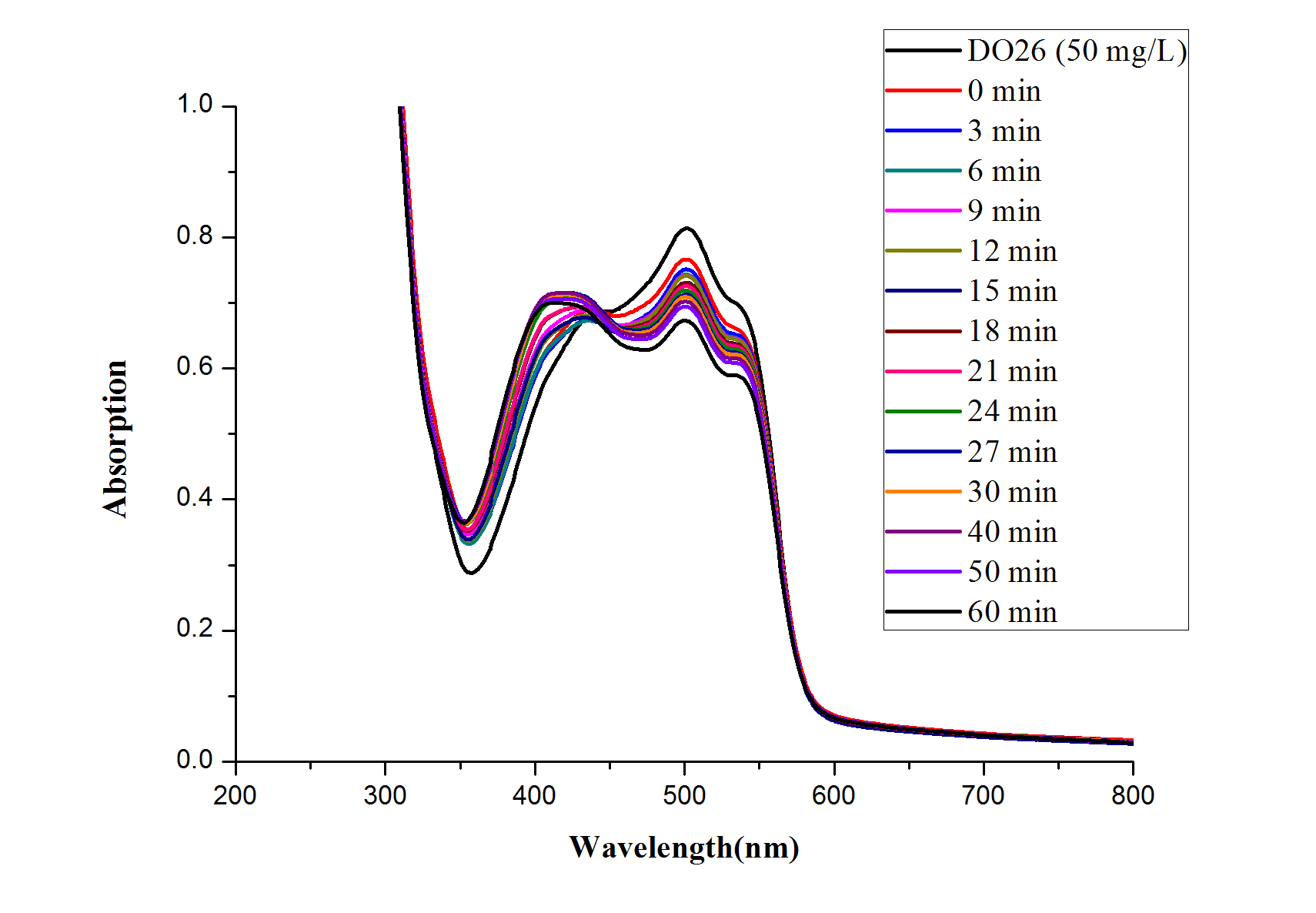

Supplement: S1 File — (ZIP) [file pone.0271408.s001.zip › supporting informations-PONE-D-22-11812/Figs5-12/DO26/fresh AgNPs/9a 3mLDO26+0.1mL╨┬╧╩╬┤╚Ñ╞ñ╔·╜¬─╔├╫╥°ú¿╦«╠ß╬∩ú⌐-2.jpg]

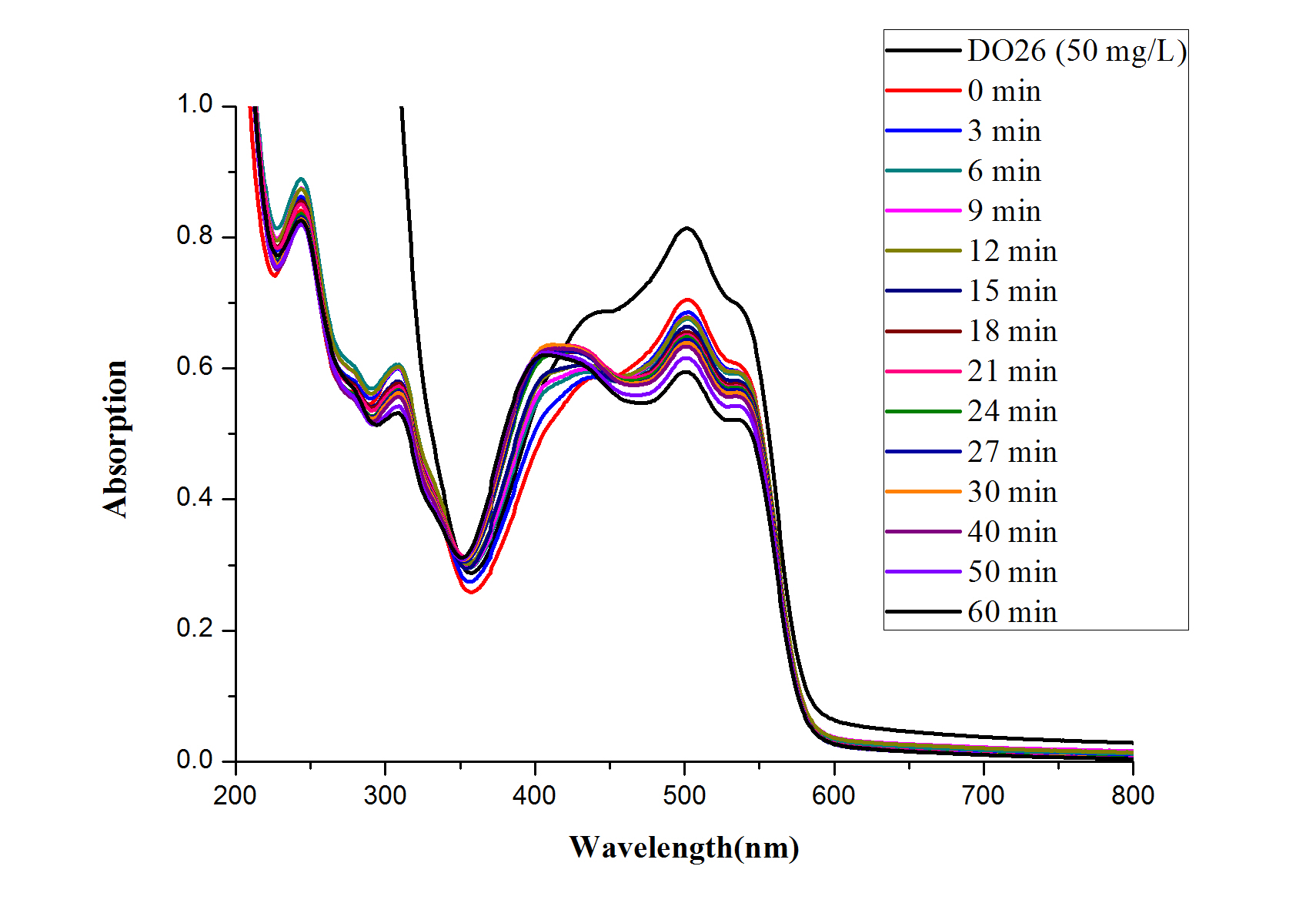

Supplement: S1 File — (ZIP) [file pone.0271408.s001.zip › supporting informations-PONE-D-22-11812/Figs5-12/DO26/fresh AgNPs/9b 3mLDO26+0.1mL╨┬╧╩╚Ñ╞ñ╔·╜¬─╔├╫╥°ú¿╦«╠ß╬∩ú⌐-2.jpg]

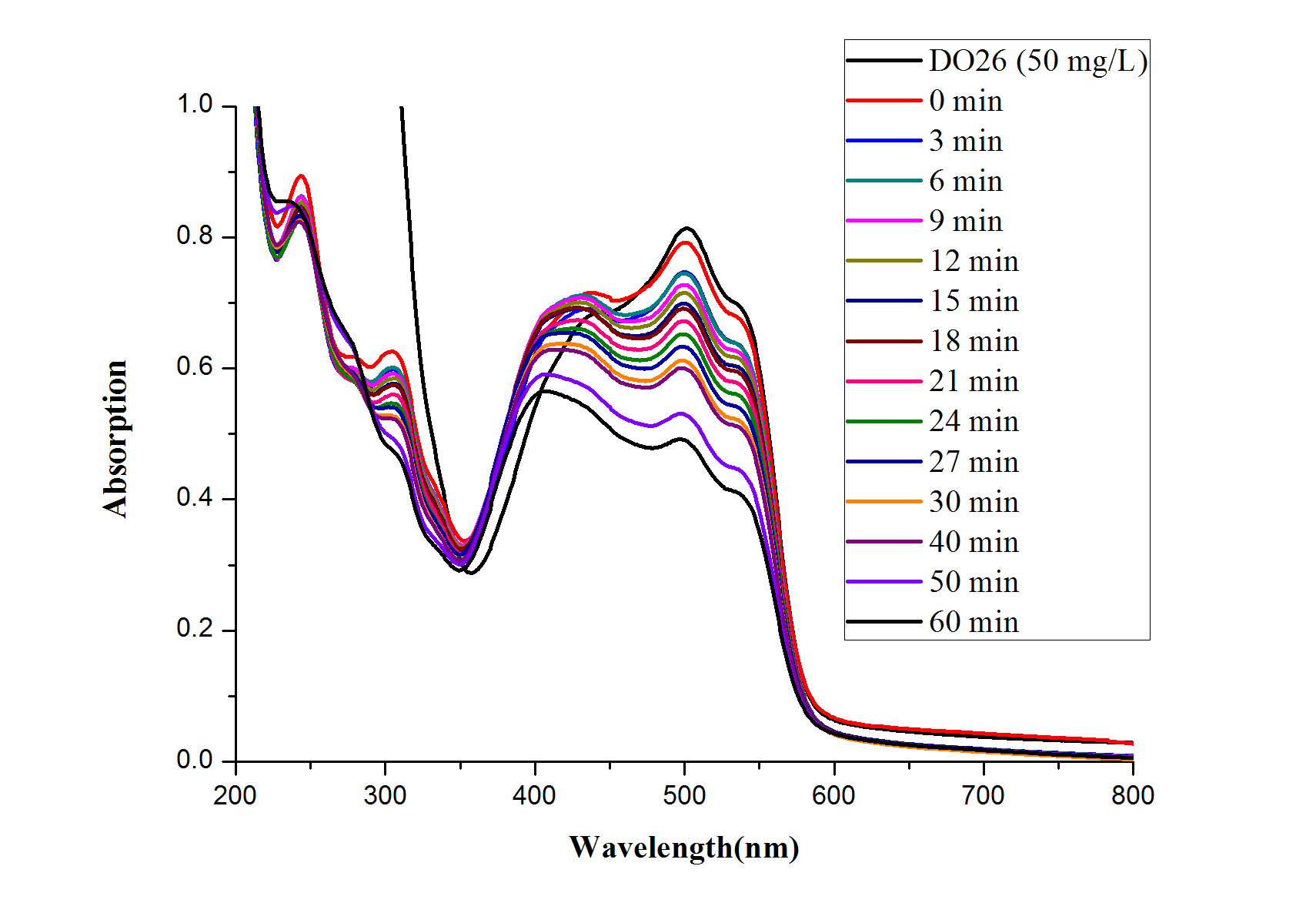

Supplement: S1 File — (ZIP) [file pone.0271408.s001.zip › supporting informations-PONE-D-22-11812/Figs5-12/DO26/fresh AgNPs/9c 3mLDO26+0.1mL╨┬╧╩╔·╜¬╞ñ─╔├╫╥°ú¿╦«ú⌐-2.jpg]

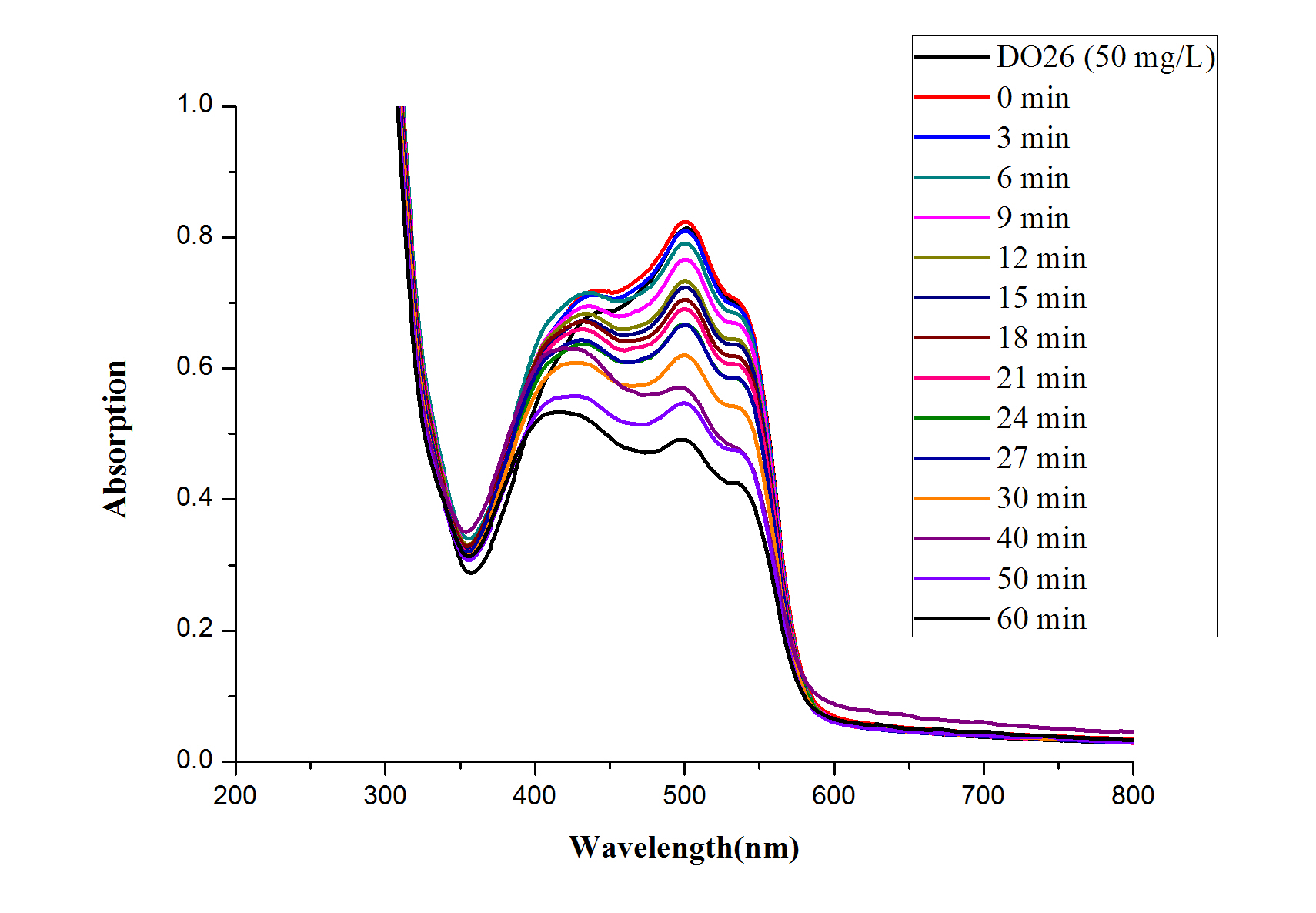

Supplement: S1 File — (ZIP) [file pone.0271408.s001.zip › supporting informations-PONE-D-22-11812/Figs5-12/DO26/fresh AgNPs/9d 3mLDO26+0.1mL╨┬╧╩╬┤╚Ñ╞ñ╔·╜¬─╔├╫╥°ú¿┤╝ú⌐-2.jpg]

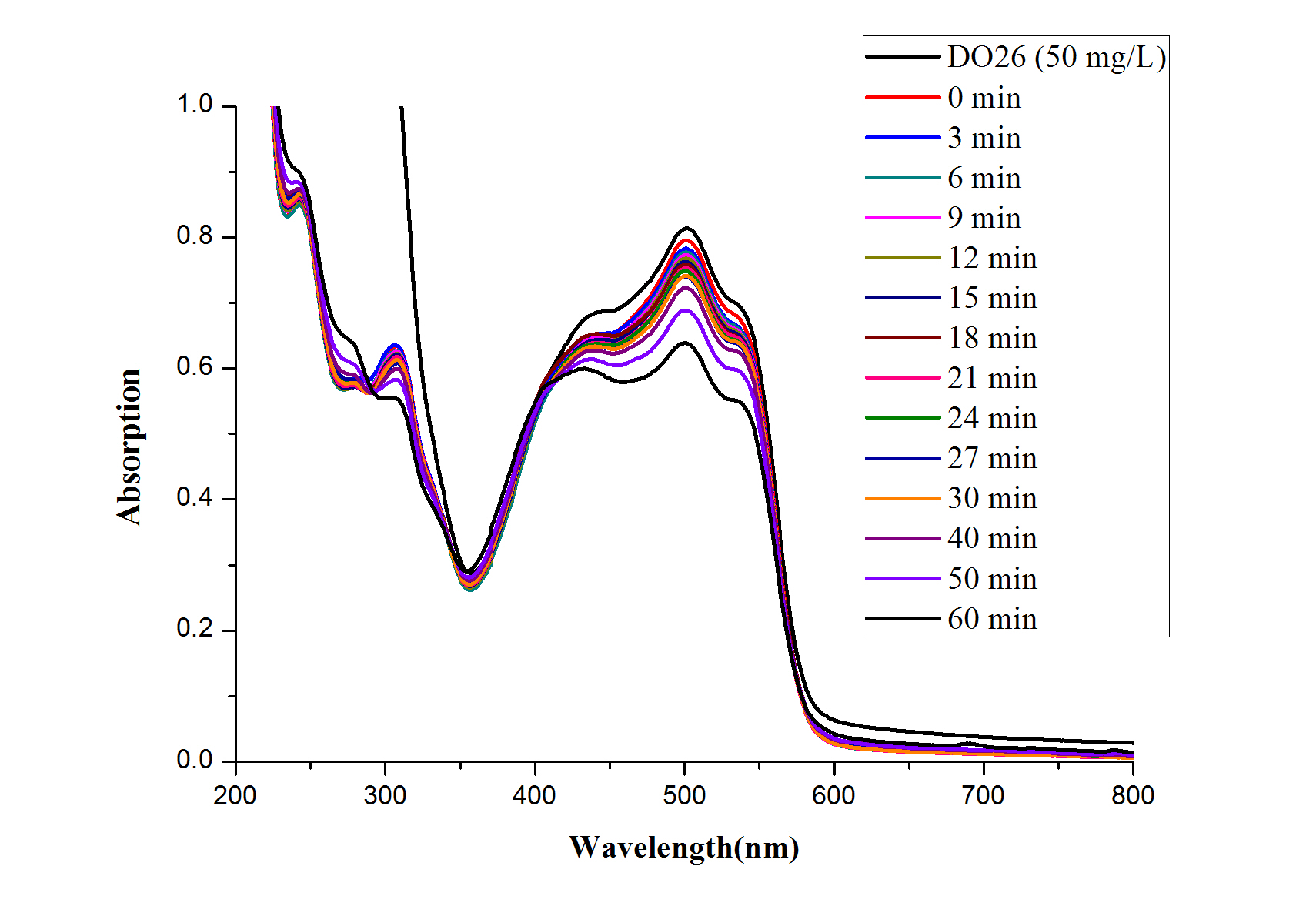

Supplement: S1 File — (ZIP) [file pone.0271408.s001.zip › supporting informations-PONE-D-22-11812/Figs5-12/DO26/fresh AgNPs/9e 3mLDO26+0.1mL╨┬╧╩╚Ñ╞ñ╔·╜¬─╔├╫╥°ú¿┤╝ú⌐-2.jpg]

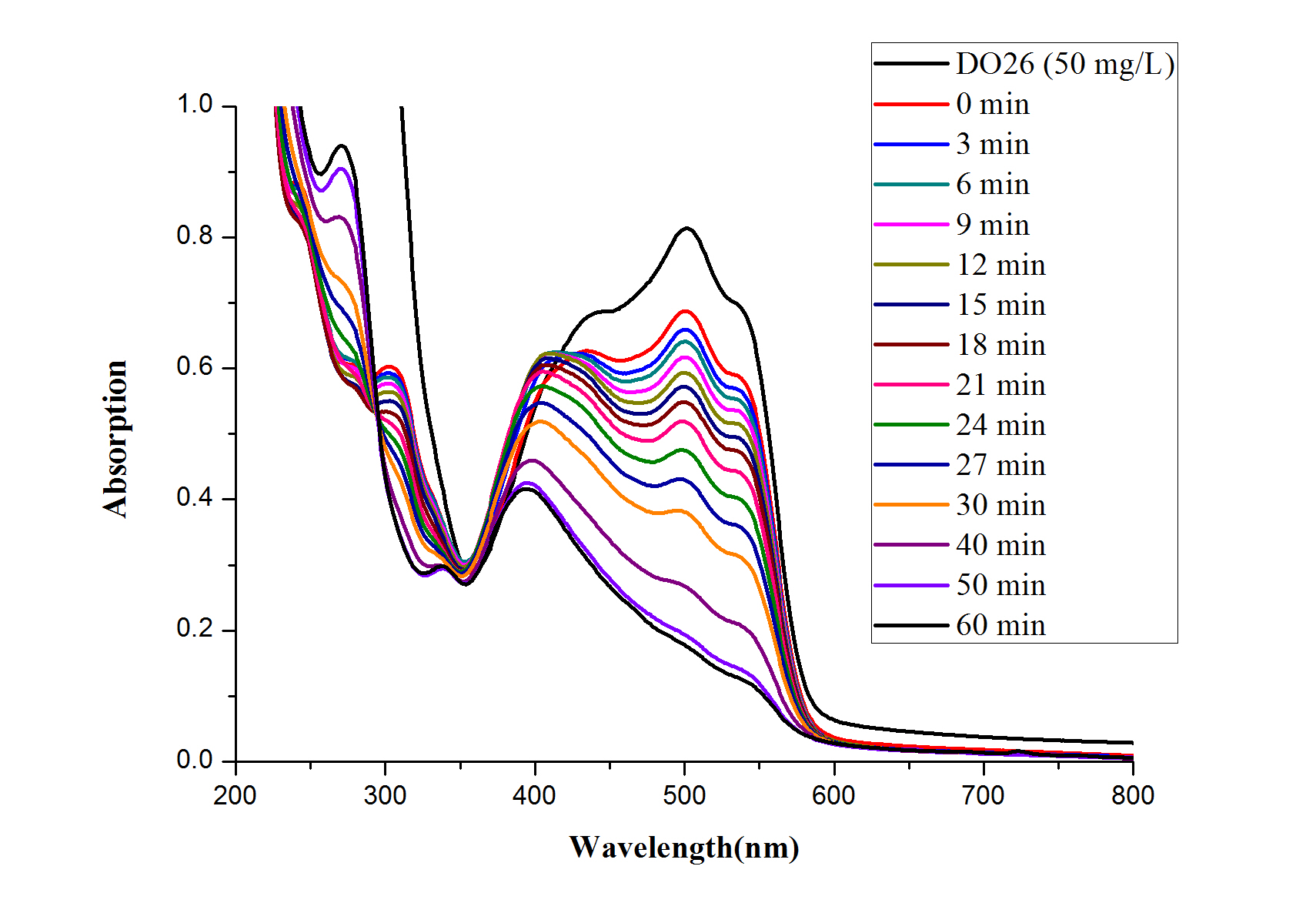

Supplement: S1 File — (ZIP) [file pone.0271408.s001.zip › supporting informations-PONE-D-22-11812/Figs5-12/DO26/fresh AgNPs/9f 3mLDO26+0.1mL╨┬╧╩╔·╜¬╞ñ─╔├╫╥°ú¿┤╝ú⌐-2.jpg]

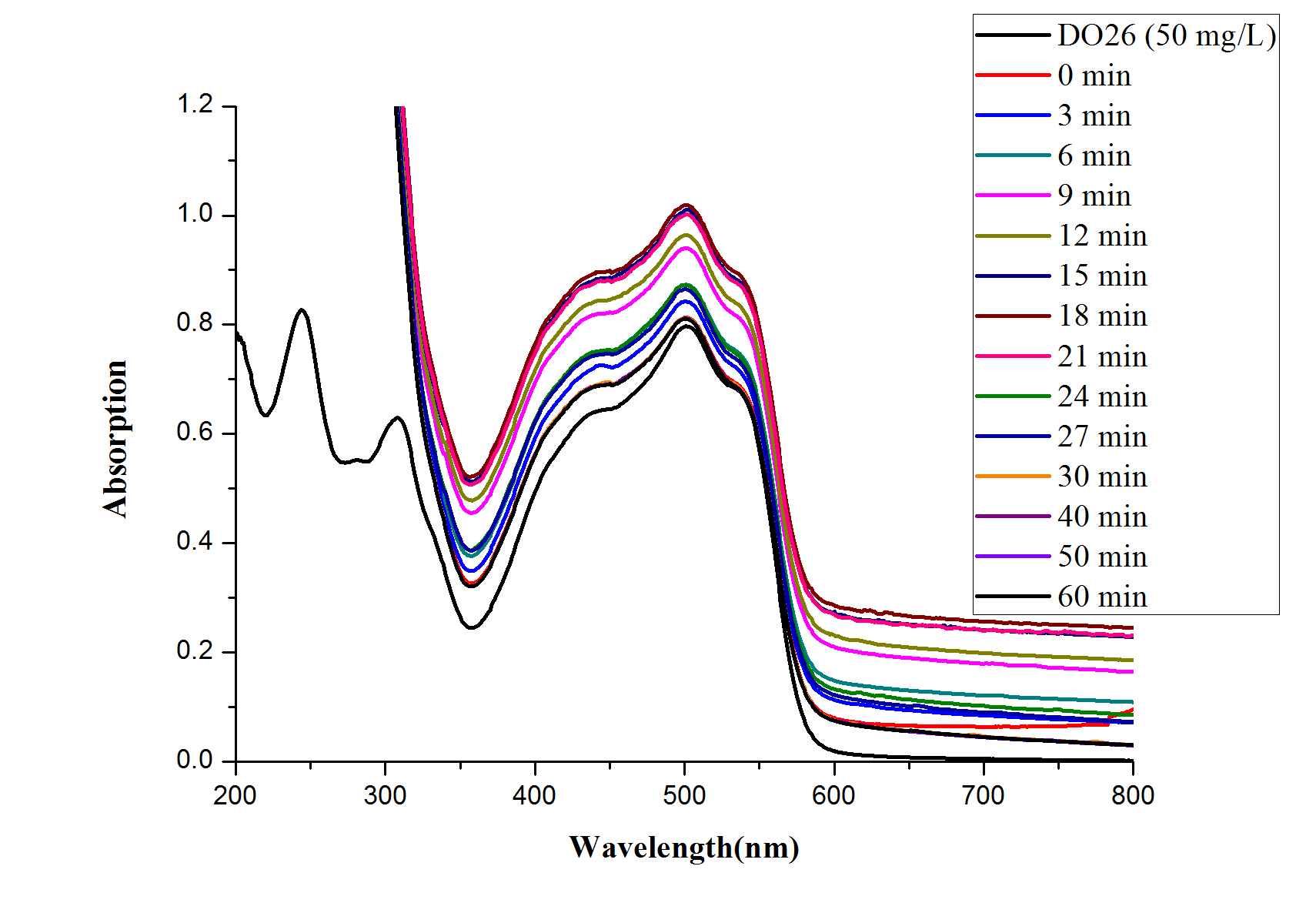

Supplement: S1 File — (ZIP) [file pone.0271408.s001.zip › supporting informations-PONE-D-22-11812/Figs5-12/DO26/fresh extract/11a 3mLDO26+0.1mL╨┬╧╩╬┤╚Ñ╞ñ╔·╜¬╦«╠ß╚í╬∩-.jpg]

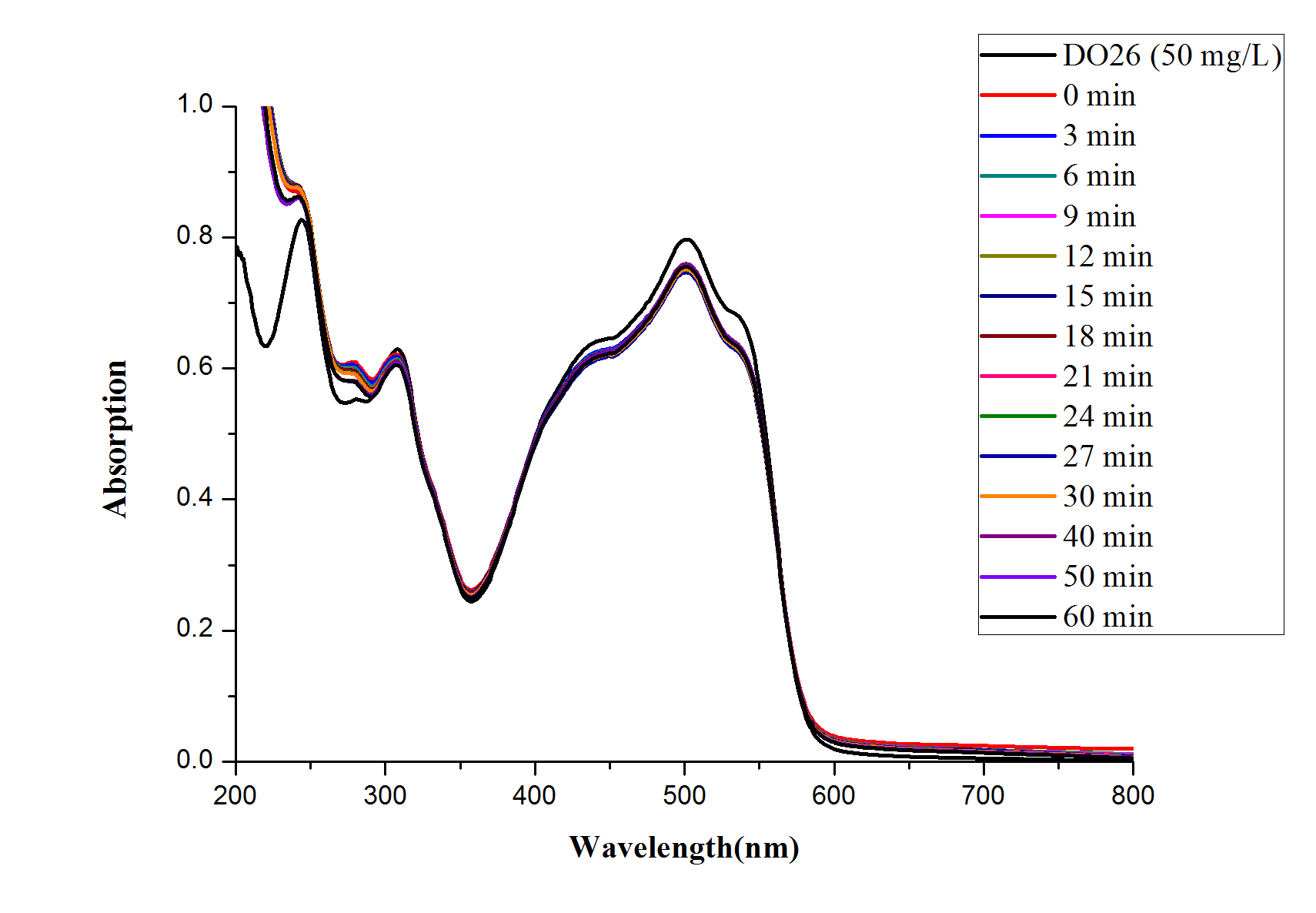

Supplement: S1 File — (ZIP) [file pone.0271408.s001.zip › supporting informations-PONE-D-22-11812/Figs5-12/DO26/fresh extract/11b 3mLDO26+0.1mL╨┬╧╩╚Ñ╞ñ╔·╜¬╦«╠ß╚í╬∩-.jpg]

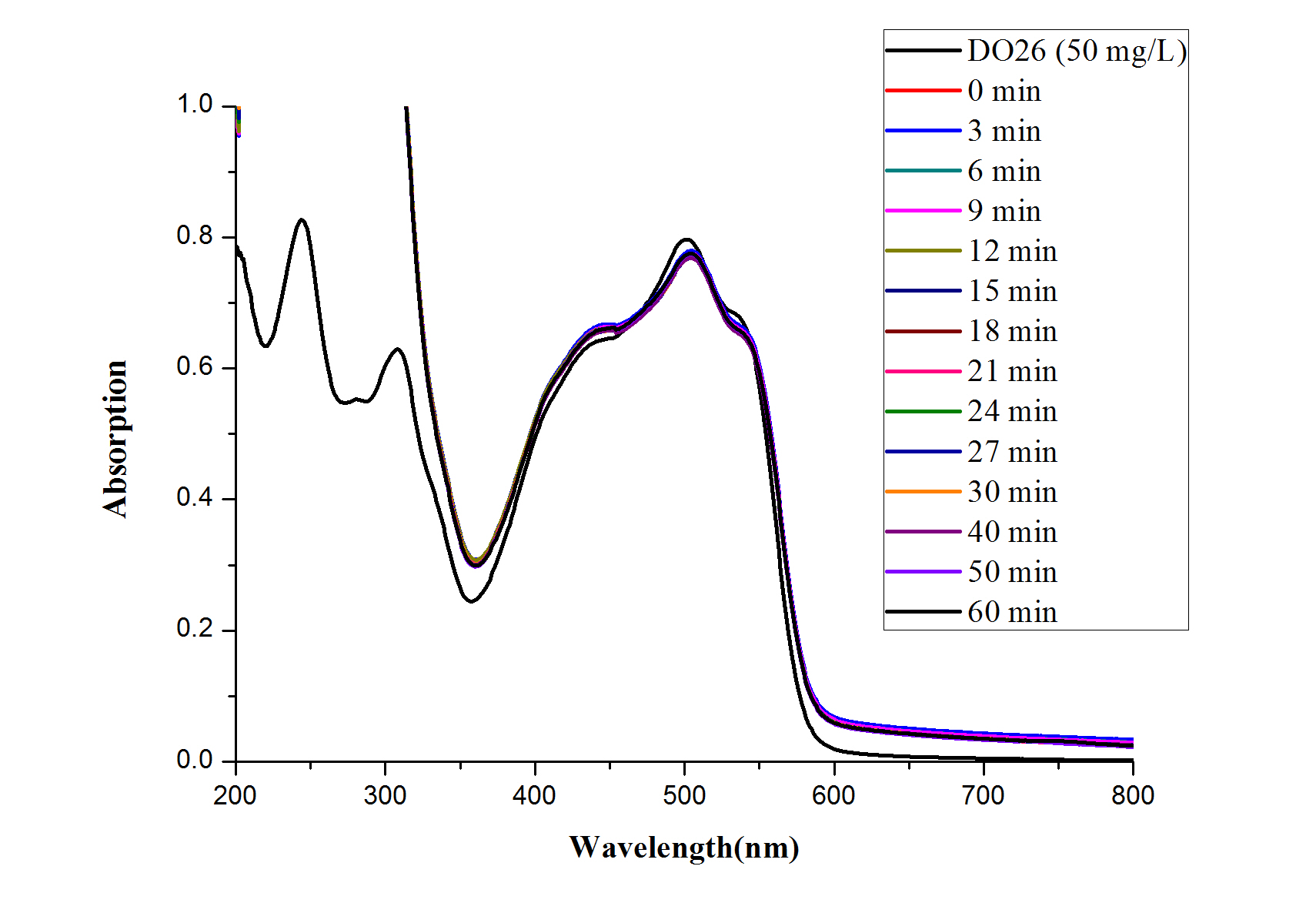

Supplement: S1 File — (ZIP) [file pone.0271408.s001.zip › supporting informations-PONE-D-22-11812/Figs5-12/DO26/fresh extract/11c 3mLDO26+0.1mL╨┬╧╩╔·╜¬╞ñ╦«╠ß╬∩.jpg]

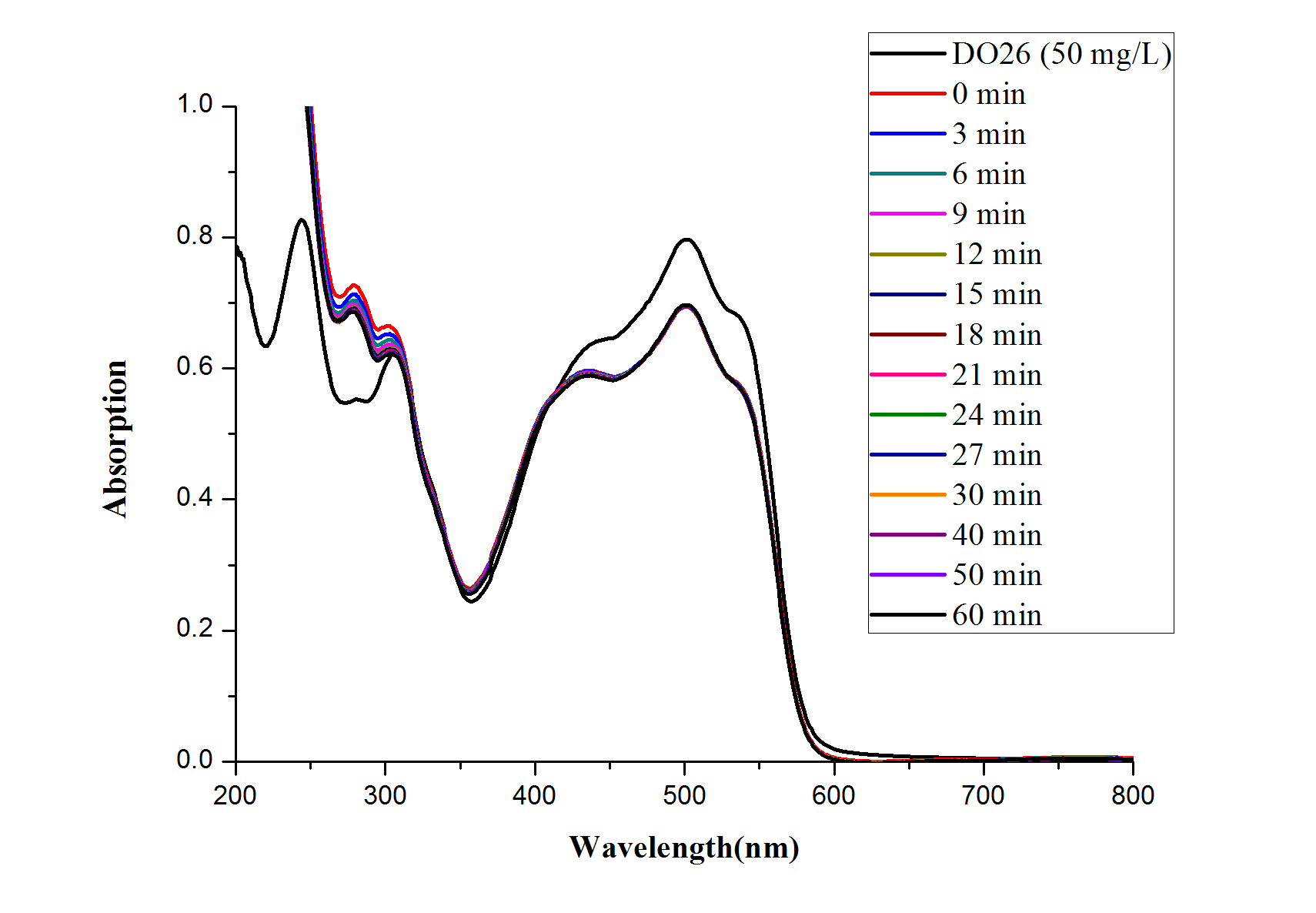

Supplement: S1 File — (ZIP) [file pone.0271408.s001.zip › supporting informations-PONE-D-22-11812/Figs5-12/DO26/fresh extract/11d 3mLDO26+0.1mL╨┬╧╩╬┤╚Ñ╞ñ╔·╜¬┤╝╠ß╚í╬∩-.jpg]

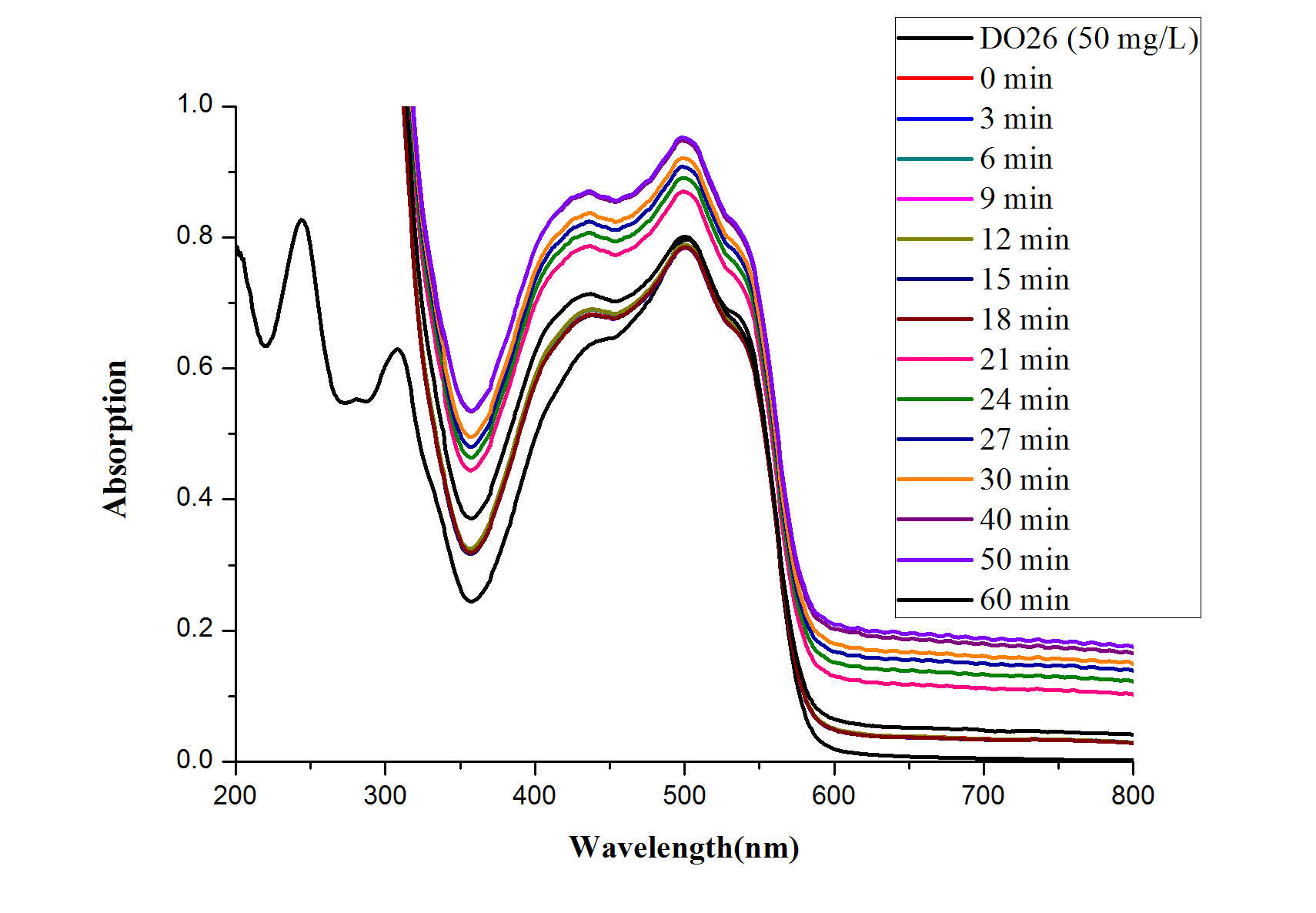

Supplement: S1 File — (ZIP) [file pone.0271408.s001.zip › supporting informations-PONE-D-22-11812/Figs5-12/DO26/fresh extract/11e 3mLDO26+0.1mL╨┬╧╩╚Ñ╞ñ╔·╜¬┤╝╠ß╚í╬∩-.jpg]

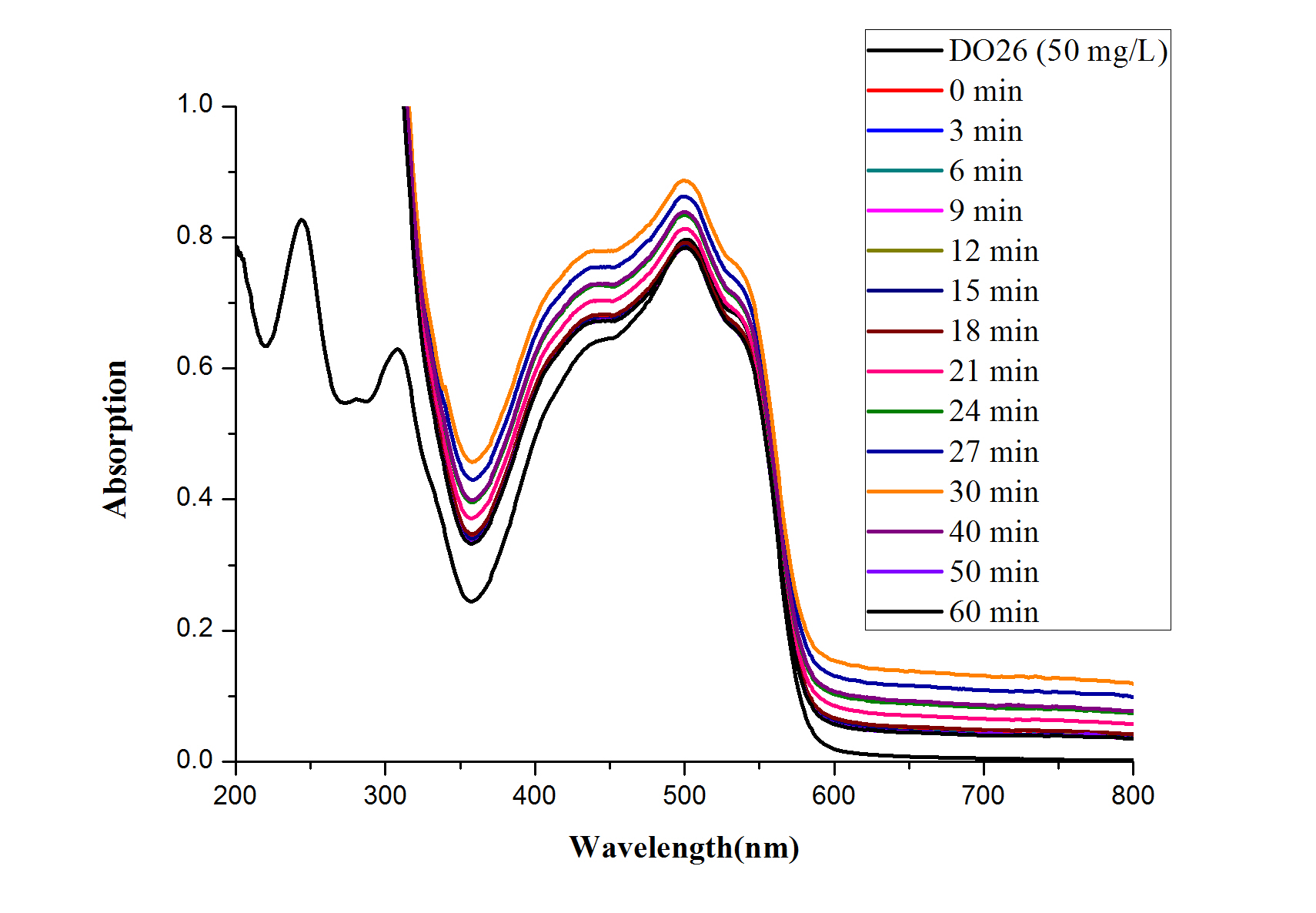

Supplement: S1 File — (ZIP) [file pone.0271408.s001.zip › supporting informations-PONE-D-22-11812/Figs5-12/DO26/fresh extract/11f 3mLDO26+0.1mL╨┬╧╩╔·╜¬╞ñ┤╝╠ß╚í╬∩-.jpg]
